# The anthropogenic fingerprint on emerging infectious diseases

**DOI:** 10.1038/s41586-026-11058-6

**Published:** 2026-09-23

**Authors:** Rory Gibb, Sadie J. Ryan, David M. Pigott, Maria del Pilar Fernandez, Renata L. Muylaert, Gregory F. Albery, Daniel J. Becker, Jason K. Blackburn, Hernan Caceres-Escobar, Michael Celone, Evan A. Eskew, Hannah K. Frank, Barbara A. Han, Erin N. Hulland, Kate E. Jones, Rebecca Katz, Adam Kucharski, Direk Limmathurotsakul, Catherine A. Lippi, Joshua Longbottom, Juan Fernando Martinez, Jane P. Messina, Elaine O. Nsoesie, David W. Redding, Daniel Romero-Alvarez, Boris V. Schmid, Stephanie N. Seifert, Anabel Sinchi, Christopher H. Trisos, Michelle Wille, Colin J. Carlson

**Affiliations:** 1 Department of Genetics, Evolution and Environment, University College London, London, UK; 2 Department of Geography, Quantitative Disease Ecology and Conservation (QDEC) Laboratory, University of Florida, Gainesville, FL USA; 3 Emerging Pathogens Institute, University of Florida, Gainesville, FL USA; 4 College of Life Sciences, University of KwaZulu Natal, Durban, South Africa; 5 Department of Health Metrics Sciences, University of Washington, Seattle, WA USA; 6 Paul G. Allen School for Global Health, Washington State University, Pullman, WA USA; 7 Sydney School of Veterinary Science, The University of Sydney, Sydney, New South Wales Australia; 8 School of Natural Sciences, Trinity College Dublin, Dublin, Ireland; 9 School of Biological Sciences, University of Oklahoma, Norman, OK USA; 10 Department of Geography, Spatial Epidemiology and Ecology Research (SEER) Laboratory, University of Florida, Gainesville, FL USA; 11 Facultad de Medicina Veterinaria y Agronomía, Campus Providencia, Universidad de Las Américas, Santiago, Chile; 12 Institute for Health Metrics and Evaluation, University of Washington, Seattle, WA USA; 13 Institute for Interdisciplinary Data Sciences, University of Idaho, Moscow, ID USA; 14 Department of Ecology and Evolutionary Biology, Tulane University, New Orleans, LA USA; 15 Cary Institute of Ecosystem Studies, Millbrook, NY USA; 16 Center for Global Health Science and Security, Georgetown University, Washington, DC USA; 17 Centre for Mathematical Modelling of Infectious Diseases, London School of Hygiene and Tropical Medicine, London, UK; 18 Mahidol-Oxford Tropical Medicine Research Unit (MORU), Faculty of Tropical Medicine, Mahidol University, Bangkok, Thailand; 19 Centre for Tropical Medicine and Global Health, Nuffield Department of Medicine, University of Oxford, Oxford, UK; 20 Department of Vector Biology, Liverpool School of Tropical Medicine, Liverpool, UK; 21 Center for Integrated Earth System Information (CIESIN), Columbia Climate School, Columbia University, New York, NY USA; 22 School of Geography and Environment, University of Oxford, Oxford, UK; 23 Oxford School of Global and Area Studies, University of Oxford, Oxford, UK; 24 Department of Global Health, Boston University School of Public Health, Boston, MA USA; 25 Science Department, Natural History Museum, London, UK; 26 Research Group of Emerging and Neglected Diseases, Ecoepidemiology and Biodiversity, Health Science Faculty, Universidad Internacional SEK (UISEK), Quito, Ecuador; 27 Instituto Nacional de Biodiversidad (INABIO), Quito, Ecuador; 28 Department of Animal Sciences, Infectious Disease Epidemiology, Wageningen University and Research, Wageningen, the Netherlands; 29 Instituto Nacional de Enfermedades Virales Humanas Dr. Julio I. Maiztegui, Administración Nacional de Laboratorios e Institutos de Salud (ANLIS), Pergamino, Argentina; 30 African Synthesis Centre for Climate Change, Environment and Development (ASCEND), University of Cape Town, Cape Town, South Africa; 31 Climate Risk Lab, African Climate and Development Initiative, University of Cape Town, Cape Town, South Africa; 32 Department of Microbiology and Immunology, Peter Doherty Institute for Infection and Immunity, Centre for Pathogen Genomics, University of Melbourne, Melbourne, Victoria Australia; 33 Department of Epidemiology of Microbial Diseases, Yale University School of Public Health, New Haven, CT USA; 34 Department of Biology, Georgetown University, Washington, DC USA

**Keywords:** Ecological epidemiology, Viral infection, Pathogens

## Abstract

Emerging infectious diseases are a hallmark of the Anthropocene^1,2^. Human-driven ecosystem changes and human–wildlife–livestock contact can drive the emergence of zoonotic and vector-borne diseases^3–5^, but how much these processes shape landscapes of outbreak risk is poorly understood, beyond a few well-studied systems^6–8^. Here we consolidate 58,319 outbreak event records for 32 diseases and systematically test how 16 hypothesized social and environmental drivers impact outbreak geographies, while accounting for detection and reporting biases. We show that outbreak risks are typically highest in mosaic landscapes where people and livestock live alongside forests and fragmented ecosystems. These combined factors, along with long-term declines in precipitation, share strong impacts across several vector-borne diseases (for example, dengue, Lyme disease and zoonotic arboviruses). By contrast, directly transmitted zoonoses (for example, Ebola and mpox) share few common drivers, and the impacts of most other anthropogenic pressures (for example, deforestation, climate warming and agricultural intensification) vary widely between diseases. Most consistently, the observed geography of outbreaks is shaped by healthcare access: reporting declines by a median of 32% (range across diseases: 1.2–96.7%) for each additional hour’s travel time from a health facility. Our findings underscore that infectious disease spillover and emergence are multi-causal, and that no one-size-fits-all strategy can prevent epidemics and pandemics. Ecosystem-based public health interventions^9^ should always follow system-specific evidence, and be paired with greater investment in health systems and One Health pathogen surveillance.

## Abstract (web summary)

Emerging infectious disease outbreak risks are ubiquitous worldwide, and highest in landscapes where people, livestock and fragmented natural ecosystems coexist, but detection and data on outbreaks are currently limited by inequalities in healthcare access.

## Main

In recent decades, emerging infectious diseases transmitted by wildlife (zoonoses; for example, COVID-19, Ebola virus disease, influenza and mpox) or arthropod vectors (for example, dengue fever, Lyme disease and Zika virus disease) have had catastrophic social, economic and ecological impacts. This trend is widely considered the result of an ongoing state shift in the biosphere^10,11^, where human-driven environmental changes are both increasing animal susceptibility to infection, and creating more opportunities for animal-to-human transmission (zoonotic spillover^2^), leading to more outbreaks of both familiar and new pathogens. The rising tide of emerging infections has brought global attention to ecological and social interventions that could mitigate the upstream drivers of disease emergence^9^. Recent attention has often focused on curbing wildlife trade or deforestation^12–15^, but other important options include reducing greenhouse gas emissions to limit climate change, ecosystem restoration initiatives, human and livestock vaccination, improved access to point-of-care diagnostics and clinical care, the development of ‘One Health’ disease surveillance systems and workforces, and stricter biosecurity practices^16,17^. These interventions are grounded in public health and ecological first principles, but there is limited scientific consensus on their potential benefits and relative priority, in large part owing to insufficient evidence about the universality of many drivers of disease transmission and emergence.

Meta-analyses and literature syntheses have shown predictable anthropogenic effects on community diversity, pathogen diversity and infection dynamics in wildlife hosts and arthropod vectors^18–22^. These studies suggest that ecosystem degradation and biodiversity loss tend to increase wildlife disease prevalence^4,18^, but that the net impacts of habitat fragmentation, agriculture, urbanization, deforestation and climate change may be unpredictable and context-specific^19,23,24^. This uncertainty reflects a mixture of scientific evidence gaps and true heterogeneity across systems, driven by differences in pathogen life cycles, host and vector ecology, and the type and intensity of anthropogenic disruption. Downstream relationships between human infectious disease risk and climate change, biodiversity loss or land use may therefore be similarly idiosyncratic across diseases and regions^25^. These relationships are further complicated by exposure processes: human–wildlife contact patterns and social vulnerabilities to outbreaks vary between landscapes and populations, but are rarely captured in wildlife-focused studies^26^.

Several influential studies have shown that geographic hotspots of human disease emergence events (that is, the location and circumstances of the first scientifically documented outbreaks of around 300 pathogens) correlate with socio-ecological factors including land use change, agricultural expansion, biodiversity hotspots and global travel^3,27^. Other research has identified similar drivers of historically important outbreaks through literature review or expert opinion^5,28^. However, the circumstances of the first confirmed human infections, or of unusually large outbreaks, may not reflect the social and ecological factors that determine wider landscapes of infection risk. For example, data on emergence events are inherently biased towards better-resourced settings where novel pathogens can be identified more easily, and away from rural and marginalized populations that experience an endemic burden of zoonotic and vector-borne diseases^29^ (including many infections typically framed as ‘emerging’^30^). The growing availability of comprehensive georeferenced outbreak datasets for many of these diseases—compiled from disease surveillance reports, scientific and grey literature—now provides the opportunity for a more systematic, global, data-driven assessment of emerging disease drivers.

**Fig. 1:**
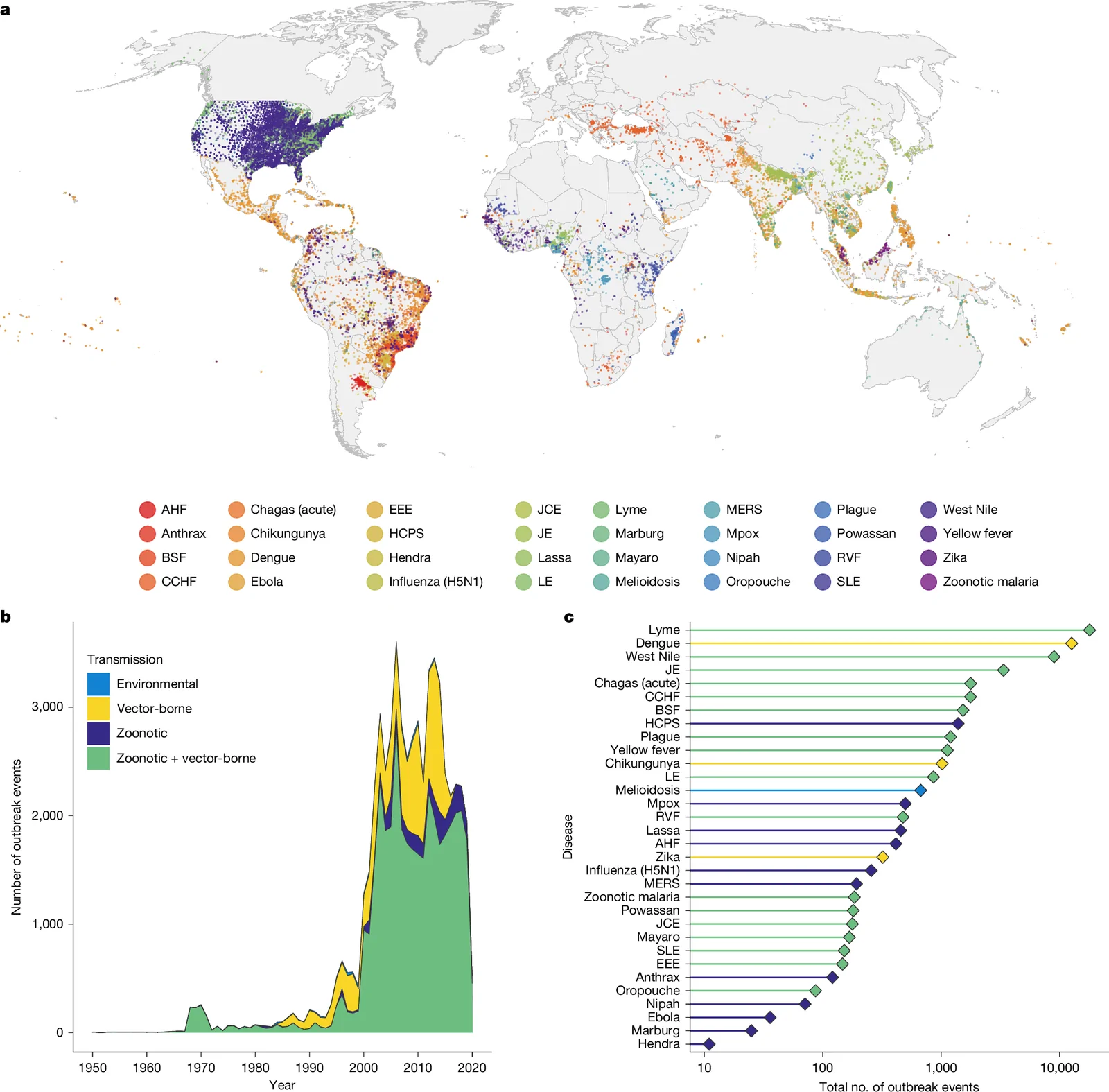
A global compendium of outbreak events for 32 human emerging infectious diseases. Records include a mix of georeferenced human disease occurrence or outbreak data and case incidence data from national surveillance systems (Extended Data Table 1). **a**, Each point represents an outbreak event (at least one confirmed case per named locality per year; *n* = 58,319) for diseases whose predominant human infection routes are broadly classified as either zoonotic (for example, Ebola virus disease, Lassa fever), zoonotic and vector-borne (for example, West Nile fever, yellow fever), vector-borne and maintained mainly in human hosts (for example, dengue, chikungunya and Zika virus disease) or environmentally transmitted (melioidosis). **b**,**c**, Data were predominantly from after 2002 across all transmission routes (data shown from 1950 onward, **b**) with most data available for well-monitored widespread diseases (for example, Lyme disease, dengue, West Nile fever) and least for emerging bat-borne infections (filo- and henipaviruses) (**c**). AHF, Argentine haemorrhagic fever; BSF, Brazilian spotted fever; EEE, Eastern equine encephalitis; HCPS, hantavirus cardiopulmonary syndrome; JCE, Jamestown Canyon encephalitis; JE, Japanese encephalitis; LE, La Crosse encephalitis; SLE, St. Louis encephalitis. For brevity, we omit ‘disease’, ‘virus disease’ and ‘fever’ from disease names as appropriate; Extended Data Table 1. Base map in **a** from Natural Earth (https://www.naturalearthdata.com).

In this study we harmonized georeferenced human outbreak data sources for 32 emerging infectious diseases (Fig. 1, Extended Data Table 1 and Supplementary Table 1), including bat viruses with epidemic and pandemic potential (filo-, henipa- and coronaviruses), rodent-borne pathogens (including hanta- and arenaviruses, plague and mpox), mosquito-borne arboviruses (including flavi-, alpha- and orthobunyaviruses such as dengue, chikungunya and Rift Valley fever (RVF)) and other neglected zoonotic, vector-borne and environmentally transmitted infections (including melioidosis, Crimean–Congo haemorrhagic fever (CCHF) and *Plasmodium knowlesi* zoonotic malaria). Our focus was on drivers of outbreak risk, rather than the distinct factors that predispose outbreaks to become epidemics or pandemics, so we included only outbreak records associated with some level of environmental influence: this included all records for vector-borne and environmentally transmitted infections, but only index cases with a probable zoonotic origin for diseases with substantial onward human-to-human transmission (for example, Ebola virus disease, mpox; Methods). Data were collated from published scientific datasets and national notifiable disease surveillance systems from the continental USA, Brazil and Argentina (Methods). The complete dataset includes 58,319 unique outbreak events across 169 countries (Fig. 1a and Methods) from 1910 to 2022 (but primarily after 2000; Fig. 1b), with outbreak events defined as at least one confirmed case at a given georeferenced location in a given year (either point or administrative polygon; Methods). Most source datasets were relatively comprehensive geographically, so our harmonized database has extensive coverage across the Americas, sub-Saharan Africa and South and Southeast Asia, but fewer records in North Africa and above the 50° N latitude line.

**Extended Data Fig. 1:**
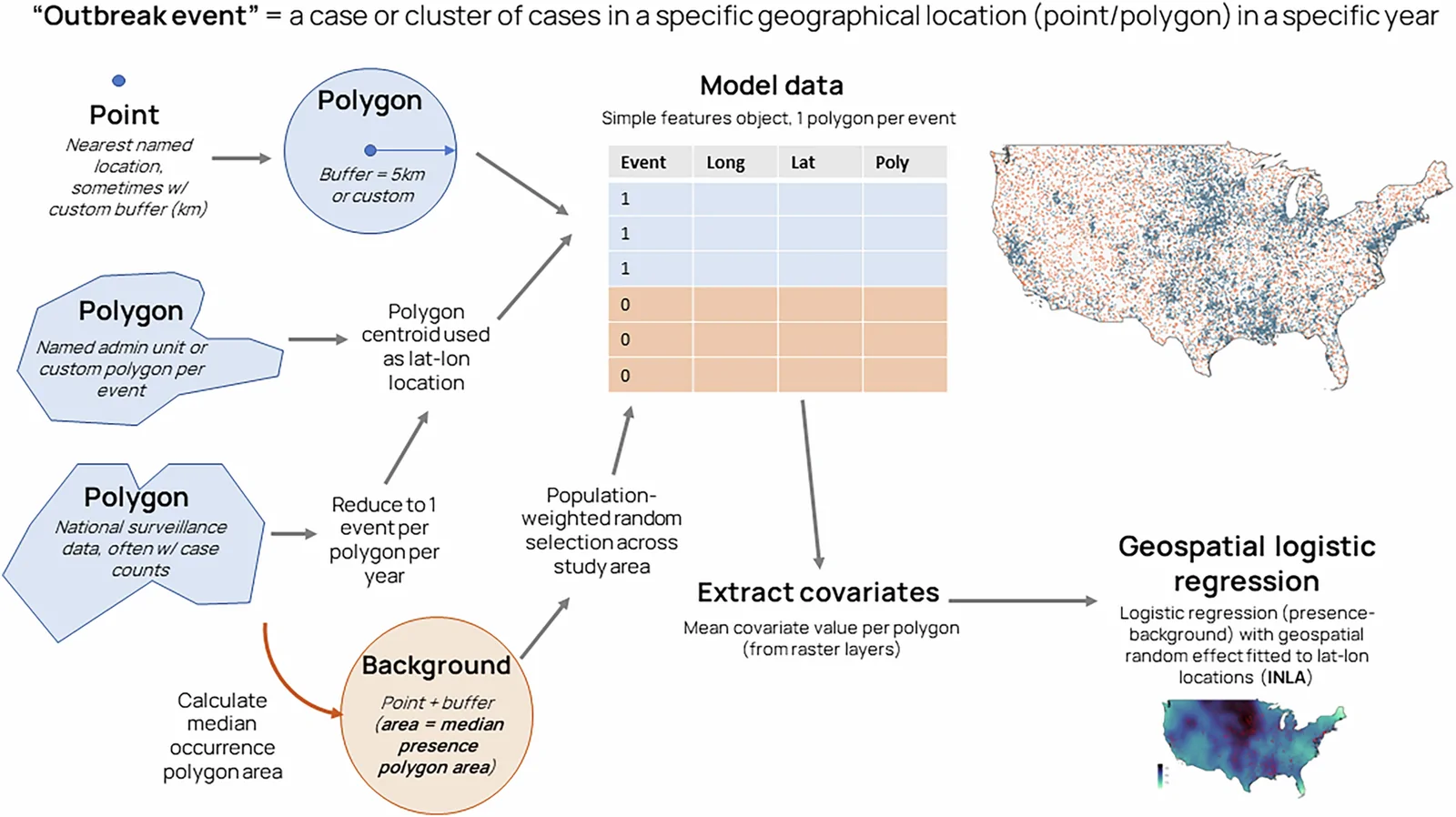
Bringing diverse disease case and outbreak data sources into a common analytical framework. Disease data sources included georeferenced case or outbreak event locations in point format (nearest named location), case or outbreak event occurrences within named administrative polygons, and case surveillance data at administrative polygon levels from national surveillance systems (sources variously shown in blue). Each contains different information about transmission intensity and different levels of geographical precision, which necessitated bringing different data types into a common, standardized analytical framework, shown in this figure. All outbreak locations (whether natively point or polygon) were converted into polygons (blue) and any polygons covering too large a spatial area were excluded as too imprecise (typically > 5000 km^2^, but up to 20,000 km^2^ for some data-deficient diseases as a compromise to retain as much data as possible; Methods). Background points were generated across the study area weighted by population (Methods, Extended Data Fig. 2, map shown is for West Nile fever), then buffers were created around background locations to cover the same median area as the presence locations (orange), to ensure covariates were averaged across a comparable spatial area for both occurrence points and polygons and background locations. For each polygon the mean value of each raster covariate was calculated across the entire polygon, and used as input to geospatial logistic regression models.

**Extended Data Fig. 2:**
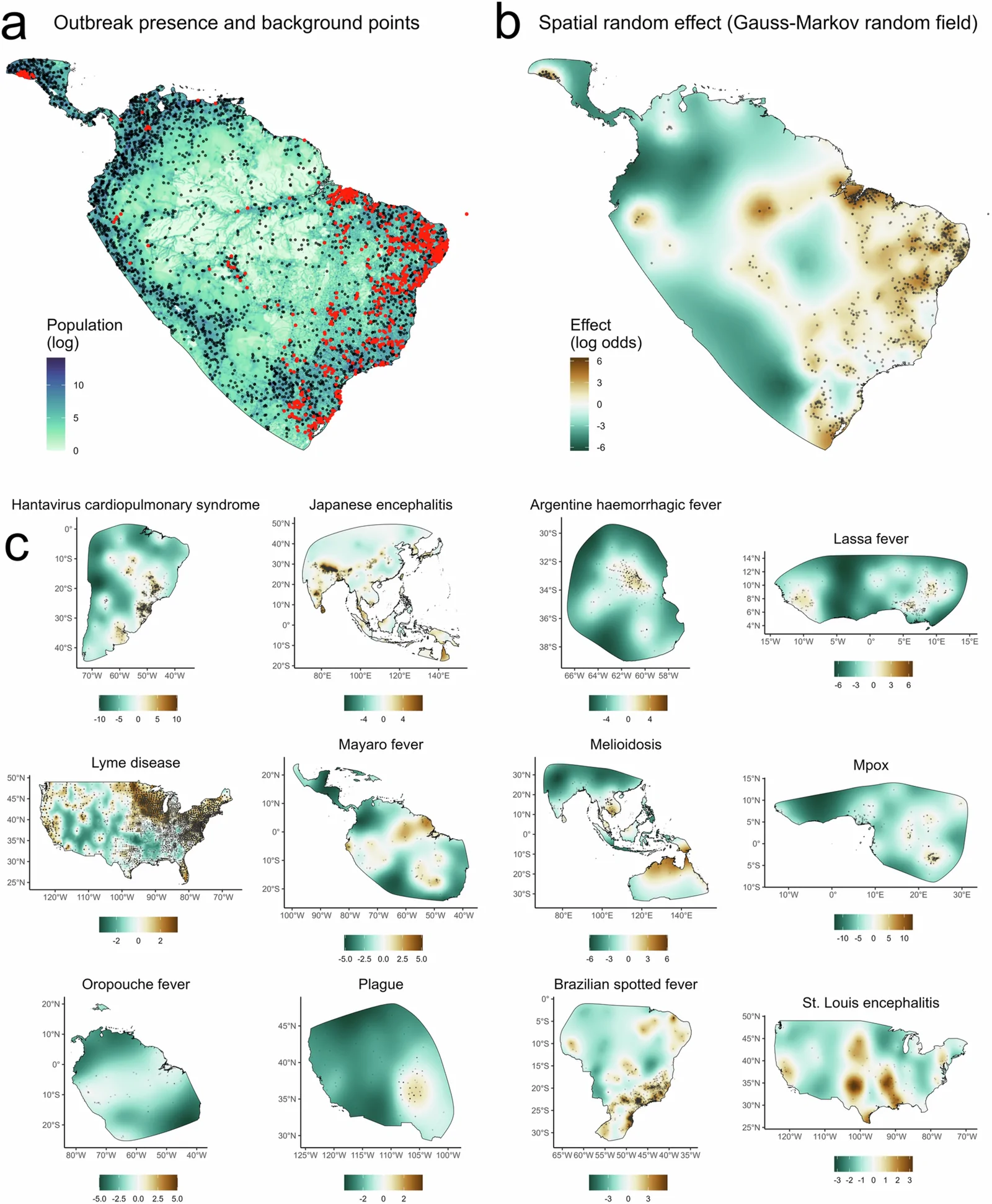
Case-control and geospatial model design for a subset of diseases. Geospatial logistic regression models were fitted to estimate the effect of covariates on the log odds of outbreak event occurrence (red points). The top row shows an example of model design for acute Chagas disease in Central and South America. Since outbreaks are presence-only data, we generated background points by randomly sampling 1 km grid cell locations across the study area (black border) weighted by log human population (left panel; shown as black points) to create a pseudo case-control design (i.e. comparing socio-environmental conditions at outbreak locations to the background distribution of conditions experienced by human populations overall) (A). To account for unmeasured factors shaping broad-scale outbreak geographies, models included a continuous geospatial random effect (Gauss-Markov random field; fitted field for Chagas disease is shown in top right panel) (B). Additional subpanels show fitted geospatial effects from the hypothesis-driven (“top ranked”) models for 12 randomly-selected diseases (C). Shading denotes the marginal contribution to outbreak risk (log odds scale), with brown denoting higher risk, and green denoting lower risk. Observed outbreak event locations are overlaid as gray points.

Using this dataset, we developed a standardized framework for inferring the socio-environmental drivers of spatial outbreak intensity (Extended Data Fig. 1 and Methods). We use a modified case-control design^31^, which compares contemporary (after 1980) outbreak locations with population-weighted background locations (Extended Data Fig. 2 and Methods). At each location we extracted 16 covariates from gridded geospatial datasets (Extended Data Table 2), which fall under five broad categories: detection processes (motorized travel time to healthcare; urban land cover), socioeconomic factors (livestock density; relative social vulnerability), ecosystem structure (spatial vegetation heterogeneity as an indicator of landscape fragmentation; forest cover; cropland cover; biodiversity intactness index), land use change and intensity (forest loss; cropland expansion; urban expansion; mining; protected area coverage; hunting pressure index) and climate change (change in mean annual temperature and precipitation between a 1950–1970 reference period and 2000–2020). Most covariates were derived from gridded remote sensing products, climate reanalysis and socio-demographic data, but a few necessarily came from composite or modelled products (biodiversity intactness, social vulnerability and hunting pressure; Methods). We used Bayesian geospatial logistic regression models to test the linear contribution of these covariates to outbreak risk. Models were fitted to outbreak event records from 1985 onwards to align with the timescales of covariate data (except certain data-sparse diseases that included records from 1980 onwards; Methods). All models included a continuous spatially structured random intercept, to account for macro-scale unexplained variation in both outbreak risk and data coverage (for example, owing to differing reporting rates between countries or regions; Extended Data Fig. 2 and Methods). We applied this framework first to the entire dataset, and then on a disease-by-disease basis, and asked whether (1) a general anthropogenic fingerprint on the spatial intensity of outbreak events can be distinguished from bias and noise; (2) certain forms of environmental change are implicated consistently in outbreak risks across a range of pathogen types, transmission modes and regions; and (3) there is evidence of widely shared drivers across diseases that could point towards promising ecosystem-based intervention strategies.

## Outbreak hotspots are shaped by detection biases

**Fig. 2:**
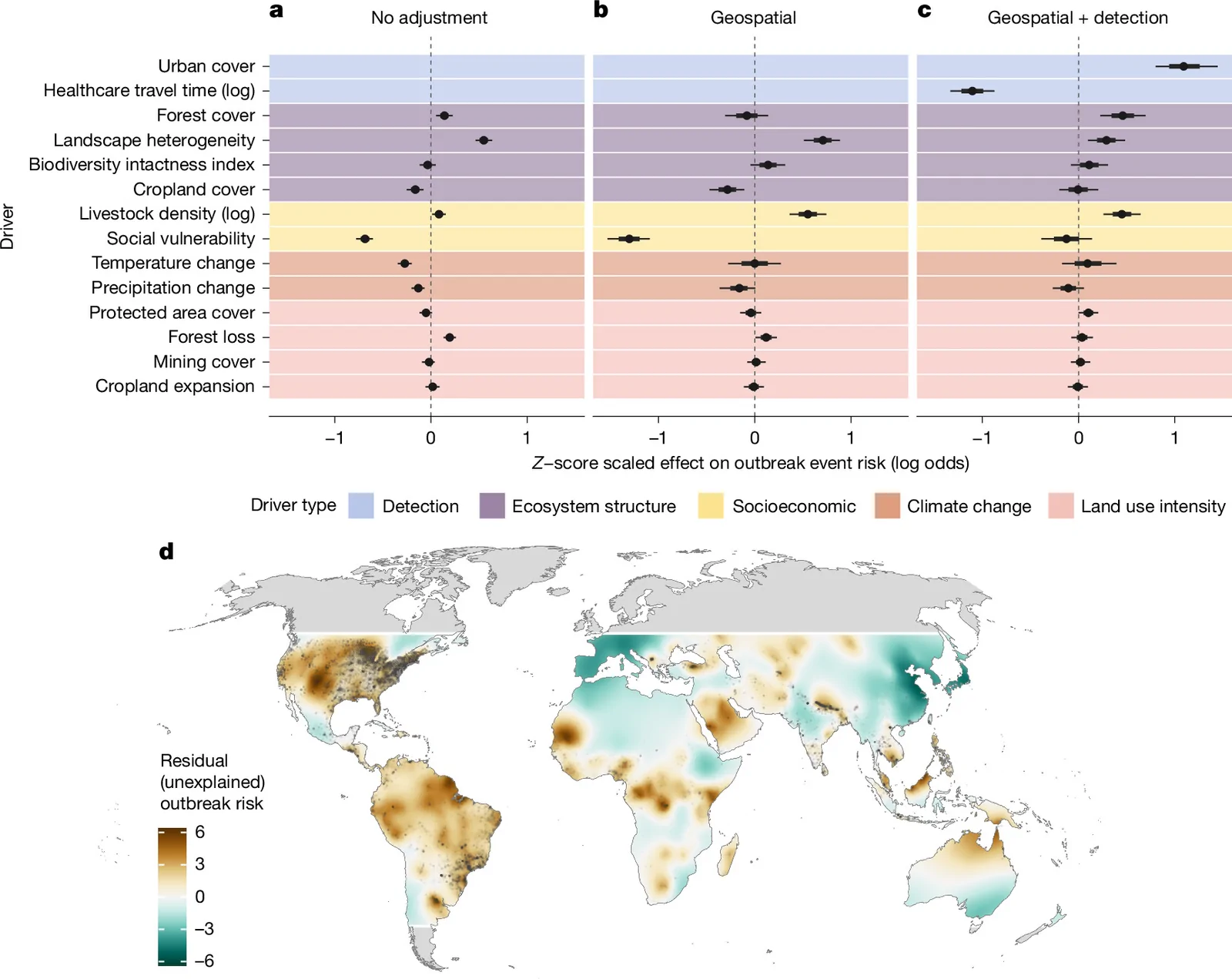
Geographical reporting and detection biases confound inference of global emerging infectious disease drivers. **a**–**c**, Points, and wide and narrow error bars show the linear fixed effects of driver covariates on outbreak event risk across all diseases (posterior marginal median, 67% and 95% credible interval). Each posterior distribution was estimated across an ensemble of 100 Bayesian logistic regression submodels fitted to randomized subsamples of the full dataset (*n* = 49,239 outbreak events between 1985 and 2022 and 50,000 background points) containing equal sample sizes for each disease (Methods). Slope estimates denote the marginal effect of each *z*-score scaled covariate on spatial outbreak risk, and background shading denotes driver type. Panels denote model specification: including either only socio-environmental fixed effects (no adjustment, that is, excluding local detection covariates, **a**), adding a continuous geospatial random effect (geospatial, **b**) or adding a geospatial effect and local detection covariates (geospatial + detection, **c**). **d**, The geospatial random effect (Gauss–Markov random field) reflects residual (unexplained) spatial variation in observed outbreak event risk, including due to macro-scale sampling processes such as biases in awareness and reporting. Map colour scale denotes contribution to the observed pattern of outbreak events (log odds scale), calculated as the mean over 100 submodels (geospatial + detection model), with outbreak locations overlaid as translucent points. The geospatial effect was inferred only within the latitudinal range of available data; areas outside these bounds are shaded in grey. Inferred effects were very similar for models fitted to subsets of diseases with different transmission characteristics (either zoonotic or vector-borne) and broadly similar across geographic regions (Extended Data Fig. 4). Covariates for urban expansion and hunting pressure were excluded owing to high collinearity and lack of global coverage, respectively. Base map in **d** from Natural Earth (https://www.naturalearthdata.com). Source data

First, we estimated the overall effects of anthropogenic drivers across all diseases by fitting models to the full dataset (*n* = 49,239 after data preprocessing, with 50,000 background points). To ensure each disease contributed equally to inference despite wide variation in sample size (Fig. 1), all marginal driver effects were estimated across an ensemble of submodels fitted to balanced subsamples of the data (Methods). Before adjustment for geographic reporting processes, outbreak events were correlated with human-impacted ecosystems (higher forest cover, fragmentation and deforestation) and less socially vulnerable communities (Fig. 2a). However, these associations could be confounded by multi-scale spatial biases in outbreak detection, investigation and reporting. Extending the model to include a geospatial random effect and adjust for detection-related covariates demonstrated that apparent hotspots are created primarily by observation and reporting processes (Fig. 2b,c). In many regions, the unexplained spatial variation captured by the geospatial effect exceeds the size of all covariate effects, particularly in the USA and Brazil (whose national disease surveillance systems are represented substantially in our data) and in data hotspots across West and Central Africa, the Middle East, and South and Southeast Asia (Fig. 2d). At a more local scale, outbreak events are reported much more frequently in cities and near health centres, with these two effect sizes much larger than any other covariate effects (Fig. 2c). These relationships probably reflect primarily the importance of accessible and better-resourced health systems infrastructure (for example, clinics, diagnostic laboratories) in determining whether outbreaks are detected and reported.

**Extended Data Fig. 3:**
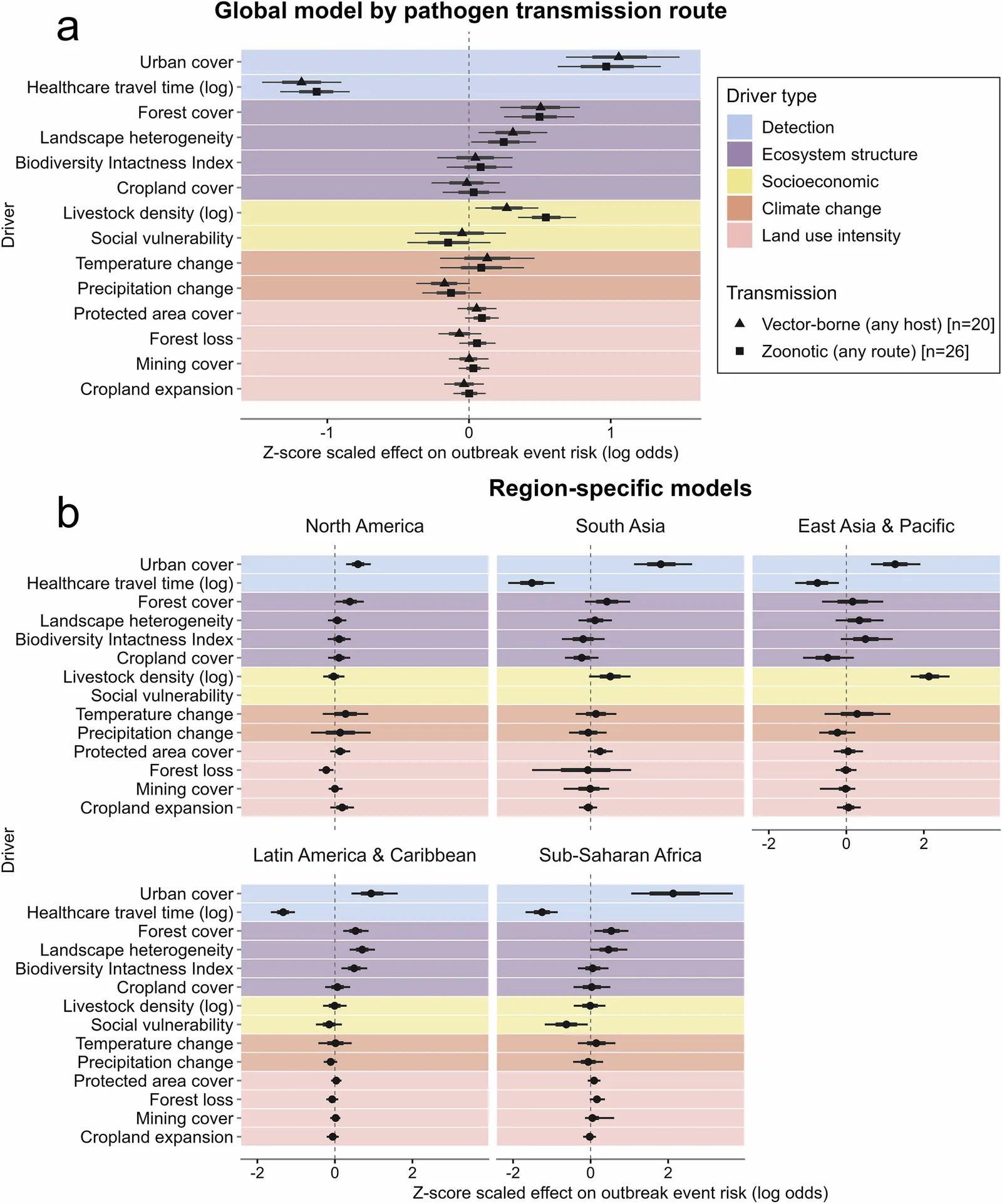
Global drivers of emerging disease outbreaks across different transmission groups and regions. Points and error bars show linear fixed effects of scaled covariates (posterior marginal median, 67% and 95% credible interval) estimated across an ensemble of 100 Bayesian geospatial logistic regression models fitted to balanced subsamples of the dataset (Methods). Slope estimates denote the effect of each scaled covariate on spatial outbreak risk. Replicating the analysis of Fig. 2, (A) shows global models fitted separately for groups of diseases defined non-exclusively as either zoonotic (non-human animal reservoir with any mode of transmission; n = 26 diseases, 36,577 outbreak points) or vector-borne (transmitted by invertebrate vectors regardless of host, i.e. including principally anthroponotic arboviruses such as dengue; n = 20 diseases, 45,556 points). Point shape denotes transmission type. (B) shows the results of models separately fitted to data from 5 different global subregions with sufficient data coverage: North America (n = 26,640), Latin America and the Caribbean (n = 10,767), South Asia (n = 3,351), Sub-Saharan Africa (n = 1,799) and East Asia and Pacific (n = 4,521) (Extended Data Fig. 3). Certain variables were excluded from the region-specific models due to multicollinearity at the regional-level (e.g. healthcare travel time for N. America; social vulnerability for N. America, South and East Asia).

**Extended Data Fig. 4:**
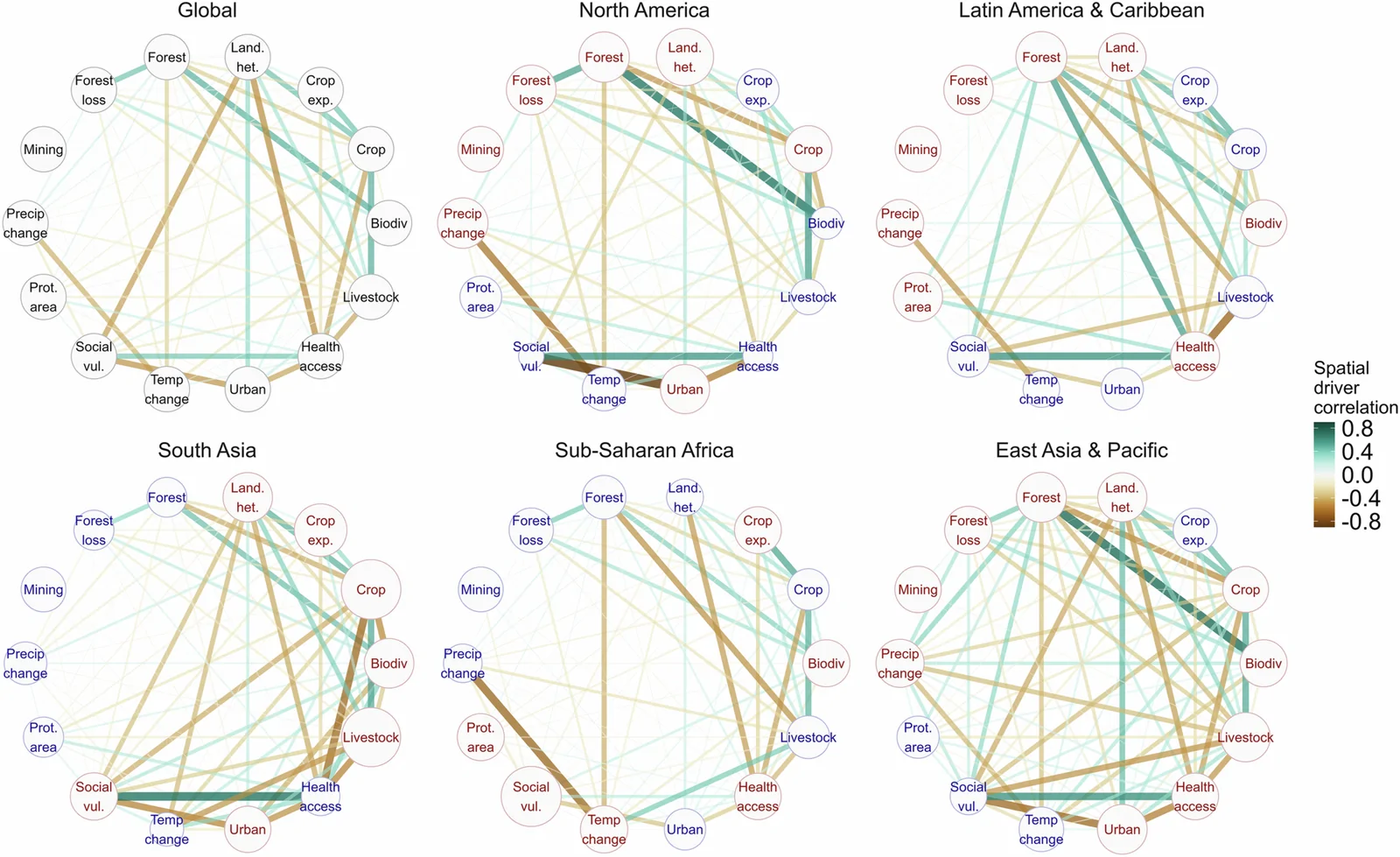
The nexus of emerging infectious disease drivers differs between geographic regions. Networks show pairwise Pearson correlations between all driver covariates (nodes), with edge colour showing direction and strength of correlation (positive in green, negative in brown) and edge weight denoting strength of correlation (i.e. absolute value). Correlations were calculated based on 50,000 population-weighted background points generated across the global study area (bounding box around all outbreak occurrences; Methods), with covariate values averaged across a 10 km radius buffer around each point. Networks are shown using all background points (global) and separately for the five subregions containing most of the outbreak data. To visualize regional differences in covariate intensity per region, node sizes in region-specific networks are proportional to each covariate’s mean scaled value, with node text colour denoting whether this was above (red) or below (blue) the global average (for example, North America has substantially lower mean social vulnerability than the global average across all points, and sub-Saharan Africa and South Asia substantially higher). Urban expansion was excluded as it was consistently highly correlated with urban cover (*ρ* > 0.85), and hunting was excluded as its restriction to tropical forest biomes resulted in a high proportion of missing values. Most variable pairs were uncorrelated or weakly correlated (mean 13% of driver pairs with absolute *ρ* > 0.5, and 8% with absolute *ρ* > 0.7, across all regions).

Social and ecological risk factors had substantially modified effect sizes with wider uncertainty after adjusting for detection and reporting processes. Outbreak event risk increased with higher levels of forest cover, landscape fragmentation and livestock density (Fig. 2c). These results were very similar when only including diseases classed non-exclusively as either zoonotic (human infections principally arising from spillover from an animal reservoir; *n* = 26 diseases) or vector-borne (transmitted by invertebrate vectors regardless of host type; *n* = 20; Extended Data Fig. 3a). These findings align broadly with the consensus that infectious diseases tend to emerge in zones of frequent contact among people, livestock and wildlife^3^. However, the intensity and spatial clustering of human-driven impacts varies geographically, which could produce distinct regional syndromes of outbreak risk: for example, in East Asia and the Pacific, most anthropogenic pressures are correlated tightly in space, whereas the opposite is true in sub-Saharan Africa (Extended Data Fig. 4). The directionality of inferred drivers was mostly consistent when fitting separate models for five principal geographic regions, but with notable variations in effect size; for example, impacts of livestock density were strongest in Asia (Extended Data Fig. 3b). These geographic differences in inferred drivers will be influenced by each region’s particular set of diseases, nexus of anthropogenic pressures and reporting processes. Unpacking these requires shifting towards more granular, disease system-specific inference.

## Outbreak drivers differ between disease systems

**Extended Data Fig. 5:**
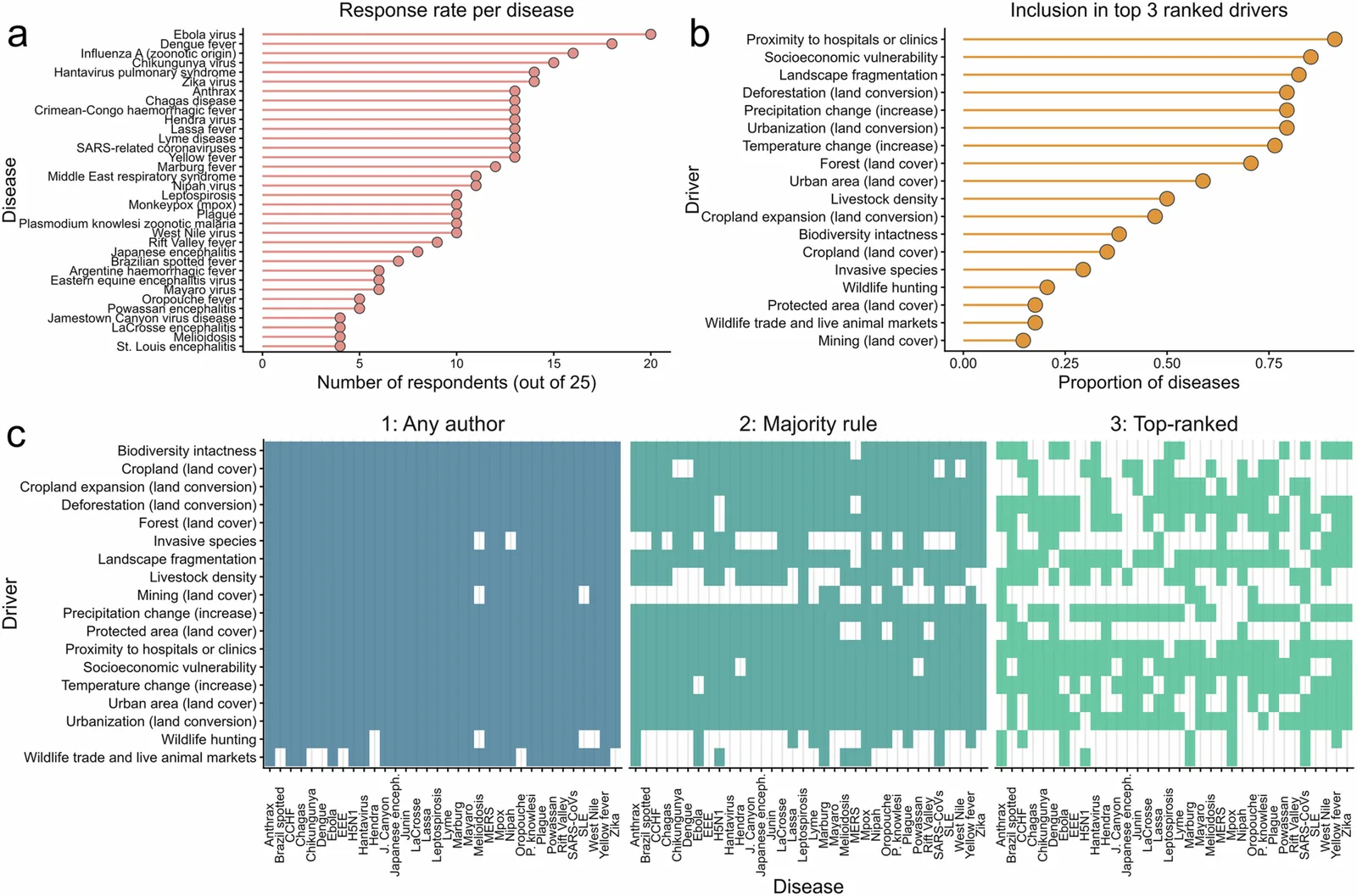
Hypothesised socio-environmental drivers for emerging infectious diseases from a team hypothesis-generation exercise. To ensure our analyses tested appropriate, ecologically-plausible drivers for each disease, we used a structured form-based hypothesis exercise completed by most coauthors (n = 25 out of 31; Methods). Authors had the option to either fill in the form or leave blank for each disease (disease names provided were as in panel A). There was substantial variability in response rates (A), with most responses for better-studied or widespread diseases (e.g. Ebola, dengue, influenza A) and vice versa. Authors ranked each driver effect as “positive”, “negative”, “none” or “don’t know” and additionally were asked to select the top 3 most important drivers for each disease. Health systems access and socioeconomic vulnerability were the most commonly top-ranked drivers, followed by fragmentation, deforestation, urbanization and climate change (B; shows the proportion of diseases for which each driver was ranked in the top 3 by at least 1 respondent). Bottom panels (C) show hypothesized drivers to test for each disease based on three schemes: “any author” (drivers that were selected by at least 1 author); “majority rule” (drivers for which more authors stated any effect than no effect); and “top ranked” (all drivers that were ranked among the top 3 by at least 1 author).

**Extended Data Fig. 6:**
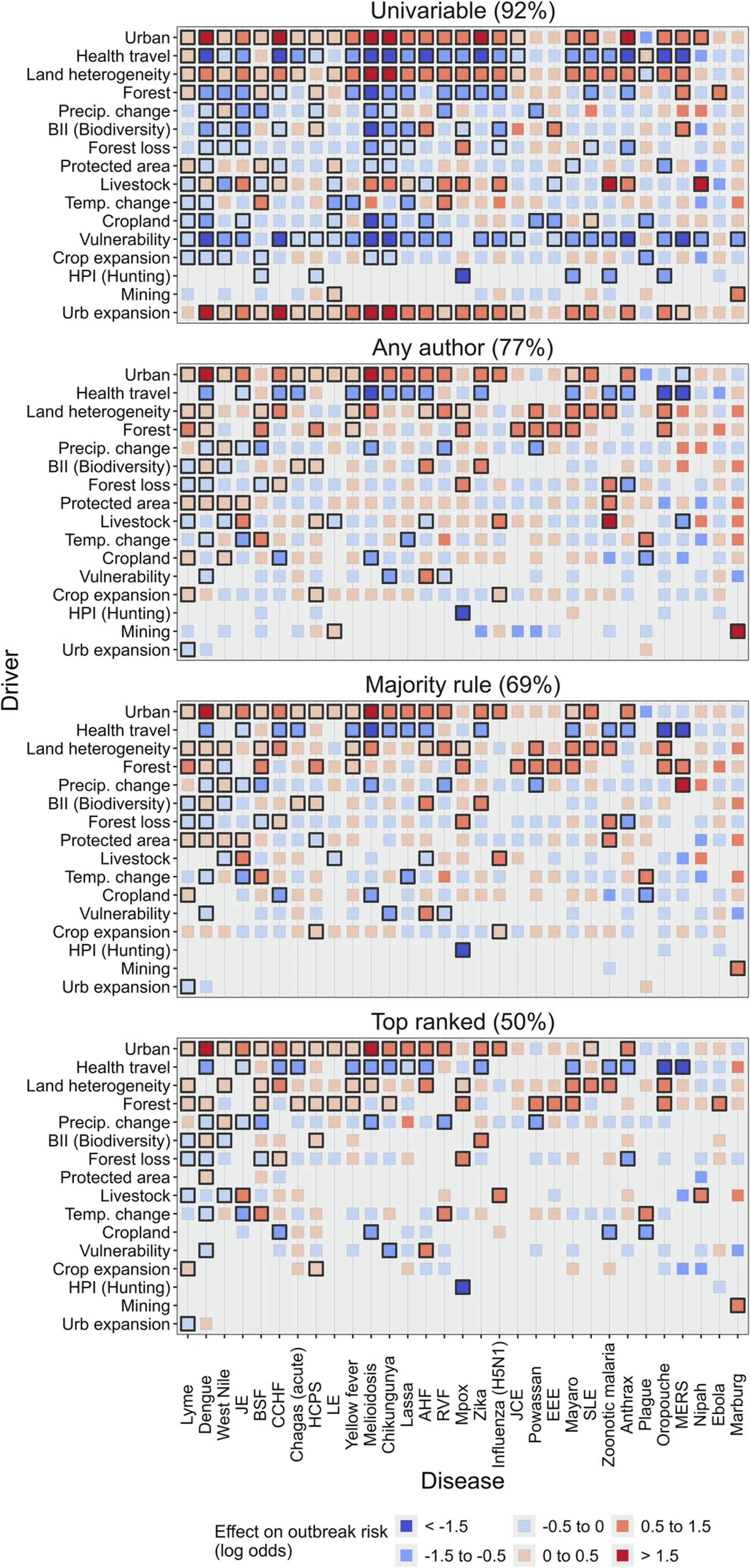
Estimated posterior mean effects of socio-environmental drivers of emerging infectious disease outbreaks, in univariable and hypothesis-driven models. Models were run in univariable driver-disease pairs (i.e. geospatial random effect plus each driver individually; top) and in multivariable models including three sets of hypothesized drivers identified through the hypothesis exercise, using the “any author”, “majority rule” and “top-ranked” criteria (Extended Data Fig. 5). Estimated fixed effects are shown as blocks, with colour scale showing the posterior mean effect of z-score scaled covariates (log odds scale), where red denotes increasing risk and blue denotes decreasing risk. Black borders denote strong evidence of a non-zero effect on risk (i.e. 95% credible interval not overlapping zero). Drivers are ranked by number of non-zero effects from the “top-ranked” models (top to bottom), and diseases are ordered from left to right by number of outbreak records (highest to lowest).

Next, we developed disease-specific geospatial models for 31 diseases (excluding Hendra virus disease owing to extreme data sparsity). The high number of pairwise disease–driver combinations (*n* = 496) and spatial reporting biases created a risk of detecting spurious relationships. Therefore, for each disease we identified a specific set of hypothesized drivers to test, through a team-wide participatory exercise to incorporate disease-specific expertise from across the study authorship. This was structured as an online form in which co-authors independently selected hypothesized drivers from a list of candidate variables (completed by 25 of 31 study authors; Supplementary Table 2 and Methods). The results of this exercise were used to select covariates to test per disease, using three different thresholds for author agreement, ranging from very broad (excluding only highly improbable drivers) to strict (testing only top-ranked covariates; Extended Data Fig. 5 and Methods). We then fitted hypothesis-driven multivariable models for each disease following the general methods described above, with geospatial effects and detection process covariates included in all models as a priori confounders (Methods). We also fitted univariable models, that is, each driver individually plus a geospatial random effect, to compare with inferred effects without adjustment for detection covariates (Extended Data Fig. 6).

**Fig. 3:**
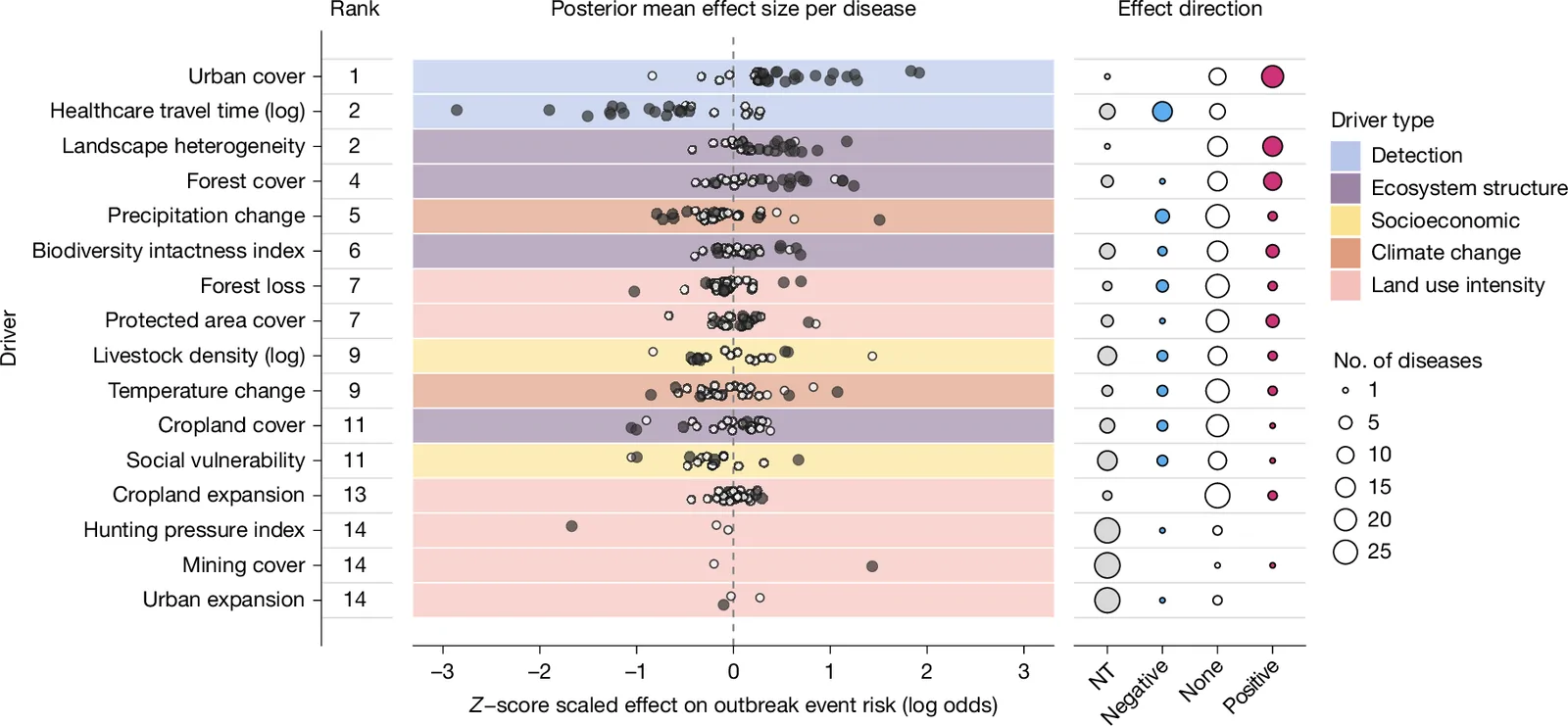
Drivers of outbreak risk are heterogeneous across 31 emerging infectious diseases. Results are summarized from separate hypothesis-based geospatial logistic regression models for 31 diseases in the dataset (Extended Data Fig. 6). Drivers are shown ranked by the number of diseases for which there was strong evidence of a relationship (95% credible interval not overlapping zero) (left column). Points (middle column) show the estimated posterior mean driver effect sizes for all tested diseases (1 point per disease). Black points, strong evidence of a relationship; white points, no strong evidence (95% credible interval overlapping zero). Background shading denotes the class of socio-environmental driver. The right hand column summarizes the directionality of effects for each driver, with point size showing the number of diseases for which relationships were either not tested (NT), negative, positive or no strong evidence (None). Results are based on hypotheses generated using a ‘majority rule’ criterion for author agreement; results for models using broader and stricter criteria are very similar (Supplementary Figs. 2 and 3). Source data

Across diseases, we again found widespread evidence of systematic reporting biases. The most prevalent significant effects were of increasing urban land cover (20 out of 30 diseases tested) and proximity to the nearest health facility (15 out of 23 tested), and these covariates also had the two largest average marginal effect sizes (Fig. 3 and Supplementary Figs. 1–3). Urban social and environmental factors can also be true contributors to elevated outbreak risk for certain diseases^32^, but the near-ubiquity of the urban effect across this ecologically diverse set of infections—including those associated primarily with rural settings, such as anthrax—strongly suggests a principal role of detection bias (Methods). For about half the diseases we examined, outbreak risk increased in more fragmented (15 out of 30 tested) and forested landscapes (13 out of 27 tested), with effect sizes that were comparable with those of detection drivers (Fig. 3). For a quarter of diseases (8 out of 31 tested), outbreak risk was linked strongly to long-term changes in annual precipitation. Notably, areas experiencing climate drying trends are at higher risk of outbreaks of several vector- and water-borne diseases with known or suspected links to hydrometeorological anomalies (for example, RVF^33^, dengue^34^, melioidosis^35^ and Japanese encephalitis^36^; Extended Data Fig. 6). This indicates that long-term drying may be increasing underlying societal or landscape susceptibility to the dynamic factors that trigger outbreaks. For example, abrupt transitions between long-term drought and extreme rainfall are implicated increasingly in explosive arboviral outbreaks, even though the precise mechanisms vary by disease (for example, domestic water storage for dengue^34^, versus floodwater mosquito breeding for RVF^33^).

Relationships with land use, biodiversity, temperature and socioeconomic factors were detected less commonly across diseases, and much more variable in effect size and direction (Fig. 3 and Extended Data Fig. 5). Although deforestation is often considered one of the primary drivers of disease emergence, we detected impacts of recent (2000–2020) cumulative forest loss in relatively few systems for which this driver was tested (6 out of 29), with a mix of directional effects (2 increasing risk, 4 decreasing risk) and mostly small effect sizes (Fig. 3). There were similarly varied relationships with biodiversity intactness, protected area coverage and livestock density (7 out of 23, 6 out of 27 and 5 out of 18, respectively; Fig. 3); the lack of a widespread positive effect of livestock density contrasts notably with the global models. Climate warming was a hypothesized driver for most diseases, but we detected effects of long-term temperature change for only a few diseases (5 out of 28 tested), again with little consistency in direction (3 increasing risk, 2 decreasing risk; Fig. 3). Notably, clearly disentangling the influence of social vulnerability from correlated detection biases proved impossible at this broad scale (and with a relatively coarse global vulnerability indicator): outbreak events were biased strongly towards wealthier settings in univariable models (25 out of 30 diseases tested), but this association was always negligible or much weaker after adjusting for detection covariates (Extended Data Fig. 6). All these findings were very similar when testing hypothesized drivers chosen using alternative criteria for author agreement, suggesting that our team-based hypothesis selection approach did not systematically bias our findings (Supplementary Figs. 2 and 3 and Methods).

**Extended Data Fig. 7:**
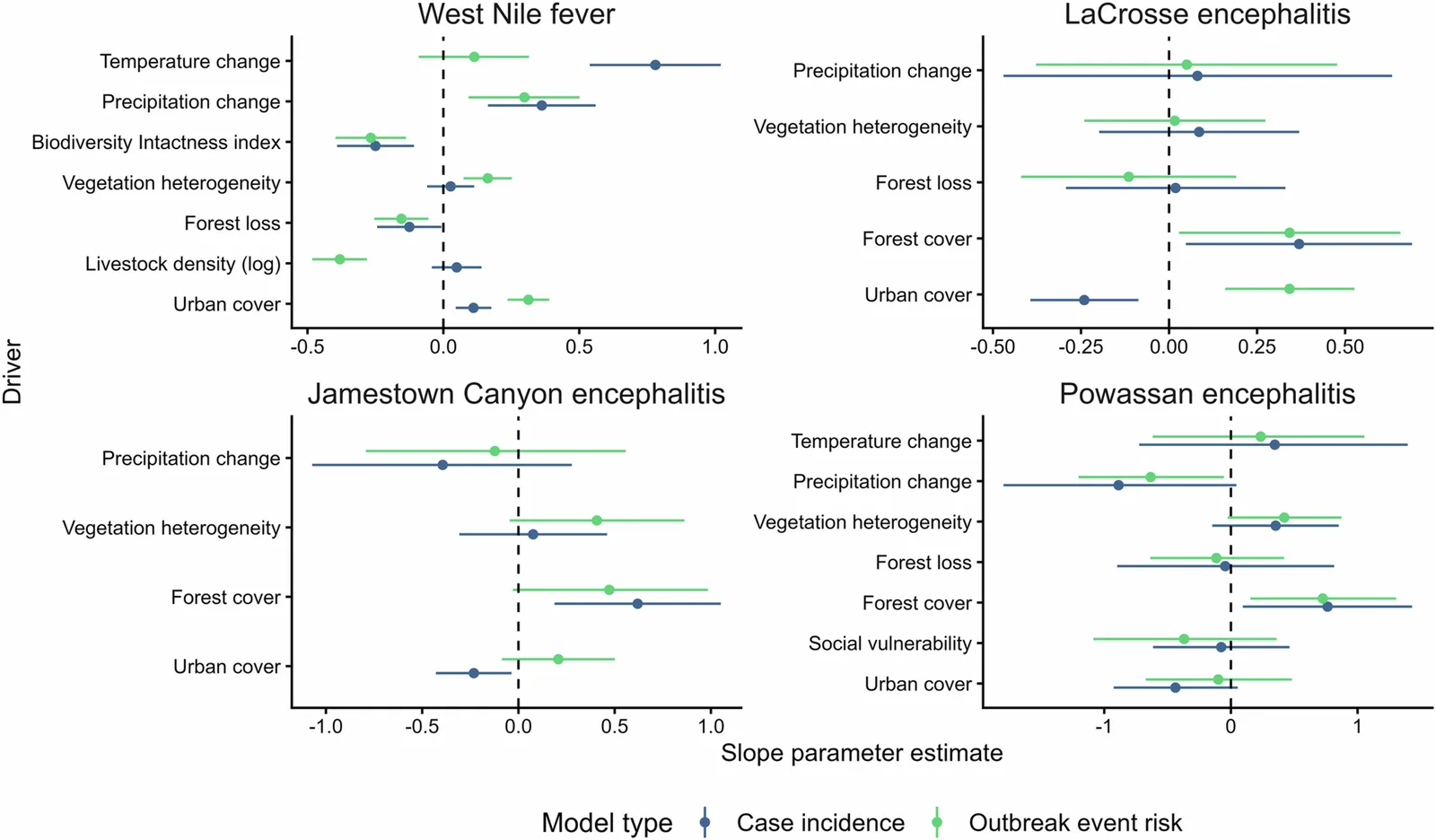
Comparison of inferred socio-environmental drivers of disease incidence and outbreak event risk for arboviruses in the USA. Points and error segments compare coefficient estimates between full geospatial models of county-level case incidence, and our outbreak event risk modeling framework (Methods), for 4 diseases with varying quantities of case incidence data from the US CDC’s ArboNET surveillance platform. Incidence slope parameters (blue) measure the inferred effects of each driver on observed log incidence (mean and 95% credible interval). These are shown alongside slope parameters from outbreak event models (green), which measure covariate effects on log odds of outbreak event occurrence compared to population-weighted background points (i.e. our standardized framework for this study; Methods). Drivers tested were based on the “top ranked” criterion in the hypothesis exercise (Extended Data Fig. 5). Data: West Nile fever (annual 2004–2020; total cases = 35,233; number of outbreak events = 1,895; total counties included in model study area = 3,084); La Crosse encephalitis (annual 2003–2020; cases = 1,369; outbreaks = 306; counties = 2,354); Jamestown Canyon encephalitis (annual 2000–2020; cases = 225; outbreaks = 112; counties = 2,642); Powassan encephalitis (annual 2004–2020; cases = 199; outbreaks = 93; counties = 1,146).

It is unclear how much the limited detectability of certain drivers reflects a true absence of causal relationships, versus a consequence of data sparsity or spatial and temporal misalignments between infection events, detection events and environmental data (Methods). These limitations meant we could not disentangle urban reporting biases from the more complex and localized influences of urban built environments, climates and population density on disease transmission^26^. Our framework is also less likely to detect other weaker, more spatially complex or dynamic drivers, such as transient changes in risk during the land conversion process^37^, especially for data-limited diseases. To some degree, these limitations may be inherent to cross-disease geospatial analyses at continental or global scales; we therefore suggest our approach should be considered complementary to system-specific work, including both longitudinal eco-epidemiological studies^6^ and ethnographic research^38^. Nonetheless, our confidence in the findings was strengthened by a sensitivity analysis of arbovirus surveillance data from the USA, which showed that our outbreak event case-control framework can detect similar spatial drivers as full models of county-level disease incidence (Extended Data Fig. 7).

**Extended Data Fig. 8:**
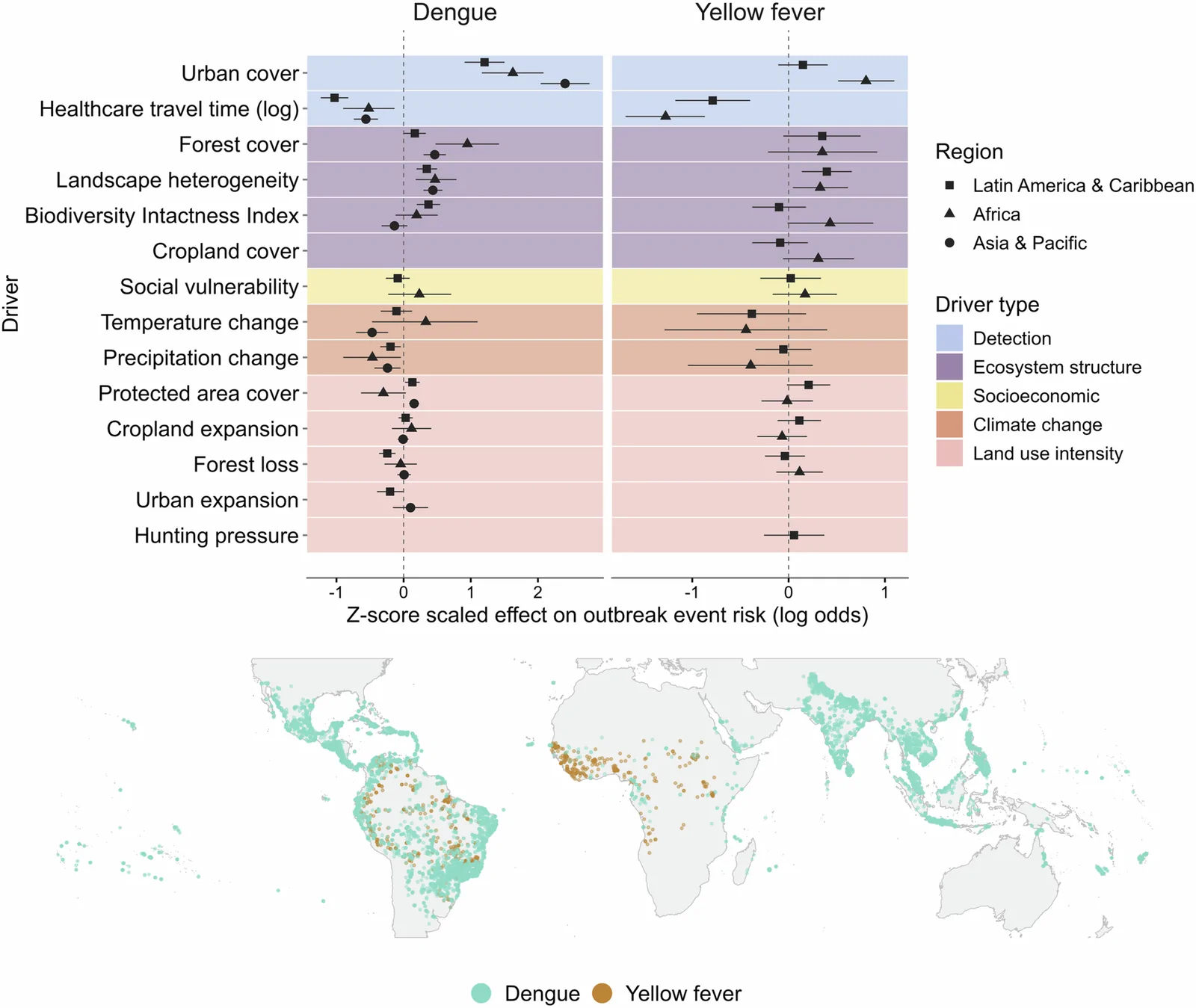
Region-specific variation in the inferred drivers of dengue and yellow fever. Points and error bars show linear fixed effect estimates (posterior mean and 95% credible interval) for geospatial logistic regression models fitted separately for different global subregions for dengue (Latin America, n = 3,782; Africa, n = 2,271; Asia & Pacific, n = 10,062) and yellow fever (Latin America, n = 1,178; Africa, n = 2,344). Point shape denotes region, and background colour denotes driver type. The map shows the distribution of outbreak event records for dengue (green) and yellow fever (brown).

Our models also detected numerous well-known or strongly suspected drivers of specific diseases, further validating the approach: these included higher biodiversity intactness reducing Lyme disease risk (consistent with foundational disease ecology research into the dilution effect^7,28^); pig density and climate change trends as drivers of Japanese encephalitis^39^; a consistent fingerprint of long-term precipitation drying on dengue outbreak risk across the Americas, Africa and Asia^34^ (Extended Data Fig. 8); an increased risk of avian influenza A/H5N1 outbreaks in areas with higher poultry density^40^; forest loss increasing risk of mpox and zoonotic malaria^41^ and evidence of fragmented forest cover driving outbreaks of arboviruses that emerge at human–forest ecotones^42^ (Mayaro fever, Oropouche fever and yellow fever) (Extended Data Figs. 6 and 8). Finally, for Argentine haemorrhagic fever, after adjusting for detection processes, we found that outbreak events are more frequent in relatively more socially vulnerable areas, which aligns with the disease’s rodent-borne transmission ecology^43^. Surveillance data on this disease were the most consistently and precisely geolocated in our entire database (Extended Data Table 1), demonstrating the value of precise, standardized spatial surveillance reports for reducing the confounding impacts of detection bias.

## Shared drivers differ by pathogen transmission mode

**Fig. 4:**
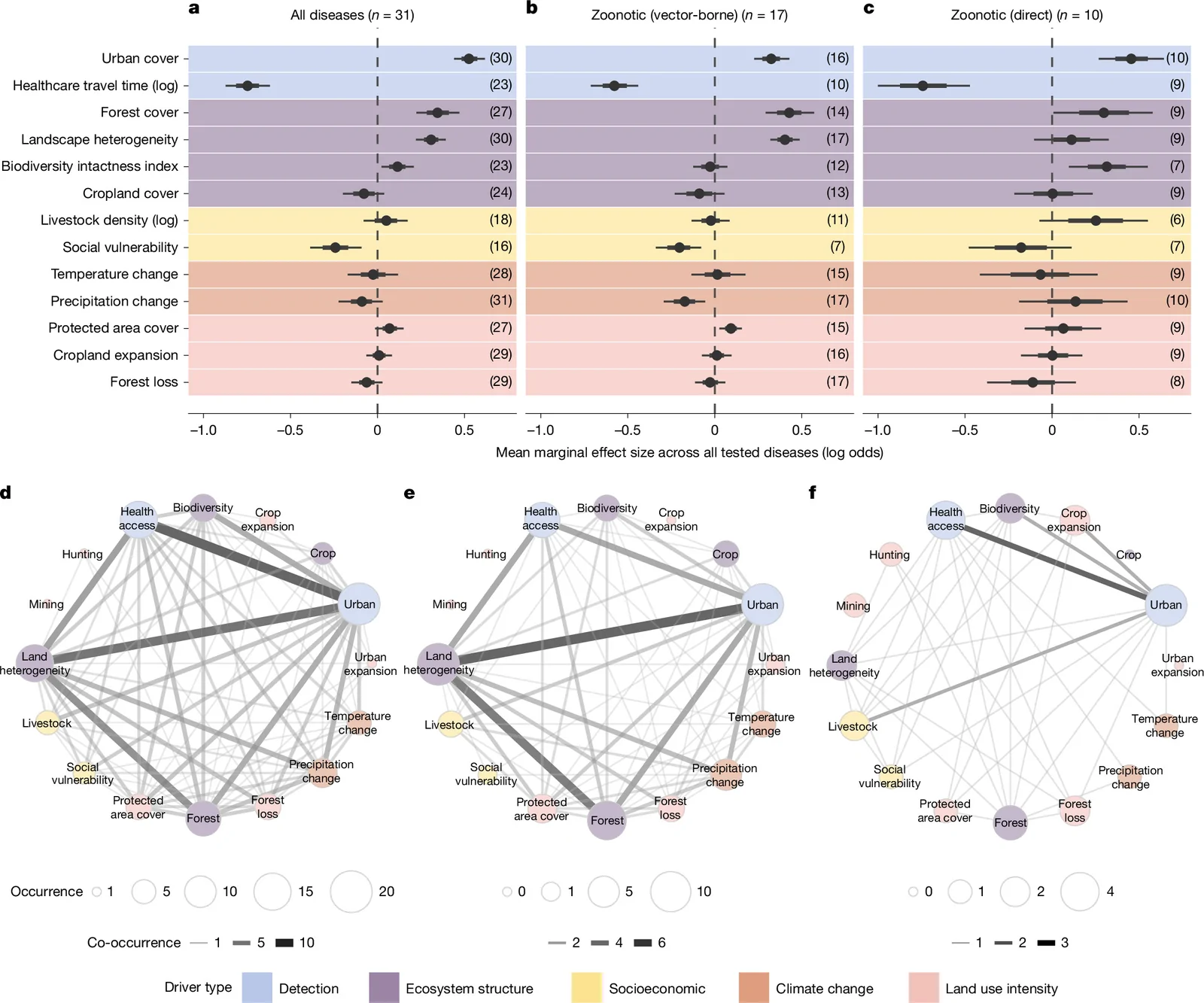
Mean effect sizes and shared outbreak drivers differ between emerging infectious disease groups. **a**–**c**, Results are summarized across individual disease models, for all diseases (**a**; *n* = 31) and for subsets of zoonotic diseases whose main mode of transmission to humans is either vector-borne (**b**; *n* = 17) or direct (**c**; *n* = 10). For each driver, estimates of the mean marginal effect size are shown (posterior median, 67% and 95% credible interval) across all diseases for which that driver was tested. Mean effect distributions were generated using 5,000 sets of posterior samples drawn from individual fitted disease models (Methods). Numbers in parentheses show the number of diseases used to calculate the mean effect; only drivers tested for at least five diseases were included (hunting pressure, mining and urban expansion were excluded). **d**–**f**, Unipartite networks show the patterns of driver sharing between all diseases (**d**; *n *= 31), vector-borne zoonoses (**e**; *n *= 17) and directly transmitted zoonoses (**f**; *n *= 10). Nodes represent drivers, with node size proportional to the number of diseases with strong evidence of a non-zero effect (‘driver occurrence’), and edges are weighted by the number of diseases for which driver pairs co-occur (‘driver co-occurrence’; that is, non-zero inferred effects of both drivers in multivariable models). Node colours denote driver type. Larger nodes reflect more prevalent drivers and darker edges reflect a higher rate of driver sharing within each group of diseases. Results are shown for ‘majority rule’ hypothesis-led models; results using other criteria are very similar (Supplementary Figs. 2 and 3). Source data

Prevailing narratives about disease emergence tend to focus on the impacts and relative importance of individual drivers, but outbreak risks typically arise from several social or environmental processes acting synergistically^6^. For pathogens that share similar ecological characteristics, convergent responses to certain drivers—for example, similar host or vector community responses to land use pressures^20,22^—might also cause multiple infections to cluster within the same population, potentially leading to worse outcomes (that is, syndemic interactions)^44,45^. To explore the evidence for shared drivers among pathogens with similar transmission modes, we estimated mean driver effect sizes and patterns of driver co-occurrence, across all 31 diseases and separately for either directly transmitted (*n* = 10) or vector-borne (*n* = 17) zoonoses (Fig. 4 and Supplementary Figs. 2 and 3). For each driver, we generated a posterior distribution of the mean effect size across all tested diseases, which incorporates the variation in uncertainty between diseases (arising from differences in sample size and variability; Fig. 4a–c). To examine shared drivers, we visualized driver occurrence and co-occurrence between diseases as unipartite networks (Fig. 4d–f). Across all diseases, there were large mean increases in outbreak risk with higher levels of landscape fragmentation and forest cover, weaker increases in risk with higher biodiversity intactness and moderate decreases in risk in more socially vulnerable settings (possibly reflecting residual detection bias) (Fig. 4a). Certain drivers co-occur frequently overall, principally urban cover and healthcare access, and, to a lesser extent, fragmented vegetation and forest cover (Fig. 4d). This pattern does not simply reflect landscape structure; for example, forest cover and landscape heterogeneity are uncorrelated globally, whereas cities, travel time to healthcare and landscape heterogeneity are, at most, moderately correlated (Extended Data Fig. 4).

Notably, the magnitude and co-occurrence of driver effects differ substantially by transmission route. Zoonotic diseases that are transmitted to humans by arthropod vectors (for example, Chagas disease, Lyme disease and yellow fever) are more strongly and consistently sensitive to ecosystem drivers and long-term climate drying, with large mean effect sizes (Fig. 4b) and a much higher rate of driver sharing between diseases (Fig. 4e). These findings reinforce the importance of ecotonal, fragmented and urbanizing landscapes as critical nexus points for vector-borne disease outbreaks^16,46–48^. Ecosystem-based strategies, such as protecting intact forests or regulating the financial actors most responsible for extractive land use^49^, may therefore have greater potential to mitigate the burden of these diseases.

By contrast, for directly transmitted zoonoses (for example, Ebola virus disease, Middle East respiratory syndrome (MERS) and mpox) we found less evidence of consistent directional effects, and very little evidence of sharing of drivers (Fig. 4c,f). This may be due partly to the low number of documented outbreak events for several of these pathogens, which increases inferential uncertainty (Figs. 1c and 4c), but it also probably reflects their diverse ecologies, life cycles and human exposure pathways (for example, hunting, contact with livestock and food products, household contact with wildlife^50^ or occupational contact with wildlife, such as through agriculture^51^). These findings do not support the idea that one-size-fits-all ecological interventions (for example, tighter global regulations on deforestation and agricultural expansion) would be broadly protective against epidemic and pandemic threats, such as directly transmitted respiratory and haemorrhagic fever viruses. Ecosystem-based surveillance and risk prevention programmes remain among the most scientifically supported options to reduce spillover risk and improve outbreak detection at human–animal interfaces^9^, but our findings suggest that such interventions should be tailored to the ecology of specific priority pathogens in specific landscapes. In systems where this evidence is currently limited, long-term ecological research can establish these principles in striking detail^6^.

## Healthcare access supports detection and response

Even with our extensive dataset of 32 diseases, representing a wide variety of different pathogens, biomes and socioeconomic contexts, our study remains limited by sample size and data quality. Detection and reporting biases are a pervasive, worldwide phenomenon, cutting across spatial scales and low- to high-income settings (Figs. 2 and 3). Strikingly, many of the highest-concern pathogens, such as bat-borne epidemic viruses, have the lowest availability of data (Fig. 1). These gaps probably reflect under-detection rather than a true scarcity of spillover events. Previous studies have estimated that up to half of all Ebola outbreaks might never have been identified^52^—a pattern also evident in serological surveys for many high-concern zoonotic pathogens (for example, severe acute respiratory syndrome (SARS)-related bat coronaviruses^53^ and Lassa fever^54^). These results highlight an underappreciated and disease-agnostic lever for intervention: improving access to healthcare in underserved rural and remote communities. In much of the world, it can take over a day to reach a healthcare facility, especially without motorized transportation^55^, and remote clinics often lack capacity for molecular diagnostics, especially for rare infections; these gaps in health systems are likely to be persistent at high-risk interfaces between rural communities and intact ecosystems. Outbreaks that start further from health centres are less likely to be detected, promptly diagnosed and treated and, without a timely response, may be more likely to grow into epidemics^56^.

**Fig. 5:**
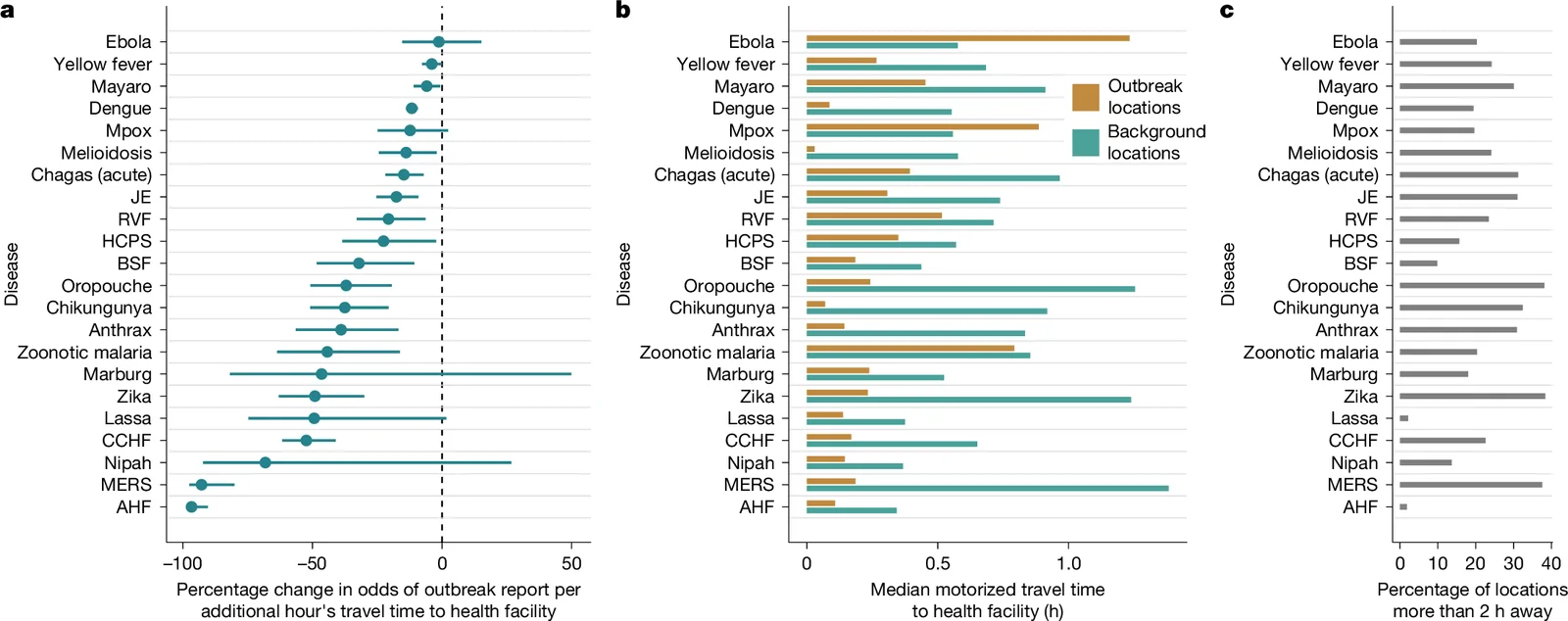
Outbreak detection declines with increasing distance from healthcare facilities. **a**, Points and error segments show the marginal percentage change in the odds of outbreak reporting for each additional hour of motorized travel time from the nearest health facility (posterior mean and 95% credible interval from each of 22 fitted disease models). **b**, Barplot showing, for each disease, the median motorized travel time to the nearest health facility across outbreak locations (brown) compared with population-weighted background locations (a representative background sample across the at-risk area; green). **c**, Barplot showing the percentage of population-weighted background locations falling more than 2 h from the nearest health centre. This covariate effect was tested for 23 diseases (SLE was not visualized owing to extremely wide uncertainty intervals) but not for the remaining 8 diseases (mostly in the USA) owing to high collinearity with urban cover (Methods). Source data

For most of the diseases we examined, outbreak event reports cluster near clinics: outbreak odds declined by a median of 32% (range 1.2–96.7%) for each additional hour’s motorized travel time from the nearest healthcare facility (Fig. 5a). Average travel times to healthcare across each disease’s entire geographic range are generally much higher than at documented outbreak locations, with a substantial proportion of population-weighted background locations falling over 2 h away (median 23%, range 2–38%; Fig. 5b,c). These model-derived travel times probably represent best-case estimates, given socioeconomic disparities in access to motorized transport, the tendency for models to underestimate actual travel times (for example, owing to road quality or traffic)^57^, and the many additional non-geographic barriers to accessing healthcare. Investing in new infrastructure and lowering social and economic barriers to access would ensure timely disease diagnosis, treatment and prevention for underserved communities, and so substantially increase the odds of outbreak detection and reporting. Improving global surveillance of infectious diseases at human-nature interfaces—for example, paired syndromic and serological surveillance across networks of sentinel sites to track spillover and disease rates in people and animals^58^—would also help address the data gaps highlighted by this study, and therefore help strengthen scientific evidence around ecological strategies for risk reduction.

## Conclusion

The recent rise in emerging infectious diseases is often described as a byproduct of global anthropogenic environmental change. This trend may share common causes with both the climate and biodiversity crisis, but is also a ‘wicked problem’^59^ in its own right: our findings suggest that emerging infectious disease risks are ubiquitous, and associated widely with mosaic landscapes where people and cities live alongside forests and fragmented ecosystems. Our approach has expanded substantially on earlier global studies by examining comprehensive outbreak geographies (rather than solely points of first emergence), testing disease-specific socio-ecological hypotheses, and adjusting for data biases at several scales. This has provided a more nuanced picture of how anthropogenic impacts vary across the diversity of emerging human infections. We find evidence of a strong and consistent anthropogenic fingerprint on vector-borne diseases, suggesting that over the coming decades, risk is likely to increase unless there are substantial shifts in human activities. At the local scale, ecosystem-, awareness- and health system-based interventions could reduce the burden of vector-borne diseases with immediate effect; over longer timescales, efforts to mitigate climate change will help slow the expanding burden of many mosquito- and tick-borne diseases, with the greatest impacts felt over the coming decades^16^. By contrast, we were unable to detect a similarly consistent fingerprint on directly transmitted zoonotic infections, diverging from popular emergence narratives that have been based largely on evidence from in-depth case studies^26^. In any given region, investments in ecological and community-led research are needed to identify and evaluate locally tailored ecosystem interventions to reduce spillover risk and the endemic burden of regional priority diseases. Meanwhile, as new diseases continue to emerge, and human activities continue to transform the planet even in the best-case scenarios for future development, we suggest that the global community should redouble their investments in health system strengthening. Achieving universal health coverage, strengthening outbreak response capacity, and investing in new vaccines and therapeutics, can help to ensure that outbreaks do not have the opportunity to become epidemics and pandemics.

## Methods

The aim of this study was to test empirically for a general detectable fingerprint of anthropogenic drivers on the geographical distribution of human outbreaks of 32 emerging infectious diseases, based on existing geolocated outbreak and case data sources, and gridded datasets representing key socio-environmental disease drivers. To account for differing ecological characteristics across diseases and avoid testing for spurious or irrelevant associations, we generated a set of hypothesized key drivers for each individual disease through a participatory form-based exercise completed by most co-authors, whose disease-specific expertise spans a wide range of disciplines and scales of enquiry (from virology, to ecology, to global public health). Across all diseases overall, and individually per disease, we applied a standardized statistical inference framework, which involved harmonization of point and polygon data and inference of the drivers of outbreak risk using geospatial logistic regression models. We describe these methodological stages in detail in the following sections.

### Collection and harmonization of geolocated human disease data

We collated and harmonized geolocated point and polygon data on human case occurrence and/or incidence of 32 environmentally linked emerging infectious diseases, from numerous published datasets in the scientific literature and from open national disease surveillance data portals (Fig. 1, Extended Data Table 1 and Supplementary Table 1). We used the following broad criteria to select diseases for inclusion: (1) human infection risk should be coupled closely and thus, in principle, attributable to local environmental or ecological conditions (that is, transmission should be zoonotic, vector-borne or environmentally mediated), and if extended human-to-human transmission chains independent of these conditions are possible, datasets must specify the locations of probable index cases. (2) Diseases should not be sufficiently well-surveyed that prevalence surveys, rather than case incidence or occurrence, could form the basis for inference. (3) Diseases should not have been subject to long-term eradication programmes that could confound inference of environmental drivers. These criteria meant that our analyses included many emerging, rare and high-concern zoonotic and vector-borne pathogens (including many mosquito-borne arboviruses, rodent- and bat-borne viruses and *Plasmodium knowlesi* zoonotic malaria), but not *Plasmodium falciparum* or *Plasmodium vivax* malarias or neglected tropical helminthiases.

The full list of diseases, data sources and their spatial and temporal coverage is provided in Extended Data Table 1. When compiling data for each disease, our priority was to select datasets that covered as much of the known geographic extent of transmission as possible, while remaining internally consistent (that is, collated in a standardized and comparable way to facilitate analysis). We focused on compiling existing published datasets from scientific literature and openly accessible disease surveillance portals, rather than collecting additional data (for example, by scraping scientific literature or ProMED), to ensure that our analyses are representative of data that are currently in the public domain and relatively analysis ready. Notably, sufficient or suitable data were not available for certain high-priority diseases, most notably SARS-related coronaviruses, because too few confirmed spillover events have been documented to provide a geographic picture of risk^60^. Datasets were obtained either by downloading from scientific paper supplementary data or open repositories, sharing between study co-authors, or through email requests to specific paper lead authors. To credit the substantial work involved in compiling the source datasets and ensure our author team included disease-specific expertise, lead authors who collated and shared datasets were invited to be study co-authors and participate in hypothesis generation (see ‘Disease-specific hypotheses for the drivers of human infection risk’) and manuscript writing and editing (see ‘Author contributions’ for a breakdown of roles).

Human case datasets are generally available in one of two formats. (1) Geolocated spillover or outbreak occurrences. Here, records represent one or more cases occurring at a named place and time, with geographical precision ranging from a specific point location or point with buffer radius (more precise), to a named administrative unit (less precise). This category of data includes most of the datasets collated for the purpose of risk mapping^61–76^. (2) Case counts from named areal units. Here, records contain the number of cases reported from a particular areal unit (usually first or second-level subnational administrative divisions) during a particular time window (usually a month or year). This category includes mainly datasets collected and reported through national notifiable disease surveillance systems, which are often available through online portals, reports or scientific papers. Point locations can provide greater geographic precision on environmental conditions nearby to a reported disease case, whereas administrative units require averaging conditions across often much larger polygons. Consequently, different sources provide different levels of information about both transmission intensity (binary outbreak occurrence versus number of cases) and environmental context (specific event location versus broad aggregated unit).

To ensure that the results of our models were comparable across diseases and datasets (Extended Data Table 1), we developed a harmonization framework to accommodate these diverse data sources while preserving spatial uncertainty in location of infection. A diagram of this pipeline is shown in Extended Data Fig. 1 and described as follows. The response variable, an ‘outbreak event’, was defined as at least one case in a named locality in a specified year, to ensure comparability in analyses between geolocated outbreak datasets (which contain no or partial information about the number of cases) and surveillance data (which typically provide an estimate of disease incidence). For any given disease, all outbreak locations (whether originally point or polygon) were converted to polygon objects using the ‘sf’ package v.1.0.23 in R^77^, by drawing a circular buffer around point locations with a radius of either 5 km, or another custom value if specified within the source dataset. This buffer size was chosen to retain a relatively high degree of spatial precision for linking point locations to environmental covariates, and assumes that infection events occurred near to where they were reported and geolocated (see ‘Limitations of data and methodology’). All polygons covering too large a spatial area were excluded as too imprecise to link to local environmental conditions; this was by default greater than 5,000 km^2^ (equivalent in area to a circular buffer with a radius of 40 km) but was relaxed to higher values (mostly under 10,000 km^2^, but maximum 20,000 km^2^) for certain data-sparse diseases and coarser areal case surveillance datasets (Brazilian spotted fever, chikungunya, Eastern equine encephalitis, influenza (H5N1), JCE, Marburg virus disease, Mayaro fever, Oropouche fever, plague, RVF, SLE, West Nile fever and yellow fever), as a compromise to retain as complete a geographical picture of outbreak event distributions as possible.

For each disease, this process produced a dataframe where each row with a unique identifier represents an outbreak event (that is, 1 or more cases in a given locality in a given year) with metadata where available (number of cases, case definition, diagnostic method, etc), along with an associated shapefile linking each record to a geographical polygon. For most diseases the shapefile contained a mixture of smaller circular buffers around point locations (with radius between 5 and 20 km) and larger, irregularly shaped administrative unit polygons. This variation in spatial uncertainty associated with infection events means that environmental covariates were necessarily averaged across varying geographic areas (see below), which is a feature inherent to most analyses of aggregated disease surveillance data^78^. We found that mean covariate values were correlated highly across a representative range of buffer sizes (Pearson’s rho > 0.8), suggesting that this issue is unlikely to substantially impact our model results (Supplementary Fig. 4).

Across all diseases, the full database contained 58,319 unique georeferenced outbreak events, for 32 diseases, in 169 countries worldwide (Fig. 1). Most records (88.7%) were from after 2000 and very few records (2.4%) were from before 1980. Most outbreak events were associated originally with spatial polygon data (73.1%), and the remaining 26.9% with point locations. The constraints of available data mean that these datasets are necessarily presence-only (that is, contain only information on positive case detections without true negatives as controls), so later modelling analysis required the selection of background points as pseudo-controls (Extended Data Figs. 1 and 2; see ‘Statistical modelling’).

### Disease-specific hypotheses for the drivers of human infection risk

Many studies have proposed that certain anthropogenic changes may act as shared drivers of risk across numerous zoonotic, vector-borne and environmentally mediated diseases (for example, agriculture and urban expansion, deforestation, wildlife hunting, biodiversity loss). Yet, given their wide diversity of reservoir hosts and transmission ecologies, disease drivers may often be pathogen- or context-specific. We therefore identified sets of hypothesized drivers to test for each individual disease, to ensure our analyses accounted for expected ecological differences between systems, and to avoid identifying spurious or implausible drivers for any given system (for example, West Nile disease and wildlife hunting). Such an issue could otherwise feasibly arise owing to the small size and spatially biased nature of many disease datasets (see ‘Limitations of data and methodology’).

First, we developed a list of 18 specific socio-ecological factors that are considered principal proximal drivers of emerging disease outbreaks, including social/socioeconomic factors, human–animal contact interfaces including food systems, landscape structure, anthropogenic stressors on ecosystems and climate change. This list was developed by the lead author team who have extensive expertise in zoonotic, vector-borne and emerging diseases (R.G., S.J.R. and C.J.C.) and aimed to include as diverse and representative as possible a set of drivers, while excluding those whose influence is probably too dynamic to be detectable within spatial outbreak data (for example, extreme climate events and wildfires). The final list included ecological and landscape factors (forest and cropland cover, landscape fragmentation, biodiversity loss, invasive species), anthropogenic land use intensity and human–wildlife contact (cropland expansion, mining, protected area coverage, urban cover and expansion, wildlife hunting, wildlife trade and markets), climate change (long-term changes in temperature and precipitation), and social processes that influence exposure and/or detection (socioeconomic vulnerability, proximity to hospitals/clinics, livestock density).

Next, we identified key hypothesized drivers to test for each disease system, using a participatory exercise that was completed by study co-authors. This was conducted as a team-wide exercise, both to take advantage of the diversity of disease-specific expertise across the study authorship, and to avoid the potential for biases if hypotheses were developed by only one or a few analysts. Owing to the geographically dispersed nature of the author team, this was designed as an online form with detailed instructions. The form was structured as a fill-in matrix spreadsheet of 18 drivers and 34 disease systems (the form and instructions are provided in Supplementary Table 2). Each cell could be filled in by each author indicating their choice (four choices) of a driver having a negative, positive, none or ‘don’t know’ impact, and authors were also asked to provide a ranking (1–3) for their expected top three drivers for each disease (in either direction). Because authors have research backgrounds in different diseases, they could opt out of completing the exercise for any disease system (by leaving these blank; NA), to ensure they only selected hypotheses for diseases for which they considered they had sufficient expertise (Extended Data Fig. 5). The form was developed by two lead authors (S.J.R. and C.J.C.) and tested independently by a co-author that was not involved in the form design (C.A.L.) to ensure instructions were clear and unambiguous. This exercise was completed independently by 25 of the 31 study authors (Extended Data Fig. 5), with 6 non-participants either because they were unavailable when the exercise was conducted (3), joined the authorship after the exercise was conducted (1) or do not have infectious disease-specific expertise and instead contributed to covariate design (2). None of the authors had seen the combined dataset or preliminary results before completing the exercise, except the lead analysts (R.G., C.J.C.) who had seen preliminary results for three diseases during pipeline development (Ebola, Lassa, CCHF) but not the results of fully adjusted models with geospatial and detection effects.

The authorship includes members with significant expertise and disease-specific publications for most of the infections included in this study, including anthrax, rodent-borne arena- and hantaviruses, Chagas disease, urban *Aedes*-borne arboviruses, zoonotic mosquito-borne arboviruses, CCHF and other tick-borne infections, avian influenza, bat-borne henipaviruses and filoviruses, Lyme disease, melioidosis, MERS, mpox and related orthopoxviruses, and plague. The group has a diverse set of disciplinary backgrounds, including disease ecology and evolution, epidemiology, microbiology and virology, genomics, veterinary medicine, social-ecological systems and public health. Nonetheless, our team still consists largely of academic researchers based in Global North institutions, and as such our hypotheses are unlikely to fully reflect locally situated understandings of most of these diseases. Although this online exercise was a concise approach to participatory hypothesis development across an international author team, authors still reported spending several hours (more than 2 h) to fully complete the matrix, so we cannot recommend this current design as the basis for a large-scale survey.

We then postprocessed the completed exercise data to generate lists of hypothesized drivers to test for each disease, based on three different levels of stringency for author agreement, as a sensitivity test to ensure our approach did not introduce systematic bias in the findings. We first adopted a moderate stringency criterion, including drivers for which more authors stated an effect (either positive or negative) than stated no effect (‘majority rule’); this retained 80% of disease–driver combinations to test. We also adopted a high-stringency criterion, including only drivers that were included in the top three ranked drivers by at least one author (‘top-ranked’; retaining 50% of disease–driver combinations), and a lowest stringency criterion, including all drivers for which at least one author had stated an effect (‘any author’; retaining 97% of disease–driver combinations). The ‘any author’ criterion tests nearly all potential relationships, excluding only extremely implausible drivers, so under this criterion the results are driven primarily by the patterns within the data, with minimal influence of the hypothesis exercise. The study results were quantitatively and qualitatively very similar under these three criteria, indicating that our findings were not biased systematically by the hypothesis exercise. We present results using the ‘majority rule’ criterion in the main figures, as this criterion reflects the balance of author agreement, and present results for the other criteria as Supplementary Information (Supplementary Figs. 2 and 3).

### Collation of geospatial data on socio-environmental drivers of disease

In parallel, we collated global geospatial (raster) layers describing socio-environmental and climatic features as proxies for the key geographic drivers of risk listed above, based on remote sensing, climate reanalysis, social indicators and census-based data sources. A full table of socio-environmental covariates, their sources and processing is provided in Extended Data Table 2 and Supplementary Table 3. The small size of many disease datasets unfortunately meant there was insufficient data to analyse the relationship between cases and covariates in both space and time, which therefore limited our study to spatial rather than spatiotemporal driver analysis (see ‘Limitations of data and methodology’). Therefore, for variables describing gross characteristics of the environment (for example, land cover type proportion variables) we selected a single raster year or time period close to the central tendency of reported disease data (between 2005 and 2015), while aiming for gridded products that provide the best spatial and thematic resolution possible under that constraint. For variables describing anthropogenic change, we generated rasters that described the grid-cell-level change in a particular variable across most of the disease data period (for example, tree cover loss between 2000 and 2020, change in mean annual temperature between 1950–1970 and 2000–2020). Raster covariates were used at their original spatial resolution with a few exceptions (for example, social vulnerability was aggregated; Extended Data Table 2); as this was not a mapping study, no rescaling was required. In all cases a key criterion was, wherever possible, to use global raster products that were comparable across regions and diseases. For most drivers there was a natural match to proxy covariate metrics (for example, percentage land cover variables, average climatology change from a historical reference, livestock density, cumulative tree cover loss, cropland or urban expansion); we selected ERA5-Land for climate owing to its rich historical coverage and high accuracy, and Copernicus land cover for land cover variables owing to its combination of high spatial and thematic resolution. For other drivers, we selected the only available globally comparable spatial product (Biodiversity Intactness Index; Global Relative Deprivation Index). Certain drivers involved choosing a specific product over other options: we selected the modelled hunting pressure index for tropical forests^79^ owing to its clearly reported modelling approach and its geographic extent, which was larger than that of other hunting pressure maps; and we selected EVI dissimilarity from ref. ^80^ as a landscape heterogeneity metric, because the original study showed this metric was sensitive to anthropogenic fragmentation and correlated strongly to biodiversity metrics (so a suitable proxy for the ecological dynamics of fragmented landscapes). Notably, we were unable to identify suitable proxy covariates for several widely hypothesized drivers that have not been quantified in space and time, highlighting an important lack of systematic spatial data collection around key putative drivers of disease emergence; these include invasive species density, wildlife trade and/or live markets, and wildlife hunting outside tropical forests.

A brief description of the full list of the socio-environmental raster datasets is as follows: temperature change (change in grid-cell-level mean annual air temperature between reference period of 1950–1970 and focal period of 2000–2020, derived from ERA5-Land reanalysis^81^); precipitation change (change in grid-cell-level mean annual precipitation between 1950–1970 and 2000–2020, from ERA5-Land); forest cover (grid-cell-level fractional tree cover from Copernicus land cover 2015); forest loss (grid-cell-level tree cover loss 2000–2020 from Global Forest Change); biodiversity intactness (local Biodiversity Intactness Index, that is, the predicted average local abundance of all species relative to their abundance in minimally disturbed habitat, for 2005 based on human disturbance layers^82^); cropland cover (grid-cell-level fractional crop cover from Copernicus land cover 2015); cropland expansion (grid-cell-level cropland growth 2000–2019^83^); landscape heterogeneity (grid-cell-level second-order EVI dissimilarity index 2005, a metric of landscape fragmentation sensitive to anthropogenic landscapes^80^); hunting pressure index (a modelled defaunation index measuring average hunting-related species declines in tropical forest biomes^79^); protected area cover (whether grid cell is under area-based conservation, based on the World Database of Protected Areas 2022); mining cover (whether grid cell is under mining land use, based on ref. ^84^); social vulnerability (the Global Gridded Relative Deprivation Index, a composite indicator based on inputs including infrastructure, human development index, nighttime lights and infant mortality rate, for a nominal present-day period^85^); travel time to healthcare (road-based travel time to nearest hospital or clinic for nominal year 2015^55^); urban cover (grid-cell-level fractional urban cover from Copernicus land cover 2015); urban expansion (grid-cell-level expansion of built-up areas 2000–2019 derived from ESA-CCI land cover); and livestock density (grid-cell-level density of livestock types from Gridded Livestock of the World v.3).

### Statistical modelling

To infer the drivers of the geographic distribution of human cases while accounting for spatial and detection biases, we applied a standardized geospatial modelling approach for each disease.

For each model, we first defined the geographical boundaries of the modelling area (‘study region’). For datasets compiled from the scientific literature, this was defined as a smoothed convex hull polygon around the full extent of geographical case occurrences, with a buffer of 180 km (Extended Data Figs. 1 and 2). For national-level case surveillance data the study area was constrained to the borders of the relevant country or subnational region. We then generated a final case-control dataset for modelling. We excluded records from before 1985 for most diseases, to better align the disease data with the timescale of available covariates; exceptions were certain data-sparse diseases where data from after 1980 were included, to retain as much information as possible (anthrax, Ebola virus disease, Marburg virus disease, Mayaro fever and Oropouche fever).

Because the case data were presence-only, meaning there were no true negative controls, we then generated background (pseudo-control) points throughout the study region. We selected between two and eight times as many background points as presence points, with the aim of ensuring sufficient coverage of the background area while balancing against computational costs. The higher numbers were selected for diseases that had a low number of outbreak points across a large study area (for example, Oropouche, Ebola) to ensure coverage of the full study area, whereas the lower numbers were selected for diseases with a large number of outbreak points across the study area (for example, Lyme, West Nile). (Various guidelines have been proposed for background points selection to maximize predictive performance in species distribution models^86^, but our study objective is inference of driver effects rather than optimizing for prediction; as such, our priority is statistical power and coverage of the study region for each disease). All else being equal, the null expectation is that the distribution of human disease cases would follow the distribution of population; as such, entirely spatially random selection of background points would over-represent sparsely populated rural areas and under-represent highly populated urban areas. Therefore, we weighted background points distribution by human population, that is, randomly generated point locations with the probability of a location being selected proportional to log+1-transformed population. This was based on a global raster of 2010 human population per pixel (WorldPop’s top-down unconstrained mosaics^87^), at 1-km resolution for most diseases, but 10-km resolution for certain diseases spanning a multi-continent geographic range to limit computation time (for example, dengue, chikungunya). This approach produced a pseudo case-control design, that is, comparing the socio-environmental conditions experienced by human populations at the locations where outbreaks have occurred (cases) with a representative background sample of the conditions experienced by populations across the study region (‘controls’). Circular buffers were created around each background point with an area equal to the median area of the outbreak location polygons, to ensure covariates were averaged across a comparable geographical area for both presence and background points (Extended Data Fig. 1).

For each model, this process produced a final dataframe of presences and pseudo-absences with associated polygons (again using ‘sf’), from which we extracted the mean value for each raster covariate using the package ‘exactextractr’ v.0.10.1. We excluded from the analyses any variables that were missing data for greater than 10% of observations or contained zeroes for greater than 95% of observations. We examined collinearity among covariates using visual inspection, correlation matrix plots and variance inflation factors, and identified and excluded highly collinear covariates from multivariable models; this step was conducted manually rather than programmatically, to prioritize the inclusion of covariates with a stronger hypothesized relationship to each disease in question (based on the ‘majority rule’ hypothesis list). The final sets of covariates included in each disease-specific multivariable model are visualized in Extended Data Fig. 6.

To infer relationships between covariates and disease outbreak probability *P* at location *i* (log odds of occurrence), we fitted geospatial logistic regression models in a Bayesian inference framework (integrated nested Laplace approximation, implemented in the package ‘INLA’ v.23.3.26 (refs. ^88,89^)), with the following general formula:

$$\begin{array}{c}{y}_{i} \sim {\rm{B}}{\rm{e}}{\rm{r}}{\rm{n}}({p}_{i})\\ {\rm{l}}{\rm{o}}{\rm{g}}{\rm{i}}{\rm{t}}({p}_{i})=\alpha +{\rho }_{i}+\sum _{j}{{\boldsymbol{\beta }}}_{j}{X}_{j,i}\end{array}$$

Here, α is the intercept, ${\rho }_{i}$ is a continuous spatially structured random effect and **β** is a vector of linear fixed effects parameter estimates for the matrix of j covariates ${X}_{j}$. The geospatial effect was specified as a Gauss–Markov random field fitted using a stochastic partial differential equations approach, with penalized complexity priors on the range and sigma parameters and an intermediate mesh density chosen to balance reasonably between spatial precision and computation time. We set Gaussian priors for intercept and linear fixed effects (mean = 0, precision = 1). Different diseases varied widely in both geographic range size and patchiness of data, so to avoid issues with overfitting or underfitting of the spatial field, for each disease we manually adjusted the hyperpriors for the stochastic partial differential equation model’s Matern covariance function (range and variance hyperparameters) to ensure that the spatial field was fitted smoothly to the spatial structure of the outbreak event data (that is, there were no visible issues with inference of the geospatial effect; Extended Data Fig. 2). After model fitting, we then extracted the Watanabe–Akaike information criterion as a comparison metric of model fit.

#### Global multi-disease models

We first fitted general models to infer drivers of risk for all disease outbreaks—not differentiating between diseases—using the full dataset of 49,239 outbreak events (after excluding early and spatially imprecise records) and 50,000 population-weighted background points across the global study area. The extreme imbalance in sample sizes between different diseases (Fig. 1) could lead to estimates being biased by well-represented diseases. To avoid this issue, we estimated fixed effects posterior distributions for each driver from an ensemble of 100 submodels fitted to balanced subsamples of the data. Each submodel was fitted to a dataset including 100 randomly subsampled outbreak points per disease (all outbreak points for any diseases with fewer than 100 points in total), and twice as many randomly subsampled background points. We drew 1,000 posterior samples per fixed effect from each submodel, then calculated credible intervals (median, 67% and 95% intervals) using pooled posterior samples from all 100 submodels. This approach ensured a balanced representation of data across all diseases when inferring overall driver effects. We applied this ensemble approach for each of the models described in the following paragraph, but for simplicity we refer to the pooled results from each ensemble as a ‘model’.

To examine the potential confounding effects of local detection processes and broad-scale patterns of reporting effort on inferred drivers, we fitted three global models: (1) including only socio-environmental covariates, that is, with no outbreak detection-specific covariates (urban cover and healthcare travel time) and no geospatial effect; (2) adding a geospatial effect but no local outbreak detection-specific covariates; and (3) a full model with outbreak detection covariates and a geospatial effect (Fig. 2). For comparison and sensitivity checking by transmission pathway, we also fitted the full geospatial and detection covariate model for subsets of pathogens defined as either zoonotic (principally transmitted to humans from an animal reservoir; *n* = 26) or vector-borne (transmitted to humans by arthropod vectors irrespective of host, that is, also including anthroponotic arboviruses such as dengue fever; *n* = 20), whose risk is expected to be coupled tightly to local ecosystem characteristics (Extended Data Fig. 3a). Given the significant regional variability in the intensity and correlation structure of emerging disease drivers (Extended Data Fig. 4), we also fitted five models to data from different geographic regions (North America, Latin America and the Caribbean, Sub-Saharan Africa, South Asia and East Asia and the Pacific), to examine the consistency of inferred drivers across these varied socio-ecological contexts (Extended Data Fig. 3b).

#### Individual disease-specific models

We fitted individual models for all diseases except Hendra virus disease, for which the number of human outbreak points was too low for reliable model fitting (Extended Data Table 1). The process of inferring drivers for each individual disease (*n* = 31) was as follows. First, we fitted separate geospatial models which included each covariate individually (‘univariable’) plus a geospatial random effect to account for the broad geographical pattern in outbreak occurrence, but not possible finer-scale confounding by other variables (particularly detection proxies). We then fitted three separate hypothesis-driven multivariable geospatial models including drivers identified using the three filtering criteria from the co-author exercise (majority rule, top-ranked and any author) (Extended Data Fig. 5). Because of the strong a priori expectation of detection bias driven by health systems proximity and accessibility, all multivariable models included both travel time to healthcare and urban cover; except in instances where these were highly collinear with each other; in these cases, the driver identified as most important in the hypothesis-generation exercise was selected. For all models where forest loss, cropland expansion or urban expansion were hypothesized as drivers, we also included either forest cover, cropland cover or urban cover, respectively, to account for the inherently spatially correlated process of land use change. For most diseases, urban expansion and urban cover were highly collinear at the scale of this analysis (Pearson’s *P* > 0.8) so urban expansion was almost always excluded from multivariable models. For diseases with strongly hypothesized associations to specific livestock, the livestock covariate was based on gridded data for only the most relevant livestock type(s) (for example, poultry for influenza, ruminants for RVF; Supplementary Table 1 and Methods); the exception was MERS, as gridded camel density data are not openly available. Across all diseases, the hypothesis-driven multivariable models always reduced Watanabe–Akaike information criterion relative to a model including only a geospatial effect (including covariates improved model fit). For the two diseases with sufficient data coverage in more than one global region (dengue in the Americas, Africa and Asia; yellow fever in Latin America and Africa) we also fitted region-specific multivariable models to examine the consistency of inferred drivers across different socio-ecological settings (Extended Data Fig. 8).

### Examining aggregate and shared driver effects across disease groups

For many infectious diseases, synergistic interactions between drivers may be necessary to align the conditions for spillover and emergence risks (for example, high livestock densities in fragmented forest landscapes for bat-borne henipaviruses^6^). Improving geospatial prediction for emergence risks requires accounting for how compound drivers align to create local foci of pathogen transmission. To examine this question we estimated the mean effect size for each driver and visualized patterns of co-occurrence between drivers, across all 31 individual modelled diseases, and for subsets of either directly transmitted (*n* = 10) or vector-borne (*n* = 17) zoonoses. For each driver, we generated a posterior distribution of the mean effect size across all diseases for which that covariate was tested. To achieve this, we calculated the mean fixed effect size using one randomly drawn posterior sample from each tested disease, and repeated this 5,000 times for each driver to build up a posterior distribution, from which we calculated median, 67% and 95% credible intervals (Fig. 4a–c). The resulting mean effect distribution therefore explicitly incorporates the variation in fixed effects uncertainty between different diseases (owing to differences in sample size and variability), with wider posterior intervals reflecting more heterogeneity in effects directionality and/or greater statistical uncertainty. We only conducted this analysis for drivers that were tested for five or more diseases, to ensure our mean estimates were not overly biased by a very small sample of diseases.

To visualize the pattern of shared drivers, we generated unipartite networks with drivers represented as nodes, and with edges between driver pairs weighted by the number of diseases for which each pair of drivers co-occurred (when both drivers had 95% credible intervals not overlapping zero; Fig. 4d–f). In parallel, to examine observed autocorrelation among putative drivers at global and regional scales, we generated a matrix of pairwise Pearson correlation coefficients between each pair of scaled covariates across 50,000 background points globally, or subsets of background points within five regions containing most of our data (North America, Latin America and the Caribbean, sub-Saharan Africa, East Asia and Pacific, and South Asia). These were used to visualize unipartite networks of pairwise driver correlations, with edges weighted by Pearson coefficient magnitude (Extended Data Fig. 4).

### Limitations of data and methodology

Because the goal of the study was to apply a general, standardized analysis framework across a variety of diseases with very different quantities and types of data, we encountered several important but irreconcilable methodological constraints that are significant to interpretation of our results, as well as to inference of spatial drivers of disease emergence more broadly. First, the datasets for many diseases (especially rare and high-consequence pathogens) are very small and spatially biased towards surveillance hotspots. We adjusted for these biases using geospatial random effects and proxies for detection processes, but these are imperfect descriptors for complex processes, and some residual confounding might remain unaccounted for (for example, health systems access is influenced locally by many factors other than proximity, and clinical index of suspicion and accessibility of diagnostics is often highly geographically variable for many rarer, non-specific febrile illnesses). Relatedly, it was often not possible to combine several data sources for the same disease without creating a geographical imbalance in the distribution of points, so our analyses were restricted mostly to datasets that were usually broad in scale but lacked granular information about transmission intensity (for example, most georeferenced outbreak datasets) or sometimes locally comprehensive at the expense of geographical breadth (for example, HCPS, which was restricted to Brazil and Argentina owing to available surveillance data, despite hantavirus infections occurring worldwide^90^). Notable exceptions where we were able to combine point and polygon data from more than one source without substantial issues included Lassa fever, CCHF, HCPS, Chagas disease (acute) and yellow fever (Extended Data Table 1 and Supplementary Table 1).

Some of the most relevant variables thought to shape risk for many high-consequence epidemic zoonoses either have not (hunting pressure outside the tropics) or cannot (wildlife trade) be translated readily into global geospatial covariates that accurately reflect their relationship to infection risk. For example, the impacts of wildlife trade and markets on disease risks can be spatially diffuse and transboundary, involving several actors at several points along commodity chains from capture to sale^91^; consequently, quantifying how these activities shape the spatiotemporal dynamics of zoonotic spillover may require substantially different analytic approaches than what is possible with this study’s geolocated outbreak event data. (However, we also refer to other work that has highlighted instances where wildlife trade has been overstated as a driver of spillover risk^15^.) Similarly, coarse modelled spatial proxies for hunting pressure such as the tropical defaunation index we used in this study^79^ probably more closely reflect commercial rather than subsistence hunting activities, even though the latter may often be more important in driving zoonotic spillover (for example, rodent hunting and exposure to Lassa fever and mpox); our study’s sparse and ambiguous results for tropical hunting pressure (Extended Data Fig. 6 and Supplementary Fig. 1) should be interpreted with this limitation in mind.

Developing a common analysis framework also led to the loss of information from some datasets, through reducing case surveillance data (with number of cases) to a binary annual outbreak indicator (losing potentially valuable information on transmission intensity). Although necessary for a standardized framework, this could feasibly erode the reliability and accuracy of inference. We therefore conducted a model comparison test, examining how reducing the data’s information content affects inferred drivers for a set of relatively well-reported diseases (four arboviruses in the continental USA using CDC ArboNET data). For each disease we compared coefficient estimates between full geospatial models of county-level case incidence, and our outbreak event risk modelling framework. County-level total case incidence across the surveillance period (2000–2020) was modelled using a negative binomial (West Nile fever) or zero-inflated negative binomial likelihood (La Crosse encephalitis, Powassan encephalitis and JCE), including an offset of log population, and a fitted geospatial random effect to account for unexplained geographical variation, again implemented using INLA. We found that most significant socio-environmental effects from a full case incidence model (reflecting transmission intensity) remained detectable even in a dataset reduced to binary outbreak occurrences with background locations (Extended Data Fig. 7). This test improved our confidence that our modelling approach is sufficient to capture key spatial drivers of risk, despite this information loss.

Nonetheless, given data sparsity for many infections, it was not possible in this standardized framework to account for temporal dimensions of causality (for example, time-specific climate or land change effects), such as by aligning covariate and case data in time, and/or adjusting for temporal patterns of detection through spatiotemporal random effects. This kind of analysis is feasible and fruitful when modelling case surveillance time series for better-surveyed infections (including some in our study such as Lyme disease or West Nile fever), but it was not possible to apply this consistently across diseases, given the extreme sparsity of outbreak data for infections such as Ebola, Marburg, Hendra and Nipah virus disease. Data sparsity also prohibited explicitly testing for nonlinear effects of drivers, although we sought to mitigate this issue by selecting covariates for individual processes whose monotonic effects combine to produce apparent nonlinear effects (for example, habitat loss and fragmentation acting together to produce peaks in spillover risk at intermediate levels of land conversion^37^). The lack of fine-scale spatiotemporal data also meant we could not disentangle elevated outbreak detection rates in urban areas, from the potential true contribution of urban social and environmental processes to risk for certain diseases; for example, human population density and urban mosquito habitats may play a significant role for *Aedes* mosquito-borne viruses that transmit between humans (dengue, chikungunya and Zika). Nonetheless, we do not expect consistent positive relationships between human density and spillover risk across all diseases in our data (many of which have rural sylvatic ecologies). Rather than solely a limitation of this study, these are more general problems for attribution of outbreak drivers for rarely documented but high-consequence infections, that currently hinders our capacity to, for example, robustly link recent deforestation to viral zoonosis outbreaks. Improving both fundamental eco-epidemiological research, and strengthening healthcare access, diagnostics and surveillance in underserved areas, will be needed to fill these gaps.

### Reporting summary

Further information on research design is available in the Nature Portfolio Reporting Summary linked to this article.

## Online content

Any methods, additional references, Nature Portfolio reporting summaries, source data, extended data, supplementary information, acknowledgements, peer review information; details of author contributions and competing interests; and statements of data and code availability are available at https://doi.org/10.1038/s41586-026-11058-6.

## Supplementary information

Supplementary Information Supplementary Figs. 1–4 and legends for Tables 1–3.

Reporting Summary

Supplementary Table 1 Disease data sources, links and access.

Supplementary Table 2 Hypothesis generation exercise form.

Supplementary Table 3 Socio-environmental driver data sources, links and access.

Peer Review File

## Source data

Source Data Fig. 2

Source Data Fig. 3

Source Data Fig. 4

Source Data Fig. 5

## Figures and Tables

**Extended Data Table 1:**
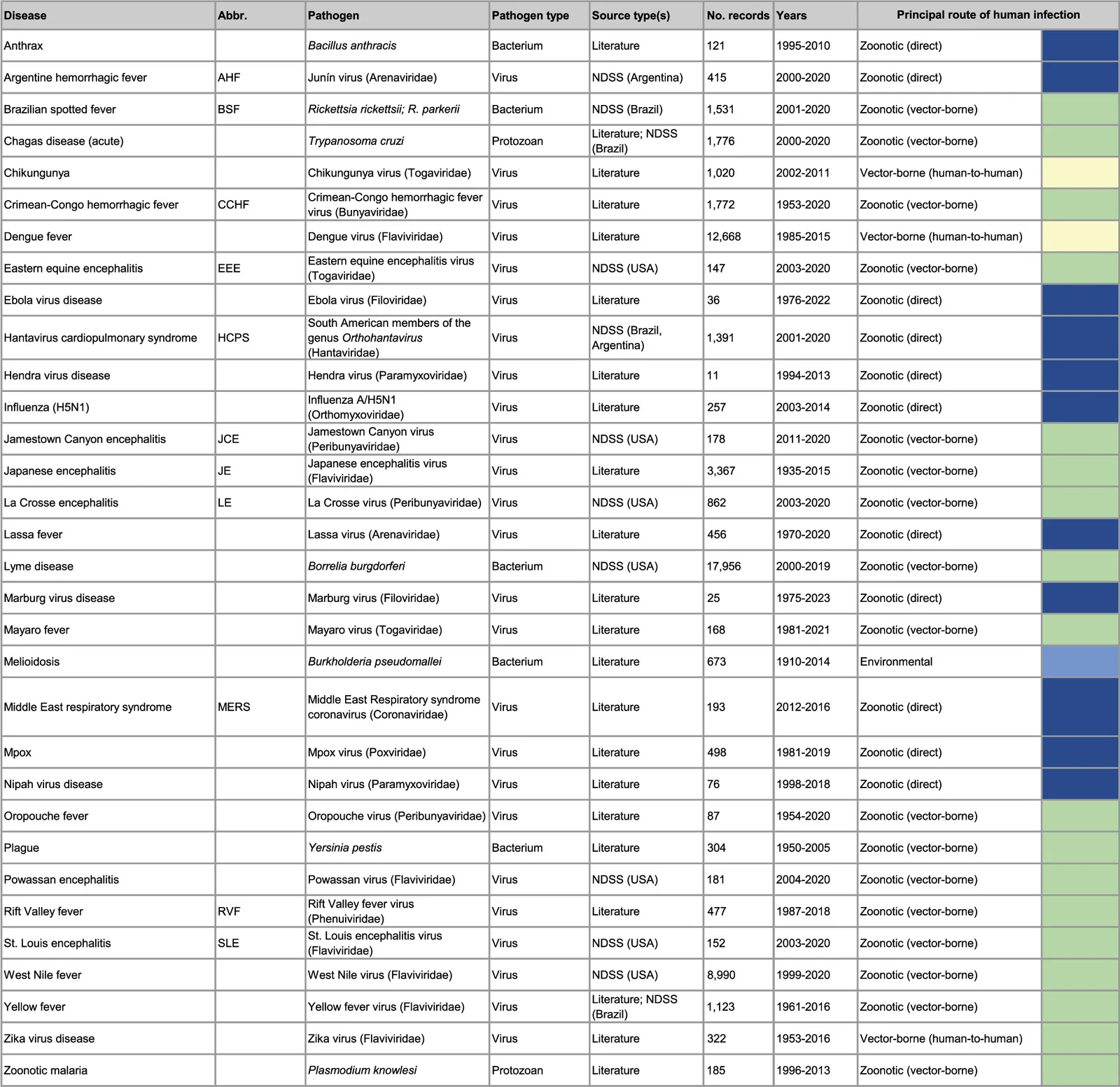
Database of outbreak events for 32 emerging infectious diseases

The table lists all diseases for which we were able to compile georeferenced human case or outbreak data, including the disease, pathogen(s), predominant transmission route to humans, number of outbreak event records and time period. A fuller set of data source descriptions with information about open accessibility for each source dataset is provided in Supp. Table 1. Most disease data were from a single source, but for several diseases we were able to combine and harmonize data from across more than one source database, as shown in the table (Extended Data Fig. 1; e.g. hantaviruses; Lassa fever).This table includes all data points across all years (including prior to 1985) and regardless of spatial precision; prior to modeling, these data were subsequently subset to post-1985 records to better harmonize with covariate layers, and records with very low spatial precision were excluded (Methods). Abbreviations: NDSS - national disease surveillance systems. Colour coding refers to human infection pathway and is aligned with colour scheme in Fig. 1.

**Extended Data Table 2:**
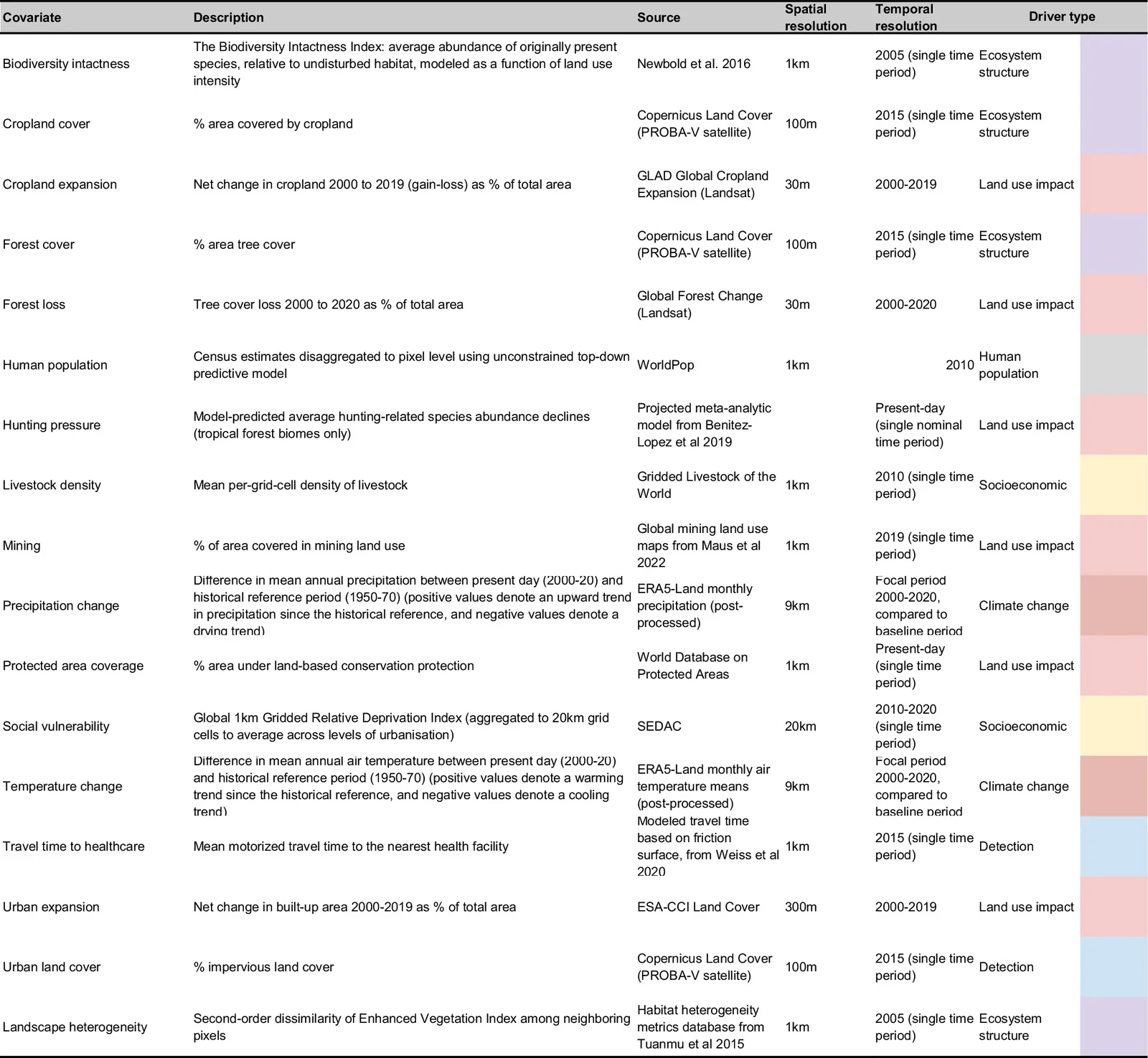
Socio-environmental covariate data sources

Table provides descriptions of the socio-environmental covariates used for each hypothesized driver, including the source, precise description, and spatial and temporal resolution. Information on open accessibility for each covariate is provided in Supp. Table 3. Colour coding denotes driver type and is aligned with all results figures.

## Data Availability

Outbreak data for the following diseases, which were openly available from scientific publications, are available at Zenodo (https://doi.org/10.5281/zenodo.20738794)^92^: CCHF, Chikungunya, dengue, Ebola, Hendra, H5N1 influenza, Japanese encephalitis, Lassa, Marburg, Mayaro, melioidosis, MERS, mpox, Nipah, RVF, Zika and zoonotic malaria. Data for US arboviral diseases (Eastern equine encephalitis, JCE, La Crosse encephalitis, Powassan encephalitis, West Nile fever, SLE) are available from the US Center for Disease Control’s (CDC) ArboNet platform (https://www.cdc.gov/vector-borne-diseases/php/arbonet/index.html). Data for Lyme disease can be accessed from the US CDC (https://www.cdc.gov/lyme/data-research/facts-stats/index.html#cdc_data_surveillance_section_2-available-data). Brazilian disease surveillance data (Brazilian spotted fever, Chagas, HCPS, yellow fever) are available through Brazil’s Notifiable Disease Surveillance System (SINAN) (https://datasus.saude.gov.br/). In cases where disease data are not public domain and were shared with us for specific use within this study (Argentine haemorrhagic fever, anthrax, HCPS (Argentina), Oropouche, plague, yellow fever), these data are available upon reasonable request from the respective data providers or corresponding authors of source publications (see Supplementary Table 1 for details). Gridded social and environmental covariate datasets are freely accessible online from their original sources (see Supplementary Table 2 for details). Source data are provided with this paper.

## Code availability

All code to reproduce this study’s results, public domain disease data and disease-specific results objects (for example, .csv files of parameter estimates; rasters of fitted geospatial effects) are available at Zenodo (https://doi.org/10.5281/zenodo.20738794)^92^.
