# Supplementary material for: The anthropogenic fingerprint on emerging infectious diseases
Source: Nature. 2026 Sep 23;658(8138):1301–9. doi: 10.1038/s41586-026-11058-6 (PMC13716837; doi:10.1038/s41586-026-11058-6)
Supplement: Supplementary file 1 [file 41586_2026_11058_MOESM2_ESM.pdf]

nature portfolio

Corresponding author(s): Rory Gibb, Sadie Ryan, Colin Carlson

Last updated by author(s): 17/6/2026

Reporting Summary

Nature Portfolio wishes to improve the reproducibility of the work that we publish. This form provides structure for consistency and transparency in reporting. For further information on Nature Portfolio policies, see our Editorial Policies and the Editorial Policy Checklist.

Statistics

For all statistical analyses, confirm that the following items are present in the figure legend, table legend, main text, or Methods section.

n/a	Confirmed	
☐	☒	The exact sample size (*n*) for each experimental group/condition, given as a discrete number and unit of measurement
☐	☒	A statement on whether measurements were taken from distinct samples or whether the same sample was measured repeatedly
☐	☒	The statistical test(s) used AND whether they are one- or two-sided<br>*Only common tests should be described solely by name; describe more complex techniques in the Methods section.*
☐	☒	A description of all covariates tested
☐	☒	A description of any assumptions or corrections, such as tests of normality and adjustment for multiple comparisons
☐	☒	A full description of the statistical parameters including central tendency (e.g. means) or other basic estimates (e.g. regression coefficient) AND variation (e.g. standard deviation) or associated estimates of uncertainty (e.g. confidence intervals)
☐	☒	For null hypothesis testing, the test statistic (e.g. *F*, *t*, *r*) with confidence intervals, effect sizes, degrees of freedom and *P* value noted<br>*Give P values as exact values whenever suitable.*
☐	☒	For Bayesian analysis, information on the choice of priors and Markov chain Monte Carlo settings
☐	☒	For hierarchical and complex designs, identification of the appropriate level for tests and full reporting of outcomes
☐	☒	Estimates of effect sizes (e.g. Cohen's *d*, Pearson's *r*), indicating how they were calculated

Our web collection on statistics for biologists contains articles on many of the points above.

Software and code

Policy information about availability of computer code

Data collection	All code used to collate and process the data are provided at the study's Zenodo archive (URL).
Data analysis	All code used to analyse the data are provided at the study's Zenodo archive (URL), which includes a demo version of the pipeline to apply to specific diseases to demonstrate functionality. Data processing and analysis was conducted in R, with the packages sf (v1.0.23), exactextractr (v0.10.1) and INLA (v.23.3.26).

For manuscripts utilizing custom algorithms or software that are central to the research but not yet described in published literature, software must be made available to editors and reviewers. We strongly encourage code deposition in a community repository (e.g. GitHub). See the Nature Portfolio guidelines for submitting code & software for further information.

Data

Policy information about availability of data

All manuscripts must include a data availability statement. This statement should provide the following information, where applicable:

- Accession codes, unique identifiers, or web links for publicly available datasets
- A description of any restrictions on data availability
- For clinical datasets or third party data, please ensure that the statement adheres to our policy

Outbreak data for the following diseases, which were openly available from scientific publications, are provided within the study Zenodo repository: CCHF, Chikungunya, dengue, Ebola, Hendra, H5N1 influenza, JE, Lassa, Marburg, Mayaro, melioidosis, MERS, mpox, Nipah, RVF, Zika, zoonotic malaria. Data for USA

arboviral diseases (EEE, Jamestown Canyon, LaCrosse, Powassan, West Nile, SLE) are available from the US Center for Disease Control's (CDC) ArboNet platform (https://www.cdc.gov/vector-borne-diseases/php/arbonet/index.html). Data for Lyme disease can be accessed from the US CDC (https://www.cdc.gov/lyme/data-research/facts-stats/index.html#cdc_data_surveillance_section_2-available-data). Brazilian disease surveillance data (BSF, Chagas, HCPS, yellow fever) are available via Brazil's Notifiable Disease Surveillance System (SINAN) (https://datasus.saude.gov.br/). In cases where disease data are not public domain and were shared with us for specific use within this study (AHF, anthrax, HCPS (Argentina), Oropouche, plague, yellow fever), these data are available upon reasonable request from the respective data providers or corresponding authors of source publications (see Supp. Table 1 for access details). Gridded social and environmental covariate datasets are freely accessible online from their original sources (see Supp. Table 2 for access details).

Research involving human participants, their data, or biological material

Policy information about studies with human participants or human data. See also policy information about sex, gender (identity/presentation), and sexual orientation and race, ethnicity and racism.

Reporting on sex and gender	NA
Reporting on race, ethnicity, or other socially relevant groupings	NA
Population characteristics	NA
Recruitment	NA
Ethics oversight	This study did not use human subjects data so was not required.

Note that full information on the approval of the study protocol must also be provided in the manuscript.

Field-specific reporting

Please select the one below that is the best fit for your research. If you are not sure, read the appropriate sections before making your selection.

☐ Life sciences ☐ Behavioural & social sciences ☒ Ecological, evolutionary & environmental sciences

For a reference copy of the document with all sections, see nature.com/documents/nr-reporting-summary-flat.pdf

Ecological, evolutionary & environmental sciences study design

All studies must disclose on these points even when the disclosure is negative.

Study description	In this study we empirically test for a general detectable fingerprint of anthropogenic drivers on the geographical distribution of human outbreaks of 32 emerging infectious diseases. We compiled existing geolocated outbreak and case data sources, as well as gridded datasets representing key socio-environmental disease drivers. To account for differing ecological characteristics across diseases and avoid testing for spurious or irrelevant associations, we generated a set of hypothesized key drivers for each individual disease through a structured form-based exercise completed by most coauthors. Across all diseases overall, and individually per disease, we applied a standardized statistical inference framework, which involved harmonization of point and polygon data and inference of the drivers of outbreak risk using geospatial logistic regression models.
Research sample	The dataset contains 58,319 unique outbreak event records, geolocated to their latitude and longitude, for 32 emerging infectious diseases worldwide. These were compiled from published disease outbreak datasets, mainly in the public domain and some provided directly by authors, and national notifiable disease surveillance systems.
Sampling strategy	Sample size for each disease was determined by the size of the dataset and historical record of outbreaks, and ranged from a small number of historical documented outbreaks (<30 for certain bat-borne epidemic infections) to very large numbers (>12,000) for well-studied infections such as Lyme disease and dengue.
Data collection	Data were collated and harmonized from published scientific datasets and national notifiable disease surveillance systems. A standardized definition of an "outbreak event" was applied (at least 1 case per named location per year) and environmental covariate values were extracted from spatial raster data in the geolocated area for each outbreak event.
Timing and spatial scale	The spatial extent of the data covers most of the globe with lowest coverage in North Africa, Europe and Central Asia. The full dataset includes data from 1910 to the present, but analyses focused on data from 1980 to present to better align disease outbreaks with available covariate data.
Data exclusions	Diseases with too few historical human outbreaks to enable stable model inference (Hendra virus) were excluded from individual disease models.
Reproducibility	Data processing, harmonization, modelling and results generation were all produced using an R code pipeline that is reproducible and shared in the study Zenodo repository.

Randomization	Samples were not randomised as we used historical locations of documented outbreaks from the scientific literature and surveillance systems, so our sample was determined by data availability.
Blinding	Blinding was not appropriate for the same reason as provided above for "Randomization".

Did the study involve field work? ☐ Yes ☒ No

Reporting for specific materials, systems and methods

We require information from authors about some types of materials, experimental systems and methods used in many studies. Here, indicate whether each material, system or method listed is relevant to your study. If you are not sure if a list item applies to your research, read the appropriate section before selecting a response.

Materials & experimental systems

n/a	Involved in the study	
☒	☐	Antibodies
☒	☐	Eukaryotic cell lines
☒	☐	Palaeontology and archaeology
☒	☐	Animals and other organisms
☒	☐	Clinical data
☒	☐	Dual use research of concern
☒	☐	Plants

Methods

n/a	Involved in the study	
☒	☐	ChIP-seq
☒	☐	Flow cytometry
☒	☐	MRI-based neuroimaging

Plants

Seed stocks	*Report on the source of all seed stocks or other plant material used. If applicable, state the seed stock centre and catalogue number. If plant specimens were collected from the field, describe the collection location, date and sampling procedures.*
Novel plant genotypes	*Describe the methods by which all novel plant genotypes were produced. This includes those generated by transgenic approaches, gene editing, chemical/radiation-based mutagenesis and hybridization. For transgenic lines, describe the transformation method, the number of independent lines analyzed and the generation upon which experiments were performed. For gene-edited lines, describe the editor used, the endogenous sequence targeted for editing, the targeting guide RNA sequence (if applicable) and how the editor was applied.*
Authentication	*Describe any authentication procedures for each seed stock used or novel genotype generated. Describe any experiments used to assess the effect of a mutation and, where applicable, how potential secondary effects (e.g. second site T-DNA insertions, mosiacism, off-target gene editing) were examined.*
