# Supplementary material for: The anthropogenic fingerprint on emerging infectious diseases
Source: Nature. 2026 Sep 23;658(8138):1301–9. doi: 10.1038/s41586-026-11058-6 (PMC13716837; doi:10.1038/s41586-026-11058-6)
Supplement: Supplementary file 2 [file 41586_2026_11058_MOESM6_ESM.pdf]

nature portfolio

Peer Review File

The anthropogenic fingerprint on emerging infectious diseases

Corresponding Author: Dr Rory Gibb

Any redactions in this file are there to maintain patient confidentiality, the confidentiality of unpublished data, or to remove third-party material.

This file contains all reviewer reports in order by version, followed by all author rebuttals in order by version.

Version 0:

Reviewer comments:

Referee #1

(Remarks to the Author)
Gibb et al. present a compelling and thorough analysis to test the influence of social and environmental drivers of outbreak risk for 32 zoonotic and vector-borne diseases globally. Using novel and varied spatial and statistical approaches, they find that a confluence of mosicac landscape and climatic (decreases in precipitation) variables influence human outbreaks at a global scale, and they disaggregate analyses based on transmission pathways and geographic regions. Overall, their findings support other global analyses and disease-specific investigations on emergence potential, but critically differ in their global scale by examining a large, compiled dataset of all recent outbreak events for each of the 32 pathogens. The figures and figure legends are clear and well presented. Their hypothesis testing follows a small-scale expert solicitation exercise (only with study authors) to target the analyses to be more biologically sound than other purely machine learning or AI approaches. Spatial occurrence of outbreaks was also found to be, unsurprisingly, associated with factors affecting detection bias as measured by healthcare access – and they make a case for continued strengthening of disease surveillance and detection systems to improve global health security. I especially appreciated the analysis of clustering of disease drivers across scales and pathogen types; and was also happy to see their sensitivity validation of using single ‘outbreaks per year’ as a response variable, vs. the richer data hidden in the # of cases per outbreak (which they also collected but didn’t use).

Overall, I found the paper to be very well written, thoughtful, with conclusions that logically follow from their statistical findings. The analyses themselves are sound, complicated but clearly presented, and the authors account for spatial reporting bias and covariance among variables. I was overall impressed with this paper that has few holes in it, but do recommend a few overarching suggestions for improvement:

1. One of the most important factors in outbreak risk was the “increasing urban land cover” for 20/30 diseases tested. While I recognized that the authors use population weighted random points as pseudo-absence points in their spatial analysis (Line 614-18), it seems that the covariation with population size and density and with this “urban cover” variable is not fully teased out. In other words, wouldn’t you expect a greater number of outbreaks in more dense human population centers/urban environments (i.e. are we not just seeing a population size effect here?), especially for the vector borne, urban cycling viruses that were tested? Human population is a difficult thing to control for given its importance as both a true driver infection risk, and a confounder influencing other socio-economic factors, and detection bias of pathogens itself. At minimum, it would be good for the authors to further discuss this relationship which isn’t fully addressed in the manuscript as is.
2. I am curious as to why the expert solicitation exercise did not include disease specific experts for the 32 pathogens, or a greater panel of global health and infectious disease experts in general. I applaud the authors for taking the approach of using participatory ranking of potential drivers to include in their hypothesis testing and models, but limiting this to only the study authors seems to be a drawback. Yes, there are a great number of experts on the paper from different disciplines, but most expert on statistical modeling and disease ecology, and less so with deep domain expertise for the individual pathogens. Giving an option of “I don’t know” on the survey was helpful, but then the rankings are based on even a smaller number of experts and prone to more biases in ranking factors for inclusion.
3. The based on the findings of precipitation and landuse/structure changes, the authors recommend that “climate policies could achieve net reductions in disease burden”. This is not a new idea, and it would be nice for more elaboration on the

specific policies that could result in gains for health; and more importantly, at what timescale might these have impact. It is easy to say slow greenhouse gas emissions now, but will that really have measurable impact on Zika or chikungunya, or RVF outbreak frequency in the coming 10, 20, 50 or more years?

(Remarks on code availability)

Referee #2

(Remarks to the Author)
The manuscript “The anthropogenic fingerprint of emerging infectious diseases” analyzes a dataset of outbreak events for 32 emerging infectious diseases to “test the influence of 16 hypothesized social and environmental drivers for outbreak risk” (71-74), aiming to understand whether “a general anthropogenic fingerprint on the spatial intensity of outbreak events can be distinguished […]; (2) whether any broad categories of environmental change are consistently implicated in outbreak events across pathogen types, transmission modes, and regions; and (3) whether there is evidence of widely-shared drivers across diseases and transmission modes that could point towards promising ecosystem-based intervention strategies.” (186-189). The authors find evidence for risks associated with habitat fragmentation and environmental factors in analyses that pool diseases and in analyses of vector-borne diseases, but little evidence for such drivers for directly transmitted zoonotic diseases (e.g. 74-81). Their analyses also highlight that outbreak intensity is strongly related to healthcare and reporting access, indicating insufficiencies of disease surveillance systems (e.g. 81-85). The study is impressive in its scope, well written, and potentially of importance for Nature’s broad audience. However, as outlined in detail below, I have several comments, both technical and conceptual, that I think would be important to address to increase the study’s robustness and ensure that findings are not artefacts of e.g. particularly well-sampled diseases. I hope the authors will find these comments helpful

1. Hypothesis-generating exercise
The authors determined covariates for their model analysis using a hypothesis-generating exercise in which “25 study authors independently ranked candidate drivers for each disease” (232-234). They then proceeded with two sets of analyses, with hypotheses/covariates selected based on either a “majority rule” or the “top-ranked” hypotheses (539-542). The authors state that they adopted this approach “to balance comprehensiveness and exhaustion” (511-512), and while in general, I do agree that this is a reasonable approach, I have a few questions and comments about the details of this exercise:
(i) Please expand on the process of how the initial 18 drivers were selected (512).
(ii) Please expand on the process of how these drivers were translated into proxy covariates (551), i.e., how choices were made regarding what specific metrics should be used in the model. Some of the choices shown in Extended Data Table 2 seem natural (e.g., % area covered by cropland), while others would benefit from additional explanation (e.g., the metric for vegetation heterogeneity). For each metric: Were final choices mostly based on data availability? Or were there other reasons? Were alternatives considered for each metric, and if so, based on what criteria were final choices made?
(iii) 527-530: The authors’ expertise spanned a wide range of disciplines, and while this certainly is beneficial for obtaining a wide range of perspectives, it also brings up the question whether authors’ opinions should be weighted equally for determining potential disease drivers? I don’t necessarily suggest that the authors alter their approach here, as any deviations might be arbitrary and only lead to small differences in the generated hypothesis sets, but I think it would still be worth commenting on why e.g., experts in “machine learning”, “microbiology”, and “public health” should be equally weighted for this exercise. And perhaps as an extension of that, I would appreciate if the authors could comment on the possible influence of confirmation bias in expert opinions on the selected drivers and, thus, the study’s results.

2. Correlations among covariates
Some of the chosen covariates (164-170, 570-588, Extended Data Table 2) are correlated by definition. This is, for example, pointed out by the authors for covariates related to land use change (687-689), and is also true for several others (e.g., per cent forest cover and per cent cropland cover). While the authors address the effects of these correlations in their discussions (e.g. Extended Data Figure 8), I am wondering whether it would be possible to formally account for such correlations in the analyses (e.g., by removing highly correlated variables) rather than just discussing their effects.

3. Choice of buffer
477-482, 596-600 – Buffers were drawn “with a radius of 5km or another custom value”. (i) Could you provide a reason for this specific scale choice and how choosing differently might influence the results? (ii) How does variance in buffer values among datapoints influence the results? Could this introduce inconsistencies of how drivers were calculated and valued for different diseases or regions?

4. Potential nonlinearities
111-113 – “In general, these studies suggest that habitat fragmentation … increases wildlife disease prevalence30” However, reference 30 actually states that “the highest spillover risk occurs at intermediate levels of habitat loss, whereas the largest, but rarest, epidemics occur at extremes of land conversion”. Please rephrase and consider any consequences for the statistical modelling approach: in particular, what if the effects of drivers were not linear but e.g., hump-shaped with intermediate metric values providing the highest risk? Could that pose problems for the logistic regression analyses of the paper?

5. Implications of some diseases being much more strongly represented in the data than others

The most sampled diseases (Lyme, Dengue, West Nile) seem to be making up more than half of the data, and as such, seem to be driving the results of the global analysis shown in Figure 2. These models are e.g. referred to by the authors as "disease-agnostic", but given this imbalance in sample sizes, I wonder whether they indeed are "disease-agnostic" or whether they just represent the results for the most commonly sampled diseases. Contrasting the effect sizes shown for individual diseases (Fig. 3) vs those of the global model (Fig. 2) might provide more insight into this. Alternatively, I wonder whether it would be insightful to rerun the analyses while subsampling each disease to provide more comparable sample sizes?

6. Focus on effect size
211-219, Fig. 2c – (i) Given the number of datapoints, and in particular the number of datapoints from a well-sampled diseases, it is perhaps not surprising that effects of social/ecological risk factors were detected and that these aligned with current consensus on likely risk drivers (219-222). However, effect sizes are small for some drivers, as also noted by the authors themselves, so I would recommend discussing these results not just in terms of detectability but also in terms of effect sizes, as well as discuss to what degree these results could be driven by the overrepresentation of some diseases (cf. also point 6 above).

Minor Comments

161 – A few references are from preprint servers, such as bioRxiv. I will defer to the editor regarding the journal's policy regarding the use of such references but think that at least some of these might be replaceable by fully peer-reviewed sources.

178-181 – "including continuous geospatial random intercepts": although I understand what the authors mean, I think it would still be beneficial to provide the non-specialized reader with a one-sentence intuitive explanation of what this term does in the analyses.

213-216 – Some of the found effects seem to be contradicting one another, e.g. higher risk with higher livestock density as well as with higher biodiversity intactness and protected area coverage. I think this would deserve additional discussion, please also see my comments above about covariate correlations.

379-381 – I agree with the statement that improving surveillance would help address data gaps, but I wonder if the authors could provide more specifics regarding what is missing the most / would be the most valuable to collect in the context of their study, so to guide such surveillance efforts.

387 – The notion of "wicked problem" may be familiar to some, but I suspect not to everyone. I suggest a reference or rewording.

460 – Not clear what you mean by "administrative level 1 or 2". Please define.

535-536 – "but we do not recommend this as a template for a rapid, large-scale survey exercise" I'm not sure I see the relevance of this statement and suggest deleting

606-613 – Please clarify the procedure regarding how the number of background points were chosen, and why this was exactly "between 2 and 8". The sentence "Guidelines on…" seems to suggest that while guidelines exist, something else might have been done here, so this leaves me confused regarding the correctness and meaning of these specific numbers.

Fig 1a – Some of the colors on the map do not seem to match the color code below it. E.g., most of the U.S. seems to have dark blue dots, but there is no dark blue color in the code. Could this be due to dots with different colors overlapping? If so, it might be helpful to provide additional supplementary maps that do not have this issue, e.g., by separating commonly overlapping diseases

Extended Data Tables 1 and 2: Not clear what the color code in the last column is referring to. Please define.

(Remarks on code availability)
Please note: I have reviewed all aspects of the manuscript, except for the code. The reviewer instructions specified that:

>> It would be most helpful if you could take a look at the code provided by the authors and comment on whether it provides sufficiently clear information and documentation that would make it possible to test the code and replicate the paper's findings. We would also be grateful if you, or someone in your group, could look at the code more closely and verify that it can indeed be run.

but, while all code seems accessible and clearly organized, I typically do not program my own work in R, and as such am not well-suited for reviewing their code. I hope one of the other reviewers was able to provide this expertise.

Referee #3

(Remarks to the Author)
Summary:

In this paper, the authors study the socio-environmental drivers of disease outbreaks. Global data are gathered for several diseases which are analyzed using a spatial statistical model. The dataset and analysis are impressive and the results may be useful for policy development. The authors' attention to detection bias and the clear and fair discussion of the associated limitations of what can and cannot be inferred (i.e., whether distance to a health care facility affects detection, outbreaks or both) from the analysis is laudable. Overall, I feel this paper is a nice contribution, but I do have a few comments.

Major comments:

(1) The assumption that the same model applies across time and continent is very strong and should be tested. You could for example fit the model separately by continent and determine if the covariates effects are similar across fits.

(2) The use of the survey to select covariates is unconventional so I feel it should be further justified and explained. First, I did not see a clear statement that the survey was conducted before the survey participants had looked at the data or preliminary results. Also, the selection process to form the panel should be make more explicit. Finally, the process of gathering information from other researchers is a common way to select variables, but it is also not clear to me why the survey of 25 experts is preferred over a systematic literature review.

Minor comments:

(3) Towards the end of the Introduction you say that "random intercepts were included to account for unexplained macro-scale patterns of data availability", but the random intercepts could also capture unexplained variation in the true likelihood of an outbreak.

(4) In the second paragraph of the "Shared drivers…" section, you discuss "interactions", but actually I think "co-occurrence" in the model as used later is more appropriate. Interaction usually means that the product of covariates is included so that the effect of one can be modified by the other.

(5) In the "Statistical Modeling" section, tuning the spatial covariance parameters "via visual inspection" is too informal. I would at least be more descriptive about what you were looking for, and preferably this would be replaced a more rigorous approach such as WAIC comparisons. Also, I would call WAIC a "model comparison metric" rather than a "model adequacy metric" since it cannot be used to conclude any model is adequate, other than it fits better than other candidates.

(Remarks on code availability)

Version 1:

Reviewer comments:

Referee #1

(Remarks to the Author)
I thank the authors for addressing each of my primary concerns, and commend them on the new analysis, figures, and revised text that was responsive to each of the reviewers' concerns. I have no further suggested changes.

(Remarks on code availability)

Referee #2

(Remarks to the Author)
Thank you to the authors for carefully responding to all my previous queries. The additional clarifications and analyses are tremendously helpful and address all my previous concerns. I especially appreciate the implementation of the new subsampling strategy for the disease-agnostic model and think that this has substantially improved the robustness of the analyses. I also appreciate the new analyses regarding the sensitivity of their results to buffer size assumptions, which again help with demonstrating robustness. Furthermore, I think that the additional details and analyses regarding the hypothesis generation exercise are very helpful, and in particular, I agree with the authors that the inclusion of the "Any author" rule further demonstrates robustness of their results. The new meta-analysis of driver effect sizes, along with the regional analyses, are insightful and help to further tease apart key patterns.

Overall, I think this is an impressive and insightful study that sheds new light on how environmental and social covariates drive global disease outbreak risks, using an extensive global dataset. The analyses are well-structured, thorough, and impressive in their scope. The methods and conclusions seem robust, and uncertainties and limitations are well-identified and discussed. The article is well-written and the analyses are, despite their complexity, easy to follow. I only have a few minor suggestions that I list below:

Line 72: “adjusting for” -> I suggest “accounting for”

75: “as well as” -> “along with”

75-76, 262-265, 308-309: In each of these places, the authors discuss the effects of climate drying on the outbreak risks of dengue and some other diseases (e.g., “This combination of factors, as well as long-term declining trends in precipitation, share strong impacts across multiple vector-borne diseases (e.g. dengue, West Nile and Lyme disease”). Figure 3 and Extended Data Figure 6 suggest general trends, as stated by the authors (262-265), but I do think it would be important to provide additional discussion on the general direction of effects, exceptions to these trends, and possible mechanistic explanations. I suggest this because, naively, one might assume that more precipitation would result in more standing water, and therefore more mosquito habitat and more disease – but it seems you’re seeing the opposite effect. I wonder whether considering why some diseases show opposing trends than others (most notably West Nile, Supp. Fig. 1) could be informative in explaining these observations. Also, please clarify in Extended Data Table 2 how “Precipitation Change” is defined. Precipitation in 2000-2020 minus that in 1950-1970, or vice versa? The direction is not clear from the descriptions in the second and fifth columns.

121-127: Here, you discuss previous studies that have examined “ecological correlates of human disease emergence”, summarize their findings (“These studies have found that land use change, agricultural expansion, biodiversity hotspots and global travel are widely associated with observed geographic hotspots of disease emergence, 123-125), and outline how their approach differs from yours (125-135). I understand space restrictions might be limiting, but if possible, I think it would be interesting to refer back to this after you have presented your results (e.g., in your Conclusions, 404-427) and briefly discuss whether / how the ecological correlates in these two contexts (previous studies on emergence vs your study on ongoing risk) are similar or not.

131: The sentence “Assigning outbreak drivers solely based on expert opinion…” does not seem to follow logically from the previous sentences (which talk about the limitations of emergence data) -> reword to clarify link.

259: “Outbreak risk was higher in fragmented and forested landscapes […], with common and almost always positive effects of landscape heterogeneity.” Here, and in a few other places (e.g., 275, 305, 310, 571), I noticed that I had to stop and remind myself what was meant by “positive”. While in most places the manuscript is unambiguous (e.g., “more heterogeneity means higher risk”), there are some places where I wondered whether general readers could misinterpret such statements in a colloquial sense (“heterogeneity brings positive impacts, i.e., lower risk”). In Supplementary Table 2, you actually state that “Make sure to think carefully about what sign means for the direction of each variable; for example…”, and I suggest that a similar clarification could be helpful to have in the main text at an appropriate place (along with a careful review of all places where the words “positive” and “negative” are used to ensure that statements cannot be misunderstood).

895: “Borrelia burgdorferi or West Nile fever” -> I suggest “Lyme or West Nile fever” (i.e., using either the name of the disease or the name of the causative agents consistently would help avoid confusion)

1145: “diseases names” -> “disease names”

1154: “author” -> “authors”

1171-1172: “diseases are ordered from left to right by number of outbreak records (lowest to highest)” -> I think this should be “highest to lowest” (Lyme is on the left, Marburg is on the right)

1410 & 1416: “B-F” should be “B-G”

(Remarks on code availability)

Referee #3

(Remarks to the Author)
Thank you for the thoroughly responsing to my comments. I am satisfied with the reviewed manuscript.

(Remarks on code availability)
I downloaded the data and code from github and run the small example on my laptop. The data and code are in order and the example worked fine.

Version 2:

Reviewer comments:

Referee #2

(Remarks to the Author)
I thank the authors for carefully addressing each of my comments. I have no further concerns or suggestions.

(Remarks on code availability)

Open Access This Peer Review File is licensed under a Creative Commons Attribution 4.0 International License, which permits use, sharing, adaptation, distribution and reproduction in any medium or format, as long as you give appropriate credit to the original author(s) and the source, provide a link to the Creative Commons license, and indicate if changes were made.
In cases where reviewers are anonymous, credit should be given to 'Anonymous Referee' and the source.
The images or other third party material in this Peer Review File are included in the article's Creative Commons license, unless indicated otherwise in a credit line to the material. If material is not included in the article's Creative Commons license and your intended use is not permitted by statutory regulation or exceeds the permitted use, you will need to obtain permission directly from the copyright holder.
To view a copy of this license, visit https://creativecommons.org/licenses/by/4.0/

Response to referees for Gibb et al. 'The anthropogenic fingerprint on emerging infectious diseases'

<u>Editors' comments:</u>

Firstly, I must sincerely apologise for the unusually lengthy review process for your manuscript, "The anthropogenic fingerprint on emerging infectious diseases". It has now been seen by 3 referees, whose comments are attached below. While they find your work of potential interest, as do we, they have raised important concerns that in our view need to be addressed before we can consider publication in Nature.

All three reviewers have made comments about the survey that was used as the basis for hypothesis generation. The reviewers find it unconventional and a possible source of bias. You would need to convince the reviewers that this approach did not bias the study outcome and ensure that the hypotheses generated are generally representative of what would have been selected had a systematic literature review been done. You would also need to provide more detail about how the survey was done. For example, how did you arrive at the 31 co-authors? Why did only 25 answer the questions? How did you validate the questions and make sure that people all understood the same thing from the questions? The reviewers have also raised a number of other technical concerns that should be addressed.

We thank the editors and reviewers for their positive comments and constructive suggestions for how to improve the manuscript, and for the opportunity to revise and resubmit our study to Nature. We have now addressed these concerns in a significantly revised version which has substantially improved the clarity of the findings. We summarise the major changes in response to the editors below, then provide a point-by-point response to each reviewer's specific concerns. Line numbers and figure/table references refer to the revised manuscript.

Summary of major changes:

1. **<u>Addressing concerns around bias from the hypothesis generation exercise.</u>** We have made significant efforts to more fully explain the scope and limits of the hypothesis generation exercise, and to demonstrate quantitatively that it does not systematically bias our results. We have particularly emphasised that this exercise was not designed as a survey; we had specifically avoided using that terminology in the original manuscript. Rather, we designed the exercise to enable the participatory generation of hypotheses across our geographically dispersed author team of disease-specific experts (whereas under different circumstances we could have workshopped hypotheses in person). We have provided fuller detail about how the exercise was designed and tested (**lines 550-578**); the disease-specific expertise that the coauthors contribute to this exercise (**lines 587-595**4; and the reasons for 6 authors not participating (2 not being infectious disease experts; 3 being unavailable at time of exercise; 1 joining as an author after the exercise was complete) (**lines 578-582**). We also acknowledge that some of the discussion in the original manuscript was confusing in implying our hypotheses are representative of expert opinion beyond the coauthor team. We have therefore edited these sections of text to aid in clarity of interpretation (**lines 267-286**).

   Crucially, to ensure that this approach has not biased our results, we have also added

an extra set of modelling sensitivity analyses using a lowest-stringency criterion for hypothesis inclusion ("any author"). This approach retains 97% of potential disease-driver combinations to test (compared to 80% and 50% for the majority rule and top-ranked criteria), i.e. solely screens out highly implausible covariates and allows the patterns in the data to speak (**Methods**). We find that the overall results are extremely similar when we take this minimally stringent approach. This strongly suggests that our overall findings are not systematically biased by the hypothesis exercise, and that it is unlikely that an alternative approach to driver hypothesis selection would have led to substantially different conclusions.

Nonetheless, both Reviewer 1 and 2 agreed that a key contribution of our study is this rigorous hypothesis-driven approach, so for this reason, we still present the "majority rule" models as the main results. This criterion tests a focused set of key hypotheses per disease, which is especially important for diseases with a smaller sample size. The "any author" and "top ranked" results are presented as supplementary sensitivity analyses (**Supp. Figures 2 and 3**) and discussed in the text (**lines 239-240; 283-286; 601-616**).

2. **<u>Accounting for differences in disease sample sizes in the estimates of multi-disease drivers.</u>** Reviewer 2 raised the important concern that the results of the global disease-agnostic model may have been biased by variation in sample sizes across diseases. We agree that this was an important consideration we missed, and have made two significant changes to robustly estimate average driver effects across multiple diseases. Firstly, we have implemented a subsampling approach to the global models, estimating the posterior driver effects across an ensemble of 100 submodels fitted to balanced subsamples of the dataset (each containing equal sample sizes per disease). These results are presented in the updated **Figure 2** and **Extended Data Figure 4**, and better capture uncertainty in the size and directionality of driver effects, while providing similar overall findings.

   Secondly, we have taken a "bottom-up" approach and conducted a meta-analysis of mean driver effect sizes across all the disease-specific models, which accounts for variation in statistical uncertainty in fixed effects between diseases (**Figure 4a-c, lines 327-333; 809-822**). Paralleling the shared driver networks, we conducted this analysis for all diseases, and then for subsets of either vector-borne zoonoses or directly transmitted zoonoses. The results of these analyses further strengthen our overall conclusions: that multiple vector-borne zoonotic diseases emerge at the nexus of cities, fragmented forests and areas experiencing long-term precipitation change, but in contrast there is little evidence for shared or consistently directional driver effects for directly-transmitted zoonoses, including major epidemic and pandemic threats.

3. **<u>Improving the visual coherence of the results across both multi-disease and individual disease analyses.</u>** To ensure that the results are more interpretable and follow a standard visual language throughout the paper, we have implemented a new figure design and colour scheme system for driver types. Each figure now contains background shading to denote the class of each driver, and these colours are consistent across all the Figures and Extended Data items. We have also adjusted Figure 3 to more clearly show the size, significance and heterogeneity in posterior mean

driver effects across individual diseases.

4. **Incorporating region-specific analyses for global multi-disease and individual disease models.** As Reviewers 2 and 3 note, an important but underexplored finding in the manuscript is that the nexus of emerging disease drivers – their intensity and spatial clustering – differs significantly between different geographic regions (**Extended Data Figure 3**). To explore the potential implications of this for regional outbreak risks, we implemented region-specific versions of the global multi-disease model (**Extended Data Figure 4**), and also implemented region-specific models for the two diseases with sufficiently geographically widespread data (dengue in Americas, Africa and Asia; yellow fever in Africa; **Extended Data Figure 8**). Overall we find evidence that ecosystem structure and detection drivers are generally similar across regional contexts, but with some key differences, including in the strength of livestock density effects.

Referees' comments:

Referee #1 (Remarks to the Author):

Gibb et al. present a compelling and thorough analysis to test the influence of social and environmental drivers of outbreak risk for 32 zoonotic and vector-borne diseases globally. Using novel and varied spatial and statistical approaches, they find that a confluence of mosicac landscape and climatic (decreases in precipitation) variables influence human outbreaks at a global scale, and they disaggregate analyses based on transmission pathways and geographic regions. Overall, their findings support other global analyses and disease-specific investigations on emergence potential, but critically differ in their global scale by examining a large, compiled dataset of all recent outbreak events for each of the 32 pathogens. The figures and figure legends are clear and well presented. Their hypothesis testing follows a small-scale expert solicitation exercise (only with study authors) to target the analyses to be more biologically sound than other purely machine learning or AI approaches. Spatial occurrence of outbreaks was also found to be, unsurprisingly, associated with factors affecting detection bias as measured by healthcare access – and they make a case for continued strengthening of disease surveillance and detection systems to improve global health security. I especially appreciated the analysis of clustering of disease drivers across scales and pathogen types; and was also happy to see their sensitivity validation of using single 'outbreaks per year' as a response variable, vs. the richer data hidden in the # of cases per outbreak (which they also collected but didn't use).

Overall, I found the paper to be very well written, thoughtful, with conclusions that logically follow from their statistical findings. The analyses themselves are sound, complicated but clearly presented, and the authors account for spatial reporting bias and covariance among variables. I was overall impressed with this paper that has few holes in it, but do recommend a few overarching suggestions for improvement:

Thank you for the positive comments and suggestions for improving the clarity and discussion of the findings. We have implemented substantial changes to address all of your suggestions, including further exploring and more clearly discussing the role of cities in driving bias versus true changes in the risk landscape, and examining the sensitivity of results to the hypothesis

exercise.

1. One of the most important factors in outbreak risk was the “increasing urban land cover” for 20/30 diseases tested. While I recognized that the authors use population weighted random points as pseudo-absence points in their spatial analysis (Line 614-18), it seems that the covariation with population size and density and with this “urban cover” variable is not fully teased out. In other words, wouldn’t you expect a greater number of outbreaks in more dense human population centers/urban environments (i.e. are we not just seeing a population size effect here?), especially for the vector borne, urban cycling viruses that were tested? Human population is a difficult thing to control for given its importance as both a true driver infection risk, and a confounder influencing other socio-economic factors, and detection bias of pathogens itself. At minimum, it would be good for the authors to further discuss this relationship which isn’t fully addressed in the manuscript as is.

Thank you for raising this issue, which we agree would benefit from being unpacked more fully. The “urban cover” covariate is the one of the most challenging to interpret due to the multiple pathways through which urbanisation can influence observed disease risk (e.g. changes in human density, outbreak detection, host and vector communities, hydrology etc).

On balance, we feel that the evidence in our study supports the widespread urban influence being principally a detection bias effect, at this global and across-disease scale. We draw this conclusion because the urban bias spans a wide diversity of diseases with very different transmission ecologies, including known diseases of major urban centres (e.g. dengue and chikungunya), and those primarily considered rural or peripheral (e.g. Brazilian spotted fever, CCHF, Chagas disease, hantaviruses, LaCrosse encephalitis, melioidosis, Lassa fever, Argentine haemorrhagic fever, Rift Valley fever, anthrax, Mayaro etc; Extended Data Figure 6). Positive human density dependence (i.e. more outbreaks where more people are) is likely to play a key role for certain infections, but principally those with extended human-to-human transmission chains, which are represented by only 3 diseases in our study (dengue, chikungunya and Zika). In contrast, most of the diseases in our dataset spill over to humans from rural sylvatic ecologies, i.e., risk depends on sufficient populations of wildlife, livestock and vectors. We do not necessarily expect a consistently positive relationship between human density and spillover risk for many of these diseases, given that human population may not scale with the force of infection; for example, agricultural diseases like anthrax are associated with rural livestock keepers, and risk decreases in population-dense urban environments (Carlson et al., 2019). Our comparison of outbreak with incidence models for arboviruses in the U.S. supports this interpretation (**Extended Data Figure 7**): we find that the urban bias is notably stronger for outbreak risk (i.e., whether cases occur and are detected) than for incidence (i.e. the number of cases), and for the diseases with more rural host communities (LaCrosse, Jamestown Canyon) the effect of cities on disease incidence is actually slightly negative.

This suggests that the widespread nature of the urban effect is principally linked to detection rather than true increases in spillover. Emerging disease cases that arise near cities with higher availability of healthcare and diagnostic laboratories are more likely to be detected, and - via surveillance data aggregation processes - their geographic locations may also be more likely to be attributed to the nearest town or city (Weiss et al 2020, Messina et al. 2015). Our findings emphasise the importance of disentangling these processes, but this would require leveraging much finer-scale geographic data on infections than is possible at our study’s global extent

(e.g. individual line lists within cities). We have now more fully discussed these issues in the manuscript (**lines 254-258; 291-293; and 900-906**).

Nonetheless, we agree that other factors - including population density - probably contribute to the urban effect for certain diseases. For example, dengue has the strongest urban effect - which is consistent across three geographic regions (**Extended Data Figure 8**) - and Zika and chikungunya among the strongest, suggesting a role of human population density, as well as of urban land cover in facilitating *Aedes aegypti* and *Ae. albopictus* breeding habitat.

To understand the consistency of the urban effect further (as well as other drivers), we also added a new analysis to examine regional variability in inferred drivers for dengue and yellow fever. Interestingly, we find that yellow fever shows a strong urban effect in Africa, but none in South America (**Extended Data Figure 8**). This is consistent with the presence of urban transmission cycles for yellow fever in Africa, and their documented absence in South America (although might equally reflect regional differences in detection capacities). Additionally, the strong evidence for a "nexus" effect of cities, fragmented landscapes and forests we find for vector-borne zoonoses (**Figure 4**) suggests that true risk may often be highest in these interface zones around the peripheral and suburban areas of cities. We have discussed the potential role of additional drivers of urban effects on **lines 900-906**.

- Carlson et al. 2019. The global distribution of Bacillus anthracis and associated anthrax risk to humans, livestock and wildlife. Nature Microbiology https://doi.org/10.1038/s41564-019-0435-4
- Weiss et al. 2022. Global maps of travel time to healthcare facilities. Nature Medicine https://doi.org/10.1038/s41591-020-1059-1
- Messina et al. 2015. A global compendium of human Crimean-Congo haemorrhagic fever virus occurrence. Sci. Data. https://doi.org/10.1038/sdata.2015.16

2. I am curious as to why the expert solicitation exercise did not include disease specific experts for the 32 pathogens, or a greater panel of global health and infectious disease experts in general. I applaud the authors for taking the approach of using participatory ranking of potential drivers to include in their hypothesis testing and models, but limiting this to only the study authors seems to be a drawback. Yes, there are a great number of experts on the paper from different disciplines, but most expert on statistical modeling and disease ecology, and less so with deep domain expertise for the individual pathogens. Giving an option of "I don't know" on the survey was helpful, but then the rankings are based on even a smaller number of experts and prone to more biases in ranking factors for inclusion.

We agree it is critical to ensure the study findings are not biased by the hypothesis generation exercise.

Firstly, on the relevance of our author team's expertise: our goal was to ensure we tested realistic disease-specific hypotheses for each disease, with the particular aim of screening out ecologically implausible drivers (e.g. wildlife hunting for West Nile virus). To avoid the potential biases created if solely the lead analyst developed these hypotheses, we felt it vital to distribute this role across our coauthor team, in order to leverage the team's diversity of disease-specific expertise. Our team includes a significant number of world-leading experts on many of the infections included in this study, who have published research on the biology, virology, ecology and epidemiology of specific diseases including anthrax (CJC, JKB, SJR), rodent-borne arenaviruses and hantaviruses (RG, KEJ, DWR, EAE, RLM, SNS, AS, BAH), Chagas disease

(MPF), filoviruses (BAH, SNS, DP, KEJ, DWR), urban *Aedes*-borne arboviruses (dengue, chikungunya and Zika; RG, SJR, CAL, JPM, EON, CJC), zoonotic mosquito-borne arboviruses (RG, MC, GFA, BAH, AK, JL, DR-A, CJC), CCHF and other tick-borne infections (RG, KEJ, DP, JPM), avian influenza viruses (MW, BAH, AK), bat-borne henipaviruses (DJB, DP), Lyme disease (SNS), melioidosis (DL), MERS (SNS), mpox and related orthopoxviruses (DP, SNS, ENH, AK), and plague (BVS, CJC). Indeed, a large proportion of authors joined the study specifically to contribute outbreak data and domain-specific expertise for individual diseases. Almost all authors contributed to the exercise, with 6 non-participants either because they were unavailable at time of exercise (x3; JKB, EON, MW), joined the study authorship after the exercise was completed (x1; AS), or do not have disease-specific expertise and instead contributed to covariate design (x2; CT, JM). We agree that these details were not sufficiently fully explained in the manuscript, so we have updated the Methods text accordingly (**lines 550-599**).

Secondly, to respond to the question of why we did not survey a wider panel of experts. We agree an expert solicitation survey would be interesting in itself, however, our hypothesis exercise was intended to facilitate the modelling analysis, rather than stand as its own analysis, and we made efforts to avoid using the terminology of a survey in the manuscript. Our aim was the participatory development of hypotheses with our author team, so we developed the hypothesis matrix and guidance for this purpose, as this was the most logistically feasible way to achieve this aim with such a geographically widespread authorship. We have ensured that this is more precisely described in the Methods (**lines 564-570)**. However, we acknowledge that, in the discussion of our results, we had sometimes described the hypothesis exercise outcomes as if they reflected expert opinion more widely. This was misleading and we have now modified the main text to avoid this (**lines 267-286**).

Thirdly, on whether our hypothesis generation approach could have systematically biased the results of the study: this question was raised by multiple reviewers and so has been a major focus of our revision. We have now tested this empirically via an additional set of sensitivity analyses: the updated manuscript now includes three different sets of hypotheses generated using criteria of varied stringency: “any author” (minimally stringent and only requires a single author to hypothesise an effect; retains 97% of disease-driver combinations), “majority rule” (retains 80% of disease-driver combinations), and “top-ranked” (retains 50% of disease-driver combinations). The newly-added “any author” criterion tests nearly all the disease-driver relationships in multivariate models - only filtering out extremely implausible combinations and highly collinear variables - so results are primarily driven by the patterns within the data, with minimal influence of the hypothesis exercise. When we compare the results across these three criteria, we find they are qualitatively and quantitatively extremely similar (**lines 284-286 and 601-616**). This demonstrates that we would have recovered the same findings irrespective of the hypothesis consensus approach, i.e. the patterns in the data are strong and consistently recoverable. Nonetheless, we agree that a strength of our study is its hypothesis-led inferential approach, so we present the results of the “majority rule” criterion as the lead findings (**Figures 3 and 4**), and provide results under the other criteria as supporting information (**Supp. Figures 2 and 3**).

3. The based on the findings of precipitation and landuse/structure changes, the authors recommend that “climate policies could achieve net reductions in disease burden”. This is not a new idea, and it would be nice for more elaboration on the specific policies that could result

in gains for health; and more importantly, at what timescale might these have impact. It is easy to say slow greenhouse gas emissions now, but will that really have measurable impact on Zika or chikungunya, or RVF outbreak frequency in the coming 10, 20, 50 or more years?

We agree this is an important clarification to make in terms of the actionability of results on this topic. In the long term, there is a substantial body of evidence that future climate change pathways assuming higher levels of mitigation (e.g. RCP2.6) are likely to substantially reduce the burden of many vector-borne diseases compared to hotter futures (e.g. dengue; Childs et al. 2025, Messina et al. 2019). Our study adds significantly to this body of evidence both by linking recent historical climate change (rather than future predicted change) to significant impacts on risk; and also for showing this is a widespread phenomenon across multiple vector-borne infections. However, outbreaks are multi-causal and the effects of climate mitigation policies on risk will likely be felt over longer time periods (i.e. years to decades) than the effects of certain social, ecological or health-systems-based interventions, that can have more immediate effects.

We have expanded the discussion of this issue in the Conclusion section to clarify the complementary nature – but also different timescales – of climate mitigation policies and other multisectoral approaches to reduce the burden of vector-borne disease:

"We find evidence of a strong and consistent anthropogenic fingerprint on vector-borne diseases, suggesting that over the coming decades, risk might be likely to increase in the absence of a substantial shift in human activities. At the local scale, ecosystem-, awareness-, and health system-based interventions could reduce the burden of vector-borne diseases with immediate effect; over longer timescales, efforts to mitigate climate change will help slow the rising burden and geographic expansion of many mosquito- and tick-borne diseases, with the greatest impacts felt over the coming decades" (**lines 410-416**).

- Childs et al. 2025. Climate warming is expanding dengue burden in the Americas and Asia. PNAS https://doi.org/10.1073/pnas.2512350122
- Messina et al. 2019. The current and future global distribution and population at risk of dengue. Nature Microbiology https://doi.org/10.1038/s41564-019-0476-8

Referee #2 (Remarks to the Author):

The manuscript "The anthropogenic fingerprint of emerging infectious diseases" analyzes a dataset of outbreak events for 32 emerging infectious diseases to "test the influence of 16 hypothesized social and environmental drivers for outbreak risk" (71-74), aiming to understand whether "a general anthropogenic fingerprint on the spatial intensity of outbreak events can be distinguished [...]; (2) whether any broad categories of environmental change are consistently implicated in outbreak events across pathogen types, transmission modes, and regions; and (3) whether there is evidence of widely-shared drivers across diseases and transmission modes that could point towards promising ecosystem-based intervention strategies." (186-189). The authors find evidence for risks associated with habitat fragmentation and environmental factors in analyses that pool diseases and in analyses of vector-borne diseases, but little evidence for such drivers for directly transmitted zoonotic diseases (e.g. 74-81). Their analyses also highlight that outbreak intensity is strongly related to healthcare and reporting access, indicating insufficiencies of disease surveillance systems (e.g. 81-85). The study is impressive in its scope, well written, and potentially of importance for Nature's broad audience. However, as outlined in detail below, I have several comments, both technical and conceptual, that I

think would be important to address to increase the study's robustness and ensure that findings are not artefacts of e.g. particularly well-sampled diseases. I hope the authors will find these comments helpful

Many thanks for these suggestions for improving the technical rigor and precision of our study. We have implemented nearly all of these suggestions, and in particular the valuable idea to redesign the global "disease-agnostic" models to ensure a balanced representation of data from across each disease. This has significantly improved the clarity and estimates of uncertainty in driver effects across all diseases (**Figure 2**) and subsets by region (**Extended Data Figure 4**). We hope these updates to the manuscript have clearly demonstrated that the findings are robust and not artefacts of variation in sampling across diseases.

1. Hypothesis-generating exercise
The authors determined covariates for their model analysis using a hypothesis-generating exercise in which "25 study authors independently ranked candidate drivers for each disease" (232-234). They then proceeded with two sets of analyses, with hypotheses/covariates selected based on either a "majority rule" or the "top-ranked" hypotheses (539-542). The authors state that they adopted this approach "to balance comprehensiveness and exhaustion" (511-512), and while in general, I do agree that this is a reasonable approach, I have a few questions and comments about the details of this exercise:

(i) Please expand on the process of how the initial 18 drivers were selected (512).

We aimed to select as diverse and representative as possible a set of socio-ecological factors that are considered major proximal drivers of emerging disease outbreaks. We started with major driver categories: social/socioeconomic; human-animal contact interfaces including food systems; landscape structure; anthropogenic stressors on biodiversity and landscapes; and climate change. Then, the study leads – who have extensive combined expertise with emerging diseases and the associated literature (RG, SJR and CJC) – brainstormed specific drivers under each category. Refining these covariates, and excluding drivers whose influence is likely too dynamic to be detectable within spatial outbreak data (e.g. climate extreme events and wildfires), produced the final list of drivers we considered for this study. We have explained this more fully in the text (**lines 550-562**).

(ii) Please expand on the process of how these drivers were translated into proxy covariates (551), i.e., how choices were made regarding what specific metrics should be used in the model. Some of the choices shown in Extended Data Table 2 seem natural (e.g., % area covered by cropland), while others would benefit from additional explanation (e.g., the metric for vegetation heterogeneity). For each metric: Were final choices mostly based on data availability? Or were there other reasons? Were alternatives considered for each metric, and if so, based on what criteria were final choices made?

We have expanded the Methods text to more fully explain the process of translating drivers into covariates (**lines 635-648**). To summarise:

(1) For most drivers there was an intuitive match to proxy covariates, with a natural single metric to use, e.g. % land cover variables, average climate change from reference to present-day period, livestock density, tree cover loss, cropland and urban expansion, etc. We selected ERA5-Land for climate due to its state-of-the-art nature as a historical climate reconstruction,

and Copernicus Land Cover for land cover variables due to its combination of high spatial and thematic resolution; and specific purpose designed mapping products for land change variables.

(2) For some drivers we selected the only available spatial product that was appropriate. The Biodiversity Intactness Index is the only globally comparable product that estimates local intactness of biodiversity as a function of location anthropogenic pressure; and the Global Relative Deprivation Index is the only globally comparable product that estimates relative social vulnerability.

(3) Certain drivers involved a decision to choose a specific product over other options. We selected the modelled hunting pressure index for tropical forests (Benitez Lopez et al. 2019) because it had a much larger geographic extent and more transparent modelling methodology than alternative, more geographically restricted, hunting pressure maps. We selected the specific landscape heterogeneity metric (second order dissimilarity from Tuanmu et al. 2015) because of both its globally comparable nature, and because the original study showed this metric was sensitive to anthropogenic landscape fragmentation and strongly correlated to biodiversity metrics (so a suitable proxy for the ecological dynamics of fragmented and ecotonal landscapes).

(4) Certain drivers were not feasible to match to gridded products so could not be empirically tested, for example wildlife trade.

(iii) 527-530: The authors' expertise spanned a wide range of disciplines, and while this certainly is beneficial for obtaining a wide range of perspectives, it also brings up the question whether authors' opinions should be weighted equally for determining potential disease drivers? I don't necessarily suggest that the authors alter their approach here, as any deviations might be arbitrary and only lead to small differences in the generated hypothesis sets, but I think it would still be worth commenting on why e.g., experts in "machine learning", "microbiology", and "public health" should be equally weighted for this exercise. And perhaps as an extension of that, I would appreciate if the authors could comment on the possible influence of confirmation bias in expert opinions on the selected drivers and, thus, the study's results.

Thank you for the request for clarification. As we have emphasised in our response to Reviewer 1 above, the majority of our author team have significant expertise in one or more specific diseases, with many joining the study to contribute outbreak data and attendant expertise. Our reference to disciplines and methods was intended to emphasise that our author team brings a wide array of disciplinary backgrounds to the study of these diseases (from molecular virology, to genomics, to ecology, to modelling, public health, etc). We have now ensured the disease specific expertise of our author team is more precisely described in the text (**lines 587-593**).

Regarding whether different authors' opinions should be weighted differently, our approach to this was to allow authors to opt in or out of completing the hypothesis exercise for each disease. So authors only completed the exercise for those diseases they felt they had sufficient expertise on, meaning that better known and/or studied diseases or those with multiple experts on the team (e.g. Ebola, dengue, influenza, hantaviruses, chikungunya, Zika) were completed by most authors, while those with fewer experts on the team (and often fewer experts worldwide, e.g. US arboviruses, melioidosis) were completed by fewer people. While this approach has its own limits, we felt this "opt in" approach was more transparent and fairer than

the lead authors attempting to retrospectively apply expertise weightings to each author. We have ensured this is clearly explained in the text (**lines 564-585**).

In response to the important concern about confirmation bias, we agree that changing the hypothesis selection method would only lead to small deviations in the tested sets of drivers and thus minimal changes in results. However this is important to examine in more depth. So, as we detail in our response to Reviewer 1, we have now expanded the analysis to test three sets of hypotheses under inclusion criteria ranging from very broad (retaining almost all disease-driver combinations) to strict (retaining only drivers that were top-ranked by authors). The new very broad "any author" criterion tests 97% of disease-driver combinations (77% after filtering out collinear variables and those with large amounts of missing data), so imposes minimal filtering based on author hypotheses. We find that the overall findings are robust between the "any author" criterion and the more stringent criteria, i.e. the patterns in the data are consistently recoverable (**Supp. Figures 2 and 3**). This strongly indicates that our conclusions are robust to potential influence of confirmation bias in driver ranking by our author team.

2. Correlations among covariates

Some of the chosen covariates (164-170, 570-588, Extended Data Table 2) are correlated by definition. This is, for example, pointed out by the authors for covariates related to land use change (687-689), and is also true for several others (e.g., per cent forest cover and per cent cropland cover). While the authors address the effects of these correlations in their discussions (e.g. Extended Data Figure 8), I am wondering whether it would be possible to formally account for such correlations in the analyses (e.g., by removing highly correlated variables) rather than just discussing their effects.

We did indeed formally account for these correlations in the analysis by removing highly correlated covariates; this process was described in **lines 632-636** (original manuscript) and **lines 683-691** (original manuscript):

"We examined collinearity among covariates via visual inspection, correlation matrix plots and variance inflation factors, and identified and excluded highly collinear covariates from multivariable models; this step was conducted manually rather than programmatically, to prioritize the inclusion of covariates with a stronger hypothesized relationship to each disease in question"

In the updated manuscript we have ensured this process is described as clearly as possible (**lines 716-722**).

Nonetheless, our analyses also indicate that correlation structure among these variables is not solely an analytic challenge, but is in fact an important structural feature of how the nexus of emerging disease risk differs in different locations. For this reason we have also moved the Extended Data figure on driver correlation networks to earlier in the paper (**Extended Data Fig 3**) and also conducted region-specific analyses for the disease-agnostic models (**Figure 2, Extended Data Fig 4**) and for two specific diseases (dengue and yellow fever; **Extended Data Fig 8**). We further detail these changes in our response to Reviewer 3.

3. Choice of buffer

477-482, 596-600 – Buffers were drawn "with a radius of 5km or another custom value". (i)

Could you provide a reason for this specific scale choice and how choosing differently might influence the results? (ii) How does variance in buffer values among datapoints influence the results? Could this introduce inconsistencies of how drivers were calculated and valued for different diseases or regions?

Drawing a buffer around outbreak point locations was designed to reflect the inherent uncertainty in the precise location where disease spillover events occur. The 5km buffer was chosen for point locations that did not have a specific buffer size provided (i.e. a custom value provided by the source datasets), to retain a relatively high spatial precision in matching covariates to outbreak locations. This approach makes the assumption that the spillover event occurred in relatively close proximity to the location where the outbreak was reported and geolocated (**lines 504-507**). To examine whether our findings could be substantially affected by this assumption - or by comparing covariates averaged across differently-sized geographic areas - we have now tested the correlation between mean covariate values calculated across buffers of 2km, 5km, 10km and 20km (i.e. covering the buffer range included in our dataset). We find a very strong correlation (all >0.8 and mostly > 0.95) between 5km and 20km radius buffers for all covariates, indicating that either choosing a different default buffer size or comparing across different buffer sizes is unlikely to substantially change our model results (**lines 521-526** and newly added **Supplementary Figure 4**). Thus, although aggregation of health data across variably-sized geographic units is an inherent challenge in the analysis of medical geographic data (Lee et al. 2016), the results of this sensitivity check suggest that this problem is unlikely to significantly impact our overall findings at this global and regional scale.

- Lee et al. 2016. Mind the Scales: Harnessing Spatial Big Data for Infectious Disease Surveillance and Inference. Journal of Infectious Diseases https://doi.org/10.1093/infdis/jiw344

4. Potential nonlinearities
111-113 – “In general, these studies suggest that habitat fragmentation ... increases wildlife disease prevalence30” However, reference 30 actually states that “the highest spillover risk occurs at intermediate levels of habitat loss, whereas the largest, but rarest, epidemics occur at extremes of land conversion”. Please rephrase and consider any consequences for the statistical modelling approach: in particular, what if the effects of drivers were not linear but e.g., hump-shaped with intermediate metric values providing the highest risk? Could that pose problems for the logistic regression analyses of the paper?

We agree this wasn’t the ideal reference to use for this specific point, so we have revised this sentence and changed the reference to a more appropriate paper focused on prevalence (Keesing & Ostfeld 2021; **line 111**).

We share your interest in the potential for nonlinear effects of certain key drivers, including the process of land conversion. However, as this referenced paper highlights, such effects will often manifest as complex and temporally dynamic changes in transmission within reservoir hosts, spillover risk, and outbreak risk (Faust et al. 2018). A growing body of recent literature, including some of our own studies, have shown that such complex nonlinear relationships can be inferred empirically, but that this requires high-quality data on disease incidence (i.e. transmission intensity) over both space and time (e.g. Carlson et al 2024; Gibb et al 2023; MacDonald & Mordecai 2019). Unfortunately such high-resolution data are unavailable for most high-concern emerging diseases (with a handful of notable exceptions e.g. Eby et al. 2022), and

this is reflected in our study's very small outbreak dataset sizes or lack of standardised case incidence data for the majority of diseases. As our focus was on applying a consistent, comparative framework across all diseases, this meant we were limited to testing linear driver effects (i.e. fixed effects).

With this issue in mind, our choice of covariates aimed to represent separate processes whose monotonic effects can combine to produce apparent nonlinear effects. For example, hump-shaped effects of land conversion on spillover risk are a product of interactions between rising levels of habitat loss and consequent rises in fragmentation - fragmentation tends to peak at intermediate stages of land change (Faust et al 2018). Our covariates represent these synergistic drivers via separate linear effects, so our framework should still capture much of this complexity even for more data-sparse diseases. We have explained this further in the Limitations section of the Methods (**lines 897-900**).

- Keesing & Ostfeld, 2021. Impacts of biodiversity and biodiversity loss on zoonotic diseases. PNAS. https://doi.org/10.1073/pnas.2023540118
- Faust et al. 2018. Pathogen spillover during land conversion. Ecology Letters https://doi.org/10.1111/ele.12904
- Carlson et al. 2024. The historical fingerprint and future impact of climate change on childhood malaria in Africa. medRxiv https://doi.org/10.1101/2023.07.16.23292713
- Gibb et al. 2023. Interactions between climate change, urban infrastructure and mobility are driving dengue emergence in Vietnam. Nature Communications. https://doi.org/10.1038/s41467-023-43954-0
- MacDonald & Mordecai 2019. Amazon deforestation drives malaria transmission, and malaria burden reduces forest clearing. PNAS https://doi.org/10.1073/pnas.1905315116

5. Implications of some diseases being much more strongly represented in the data than others
The most sampled diseases (Lyme, Dengue, West Nile) seem to be making up more than half of the data, and as such, seem to be driving the results of the global analysis shown in Figure 2. These models are e.g. referred to by the authors as "disease-agnostic", but given this imbalance in sample sizes, I wonder whether they indeed are "disease-agnostic" or whether they just represent the results for the most commonly sampled diseases. Contrasting the effect sizes shown for individual diseases (Fig. 3) vs those of the global model (Fig. 2) might provide more insight into this. Alternatively, I wonder whether it would be insightful to rerun the analyses while subsampling each disease to provide more comparable sample sizes?

Thank you for this insightful suggestion, and we agree that the unbalanced representation of data from across diseases in the global models could have impacted the results. Therefore, we have redesigned the disease-agnostic modelling approach to balance sample sizes across all diseases, while capturing uncertainty using an ensemble bootstrapping approach. To achieve this, we estimated the posterior fixed effects distributions for each driver across an ensemble of 100 submodels, each of which was fitted to a randomly-drawn subsample of the data with balanced sample sizes across diseases (100 points per disease, or all points for diseases where $n < 100$). Credible intervals were calculated across posterior samples drawn from all 100 submodels, ensuring that the fixed effects estimates and associated uncertainty reflect a balanced sample of all diseases. We have updated **Figure 2** and associated supplementary figure (**Extended Data Figure 4**) to reflect this new approach, showing the posterior median, 67% and 95% credible intervals, and we describe this method fully in the main text (**lines 192-**

196) and Methods (**lines 746-759**). The results of this new method were generally consistent with the findings from the previous version of the models, with the strongest effects of detection covariates, fragmentation, forest cover and livestock density. However, the uncertainty intervals were wider, leading to weaker evidence for overall effects of biodiversity intactness or precipitation drying across all diseases, and further emphasising the importance of the disease-specific approach we take in the later analyses.

We also agree with your suggestion that it would be insightful to more explicitly compare overall effects derived from the global model (**Figure 2**), from those derived from disease-specific models (**Figure 3**). Therefore, we have added a new analysis to the study which takes a "bottom-up" meta-analytic approach to estimate the average effect of drivers across the individual disease models (**Figure 4a-c**). For each driver, we calculate the posterior distribution of the mean marginal effect size across all individual disease models: for each driver, we use posterior samples from each disease model to generate a posterior distribution of the mean effect size across all tested diseases, which is summarised to median, 67% and 95% credible intervals. Importantly, this approach explicitly integrates variability in credible interval width between diseases (due to variation in sample sizes) while still making full use of the full dataset for each disease (**lines 802-824**).

In parallel with the driver sharing networks, we conduct this meta-analysis including all 31 diseases, then for subsets including either vector-borne or directly-transmitted zoonoses (**Figure 4a-c**). This updated synthesis figure provides further clarity of the variation in effect sizes by transmission pathway. In particular, we find evidence that, for vector-borne zoonoses, forest cover and landscape heterogeneity drive large increases in outbreak risk, as do long-term declining rainfall trends (**Figure 4b**), whereas we find little evidence for net positive or negative driver effects for directly-transmitted zoonoses (except biodiversity intactness; **Figure 4c**). This addition of mean effect sizes complements the driver sharing network analysis (**Figure 4d-f**), providing strong evidence that multiple vector-borne zoonotic risks cluster at the nexus of fragmented forests, cities, and areas experiencing long term declines in precipitation. We discuss these updated results in the text in **lines 322-366**.

6. Focus on effect size
211-219, Fig. 2c – (i) Given the number of datapoints, and in particular the number of datapoints from a well-sampled diseases, it is perhaps not surprising that effects of social/ecological risk factors were detected and that these aligned with current consensus on likely risk drivers (219-222). However, effect sizes are small for some drivers, as also noted by the authors themselves, so I would recommend discussing these results not just in terms of detectability but also in terms of effect sizes, as well as discuss to what degree these results could be driven by the overrepresentation of some diseases (cf. also point 6 above).

We agree on the importance of effect sizes, and have focused further on these in three ways.

Firstly, we have redesigned **Figure 3** to be visually clearer in the size, direction and significance of individual disease effects, by removing uncertainty intervals from the visualisation of fixed effects (as these were difficult to see clearly in the previous version). Uncertainty intervals are still included in **Supp Figure 1** and propagated into the meta-analysis of effect sizes (**Figure 4**). The updated **Figure 3** makes the relative strength and heterogeneity of driver effects across diseases much clearer.

Secondly, we have conducted the meta-analysis of mean effect sizes to complement the driver sharing networks (**Figure 4a-c**). These show more clearly the variation in the size of different driver effects across diseases (see our response to the previous comment).

Thirdly, we have ensured effect sizes are discussed in relevant places in the text in relation to all the sub-analyses in the study, for example:

- "At a more local scale, outbreak events are much more commonly reported in cities and near clinics, with these two effect sizes much larger than any other covariate effects" (**lines 206-208**)
- "Social and ecological risk factors had substantially modified effect sizes and wider uncertainty after adjusting for detection and reporting processes" (**lines 212-213)**
- "common and almost always positive effects of landscape heterogeneity (15 out of 30 tested) and forest cover (13 out of 27 tested), that were comparable in effect sizes to detection drivers" (**lines 259-261**)
- "impacts of recent cumulative forest loss (2000-2020) in relatively few systems for which this driver was tested (6 out of 29), with a mix of directional effects (two positive, four negative) and mostly small effect sizes" (**lines 270-273**)
- "[Vector-borne zoonoses] are more strongly and consistently sensitive to ecosystem drivers, with large mean effect sizes (Figure 4b) and a much higher rate of driver sharing between diseases (Figure 4e)." (**lines 345-347**)

Minor Comments

161 – A few references are from preprint servers, such as bioRxiv. I will defer to the editor regarding the journal's policy regarding the use of such references but think that at least some of these might be replaceable by fully peer-reviewed sources.

All preprints that were cited have now been published in peer-reviewed form, and we have replaced the citations with their full published versions.

178-181 – "including continuous geospatial random intercepts": although I understand what the authors mean, I think it would still be beneficial to provide the non-specialized reader with a one-sentence intuitive explanation of what this term does in the analyses.

To ensure this is clear for a nonspecialist reader, we have expanded this to the following: *"All models included a continuous spatially-structured random intercept, to account for macro-scale unexplained variation in both outbreak risk and data availability (for example due to reporting biases across countries or regions; Methods, Extended Data Figure 2)."* **(lines 180-182)**

213-216 – Some of the found effects seem to be contradicting one another, e.g. higher risk with higher livestock density as well as with higher biodiversity intactness and protected area coverage. I think this would deserve additional discussion, please also see my comments above about covariate correlations.

It is important to highlight that these inferred fixed effects are marginal effects, i.e. they describe the effect on risk associated with changing one covariate while holding all others constant at a specific level. Consequently, these findings are not necessarily contradictory; in

the given example, increasing livestock density would increase risk at any given level of biodiversity intactness, etc. We have made efforts to emphasise the marginal nature of these effects in the text and figure legends (e.g. **lines 193-196, 254, 984-990, 1022**).

After following your earlier suggestion to subsample the data to ensure balanced representation across diseases, we also find that these seemingly conflicting findings are largely absent - i.e. the results are more consistent and clear. Importantly, the global model results (**Figure 2**) reflect the aggregate effects across all diseases; when we fit individual models per disease, we find that such apparently conflicting results are mostly absent (**Figures 3-4, Extended Data 6).** This further reinforces the importance of moving beyond the overall modelling approach of Figure 2 towards disease specific inference and synthesis.

379-381 – I agree with the statement that improving surveillance would help address data gaps, but I wonder if the authors could provide more specifics regarding what is missing the most / would be the most valuable to collect in the context of their study, so to guide such surveillance efforts.

We agree this merits further elaboration. In terms of addressing the data gaps in our study the key gaps to close are around improving the spatial and temporal granularity of disease data, and better disentangling true infection and disease rates from reporting bias. Therefore we have added a clause to highlight this: "for example, paired syndromic and serological surveillance across networks of sentinel sites to better track spillover and disease rates in people and animals" (**line 398-400**). Members of our team also recently published a paper highlighting the benefits of taking a combined approach to biodiversity monitoring and disease surveillance, which also contains insights into this question beyond what we have space for in this manuscript (Poisot et al. 2025). We have therefore also added a citation to this paper.

- Poisot et al. 2025, "Biodiversity science and biosurveillance are fellow travellers", BioScience. https://doi.org/10.1093/biosci/biaf091

387 – The notion of "wicked problem" may be familiar to some, but I suspect not to everyone. I suggest a reference or rewording.

We have added a reference to a foundational paper on wicked problems to define this (Rittel & Horst 1973) (**line 408**).

460 – Not clear what you mean by "administrative level 1 or 2". Please define.

We have rephrased this to clarify, as "first or second-level subnational administrative divisions" (**line 485-486**). Hopefully this is now clearer.

535-536 – "but we do not recommend this as a template for a rapid, large-scale survey exercise" I'm not sure I see the relevance of this statement and suggest deleting.

We have modified this to be more concise, but have kept the meaning of this statement intact. We feel that, especially considering the wider set of concerns from reviewers, it is important that we clearly differentiate this template as a specific tool designed for coauthors to participate in hypothesis generation, rather than a widely-applicable survey exercise. The updated text now states the following: *"While this online exercise was a concise approach to*

participatory hypothesis development across an international author team, authors still reported spending multiple hours (>2) to fully complete the matrix, so we would not recommend this design as the basis for a large-scale survey." (**lines 596-599**).

606-613 – Please clarify the procedure regarding how the number of background points were chosen, and why this was exactly "between 2 and 8". The sentence "Guidelines on..." seems to suggest that while guidelines exist, something else might have been done here, so this leaves me confused regarding the correctness and meaning of these specific numbers.

Our note about "guidelines" was specifically in relation to predictive species distribution modelling (SDM) studies. The selection of background pseudoabsence points has been extensively tested in the SDM literature - this literature has generally shown that there is no one "correct" approach, and that results tend to be relatively similar across different approaches.

However, SDM studies are focused on optimising for predictive mapping rather than inference, and as such, have a different analytic objective than we do in this study, so are not applicable. As we stated in the original Methods: *"we note that this is a distinct statistical approach; our objective here is to detect predictor effects, rather than correctly model the area of occupancy"* (**original MS, lines 611-612**).

Our priority instead was ensuring statistical power and sufficient coverage of the background area (i.e. an adequate number of background points), while balancing against computational modelling costs. The choice of between 2 and 8 times as many background points was made with this in mind, and for each disease, was informed by the sample size and the geographical dispersion and clustering of points. For example, diseases like Oropouche and Ebola cover a very wide geographic area (entire tropical forest regions) but have relatively few outbreak points that are geographically sparse; diseases like these required a higher multiple of background points to ensure that the study area was fully captured. In contrast, diseases like Lyme and West Nile have a very large number of outbreak points that cover much of the geographic area, so selecting just twice as many background points was sufficient.

We have ensured the description of this approach and rationale are more clearly explained in the updated manuscript (**lines 690-698**).

Fig 1a – Some of the colors on the map do not seem to match the color code below it. E.g., most of the U.S. seems to have dark blue dots, but there is no dark blue color in the code. Could this be due to dots with different colors overlapping? If so, it might be helpful to provide additional supplementary maps that do not have this issue, e.g., by separating commonly overlapping diseases

The apparent colour coding issue is a consequence of the overlaying of translucent points of the same colour, especially for diseases with multiple outbreaks in the same location across multiple years. The dark blue points, for example, are for West Nile. We have fixed the legend translucency to make the colour-to-colour mapping visually clearer, but also emphasise that Figure 1 is designed mainly to show the overall geographic distribution and diversity of the data, rather than precisely mapping the points for each disease.

Extended Data Tables 1 and 2: Not clear what the color code in the last column is referring to. Please define.
These colour codes refer, respectively, to major transmission pathway to humans (Extended

Data Table 1, aligned with the colour code in Figure 1), and driver type (Extended Data Table 2, aligned with the colour code used throughout the results figures). We have added text to the table legends in the Extended Data to clarify this.

Referee #2 (Remarks on code availability):

Please note: I have reviewed all aspects of the manuscript, except for the code. The reviewer instructions specified that:

>> It would be most helpful if you could take a look at the code provided by the authors and comment on whether it provides sufficiently clear information and documentation that would make it possible to test the code and replicate the paper's findings. We would also be grateful if you, or someone in your group, could look at the code more closely and verify that it can indeed be run.

but, while all code seems accessible and clearly organized, I typically do not program my own work in R, and as such am not well-suited for reviewing their code. I hope one of the other reviewers was able to provide this expertise.

We have now updated the code and data base on the repository, which contains all the disease data that we are able to share (i.e. are already in the public domain). To demonstrate that the modelling pipeline is reproducible and ensure it could be used by other researchers, we have now developed a reproducible example subset of the code, which can be quickly run by any user to generate results for three exemplar diseases. This is available at https://github.com/viralemergence/fingerprint-preprint/ and will be archived as a permanent Zenodo repository (with DOI) once the manuscript is accepted and finalised for publication.

Referee #3 (Remarks to the Author):

Summary:

In this paper, the authors study the socio-environmental drivers of disease outbreaks. Global data are gathered for several diseases which are analyzed using a spatial statistical model. The dataset and analysis are impressive and the results may be useful for policy development. The authors' attention to detection bias and the clear and fair discussion of the associated limitations of what can and cannot be inferred (i.e., whether distance to a health care facility affects detection, outbreaks or both) from the analysis is laudable. Overall, I feel this paper is a nice contribution, but I do have a few comments.

Thank you for your positive comments and the suggestions to improve the clarity, breadth and description of our analyses.

Major comments:

(1) The assumption that the same model applies across time and continent is very strong and should be tested. You could for example fit the model separately by continent and determine if the covariates effects are similar across fits.

We agree this is a good idea, especially in light of our finding that the nexus of emerging disease drivers, and their correlations, are distinctly different between different global regions (networks in **Extended Data Figure 3**). We have addressed this via two sets of updated analyses.

Firstly, we fitted subsets of the global multi-disease model (**Figure 2**) for different major geographic regions with sufficient outbreak data coverage in our dataset (North America, South Asia, East Asia & Pacific, Latin America & Caribbean, Sub-Saharan Africa), which are shown in the new **Extended Data Figure 4b** and discussed in **lines 219-228** of the manuscript. This has revealed that the results from the global model (**Figure 2**) are broadly similar across regions of the globe - and most consistently for the detection-based drivers, forest cover and landscape fragmentation - but with some clear differences. Most notably, the strong influence of livestock density is mainly restricted to South Asia and East Asia & Pacific, with no overall effect in regions outside Asia (**Extended Data Figure 4b**). This breakdown by region highlights that the different driver nexus in different regions matters for emerging disease risk, and it also further reinforces that, to understand why these differences occur, we need the disease-specific approach we take with the rest of the manuscript **(lines 225-228)**.

Secondly, we also fitted region-specific models for the two specific diseases with sufficient representation of data from more than one region: dengue (Latin America & Caribbean, Africa, Asia & Pacific), and yellow fever (Latin America, Africa) (**lines 800-803**). The aim of this analysis was to test whether individual diseases show consistency in their inferred drivers across different regional contexts, and especially whether drivers were substantially different for yellow fever between the Americas and Africa, given documented differences in this disease's current epidemiological characteristics (Possas et al. 2017). The results are shown in the new **Extended Data Figure 8** and discussed in **lines 308-309** of the manuscript. Overall we find a high consistency in drivers of dengue across the three major regions, particularly for detection and ecosystem structure drivers, and in particular a consistent effect of precipitation drying across all three regions - a notable result given the growing evidence for the important role of drought in dengue dynamics (Gibb et al. 2023, Lowe et al. 2018). Yellow fever shows a similar overall set of drivers across regions, but with a notably different influence of urban areas, which have a strongly positive effect on risk in Africa but not the Americas - a finding that potentially reflects differences in detection bias, but is also consistent with the lack of documented urban transmission cycles for yellow fever in the Americas.

- Possas et al. 2017. Urgent call for action: avoiding spread and re-urbanisation of yellow fever in Brazil. Mem. Inst. Oswaldo Cruz. https://doi.org/10.1590/0074-02760170361
- Gibb et al. 2023. Interactions between climate change, urban infrastructure and mobility are driving dengue emergence in Vietnam. Nature Communications. https://doi.org/10.1038/s41467-023-43954-0
- Lowe et al. 2018. Nonlinear and delayed impacts of climate on dengue risk in Barbados: A modelling study. PLoS Medicine https://doi.org/10.1371/journal.pmed.1002613

(2) The use of the survey to select covariates is unconventional so I feel it should be further justified and explained. First, I did not see a clear statement that the survey was conducted before the survey participants had looked at the data or preliminary results. Also, the selection process to form the panel should be make more explicit. Finally, the process of gathering information from other researchers is a common way to select variables, but it is also not clear to me why the survey of 25 experts is preferred over a systematic literature review.

Thank you for the request for clarification. As discussed in previous responses, we feel it is important to emphasise that the hypothesis generation approach we took was not a survey, and we were careful not to use this language in the original manuscript. We designed this exercise as a way for a geographically widespread group of coauthors to participate in hypothesis generation, which was preferable to making this the responsibility of a single lead analyst. We have provided a thorough explanation and justification of the approach in our responses to earlier reviewers, but to summarise in response to the specific concerns raised in this review:

A systematic literature review could be an alternative way to approach hypothesis development, although the scientific literature on emerging disease drivers is itself extremely large, variable in quality, and shaped by complex geographical and socioeconomic biases, as well as disciplinary conventions (Gibb et al. 2022, Wilkinson 2016, Jephcott 2024). Indeed, a recent systematic review of studies of environmental drivers of zoonotic and vector-borne diseases showed that, of 312 eligible studies, 52% solely tested ecological drivers thought to shape hazards, with only 7.4% also testing for social influences on exposure and vulnerability (Carvalho et al. 2025). It is therefore not guaranteed that a systematic review of the literature would provide a higher-quality set of hypotheses to test than our approach, which combines the expertise of an author team with extensive subject-specific on the ecology, transmission routes and dynamics of emerging infections, members of which have published on the majority of diseases included in this study. We have emphasised this in the text (**lines 564-599).**

Nonetheless, we acknowledge that our hypotheses will, to some degree, still be influenced by the biases of our author team. To ensure this does not systematically bias our study findings, we conducted an additional sensitivity test using hypotheses generated using a minimal stringency criterion that tests all but highly implausible drivers. We find that this approach, which allows the patterns in the data (rather than the hypotheses) to dominate, leads to the same overall findings as the more focused hypothesis sets. This has improved our confidence that the results would not substantively differ under other approaches to hypothesis generation (**Supp. Figures 2 and 3)**.

Finally, thank you for highlighting the important question of whether the coauthors had seen the data or results before completing the hypothesis exercise. We have now confirmed in the Methods that the coauthors had not seen the combined data or preliminary results. The lead analysts (RG, CJC) had seen preliminary results for three diseases during model pipeline development (Lassa fever, Ebola and CCHF), but not the final multivariable model results after adjustment with geospatial effect and detection covariates. Several disease datasets were contributed by authors who had familiarity with their own datasets, but not with our study covariates, data processing, model design or full multi-disease dataset. These details are now included in the manuscript at **lines 582-585**.

- Gibb et al. 2022. Mammal virus diversity estimates are unstable due to accelerating discovery effort. *Biology Letters* https://doi.org/10.1098/rsbl.2021.0427
- Wilkinson, 2016. Emerging Disease or Emerging Diagnosis? Lassa Fever and Ebola in Sierra Leone. *Anthropological Quarterly*. https://primo.qatar-weill.cornell.edu/permalink/974WCMCIQ_INST/1e7q4lh/cdi_proquest_journals_1940161704
- Jephcott, 2024. Propagating Visions of a Forest Reservoir: A Supposed Zoonotic Outbreak in the Brong-Ahafo Region of Ghana. *Medical Anthropology* https://doi.org/10.1080/01459740.2023.2166411

- Carvalho et al. 2025. Unpacking the risks of zoonotic and vector-borne pathogen transmission to humans in the context of environmental change. *One Earth* https://www.sciencedirect.com/science/article/pii/S2590332225001745

Minor comments:

(3) Towards the end of the Introduction you say that "random intercepts were included to account for unexplained macro-scale patterns of data availability", but the random intercepts could also capture unexplained variation in the true likelihood of an outbreak.
This is a good point and we have changed the text to reflect this (**lines 180-182**).

(4) In the second paragraph of the "Shared drivers..." section, you discuss "interactions", but actually I think "co-occurrence" in the model as used later is more appropriate. Interaction usually means that the product of covariates is included so that the effect of one can be modified by the other.
We agree this could be more clearly phrased, so we have changed the terminology of this section to focus consistently on "driver co-occurrence" and "shared drivers" (**line 320-366**).

(5) In the "Statistical Modeling" section, tuning the spatial covariance parameters "via visual inspection" is too informal. I would at least be more descriptive about what you were looking for, and preferably this would be replaced a more rigorous approach such as WAIC comparisons. Also, I would call WAIC a "model comparison metric" rather than a "model adequacy metric" since it cannot be used to conclude any model is adequate, other than it fits better than other candidates.
We agree the spatial field fitting was not explained in sufficient depth. We had also erroneously stated that we tuned the hyperparameters, but in fact we adjusted the hyperpriors; posterior distributions for each hyperparameter were inferred during the model fitting process. Different diseases had data from across very different sized geographic areas (from a small subnational area for Argentine haemorrhagic fever; to global and multi-continent for dengue), so the spatial range and variance of the fitted field needed to vary significantly. Assigning hyperpriors that were too small or too large for any given disease would sometimes result in either over- or underfitting of the spatial field, i.e. either extreme shrinkage to each point, or capturing very little spatial structure. We set the hyperpriors for each disease to ensure that each model fitted with a visually smooth spatial field across the outbreak data points (**Extended Data Figure 2**). Despite their penalisation of model complexity, WAIC and other information criteria can still favour overfitted spatial models as the "best" model, so it was inappropriate to use these for hyperprior adjustment for the spatial field. We have explained this more clearly in the Methods (**lines 736-740**).

Second, we agree that WAIC is best described as a model comparison metric and have changed the text accordingly (**line 742**).

Response to referees for Gibb et al. "The anthropogenic fingerprint on emerging infectious diseases", April 2026

Our thanks to the reviewers for their positive comments, and to the editors for the opportunity to resubmit the manuscript. We have now revised the manuscript to address Reviewer 2's comments about ensuring our results are findings are clearly described and explained (please see below for line-by-line responses). As recommended in the editors' email, we have also line-edited the abstract and main text to slightly reduce the overall length, ensure all descriptions are concise, and move any unnecessary technical detail into the Methods.

Referees' comments:

Referee #1 (Remarks to the Author):

I thank the authors for addressing each of my primary concerns, and commend them on the new analysis, figures, and revised text that was responsive to each of the reviewers' concerns. I have no further suggested changes.

Referee #2 (Remarks to the Author):

Thank you to the authors for carefully responding to all my previous queries. The additional clarifications and analyses are tremendously helpful and address all my previous concerns. I especially appreciate the implementation of the new subsampling strategy for the disease-agnostic model and think that this has substantially improved the robustness of the analyses. I also appreciate the new analyses regarding the sensitivity of their results to buffer size assumptions, which again help with demonstrating robustness. Furthermore, I think that the additional details and analyses regarding the hypothesis generation exercise are very helpful, and in particular, I agree with the authors that the inclusion of the "Any author" rule further demonstrates robustness of their results. The new meta-analysis of driver effect sizes, along with the regional analyses, are insightful and help to further tease apart key patterns.

Overall, I think this is an impressive and insightful study that sheds new light on how environmental and social covariates drive global disease outbreak risks, using an extensive global dataset. The analyses are well-structured, thorough, and impressive in their scope. The methods and conclusions seem robust, and uncertainties and limitations are well-identified and discussed. The article is well-written and the analyses are, despite their complexity, easy to follow. I only have a few minor suggestions that I list below:

Thank you, we are glad you appreciated the revisions to the manuscript, and appreciate your further suggestions to improve the clarity of the text and findings. We have addressed these as follows.

Line 72: "adjusting for" -> I suggest "accounting for"

75: “as well as” -> “along with”

We have made these text corrections.

75-76, 262-265, 308-309: In each of these places, the authors discuss the effects of climate drying on the outbreak risks of dengue and some other diseases (e.g., “This combination of factors, as well as long-term declining trends in precipitation, share strong impacts across multiple vector-borne diseases (e.g. dengue, West Nile and Lyme disease”). Figure 3 and Extended Data Figure 6 suggest general trends, as stated by the authors (262-265), but I do think it would be important to provide additional discussion on the general direction of effects, exceptions to these trends, and possible mechanistic explanations. I suggest this because, naively, one might assume that more precipitation would result in more standing water, and therefore more mosquito habitat and more disease – but it seems you’re seeing the opposite effect. I wonder whether considering why some diseases show opposing trends than others (most notably West Nile, Supp. Fig. 1) could be informative in explaining these observations.

We agree it would be insightful to add further detail on potential mechanisms underlying the climate drying effect. Overall, it is likely that the precise factors that underpin this differ between diseases, but there is growing evidence that long-term changes in precipitation dynamics, and the increasing incidence of sudden “whiplash” events between extreme drought and rainfall (Swain et al., 2025, Nat Rev. Earth & Environment), can be linked with severe outbreaks for a number of mosquito-borne and water-borne pathogens.

The effect of drought seems counterintuitive given the need for standing water for mosquito breeding, but other studies have indicated that this can be driven by several mechanisms related to mosquito ecology and human-mosquito contact, including water storage around homes during drought for dengue and other Aedes-borne infections (creating domestic breeding sites; Gibb et al. 2023, Nat Comms), or increasing vulnerability of landscapes to flooding after periods of drought (increasing abundance of floodwater mosquitoes that transmit Rift Valley fever; Rostal et al. 2025 Lancet P. H.). Recent evidence has, in contrast, suggested that the effects of drought on West Nile in North America appear particularly complex, with drought typically suppressing mosquito abundance (Sambado et al. 2025, Proceedings B); this might contribute to its diverging effect in our study.

Our findings are interesting in suggesting that long-term drying might, in general, be increasing the susceptibility of landscapes to more dynamic outbreak triggers (such as extreme rainfall), across quite a large set of diseases; as well as showing that, for dengue, this increased risk spans all three continents where the disease is a major health problem (Asia, Africa, Americas; Extended Data Figure 8). We have added some additional detail to the main text to discuss this, as follows (lines 259-267).

“For a quarter of diseases (8 out of 31 tested), outbreak risk was strongly linked to long-term changes in annual precipitation. Notably, areas experiencing climate drying trends are at higher risk of outbreaks of several vector- and water-borne diseases with known or suspected links to hydrometeorological anomalies (e.g. Rift Valley fever53, dengue54, melioidosis55 and Japanese encephalitis56; Extended Data Figure 6). This indicates that long-term drying may be increasing underlying societal or landscape susceptibility to the dynamic factors that trigger outbreaks. For example, abrupt transitions between long-term drought and extreme rainfall are increasingly implicated in explosive arboviral outbreaks, even though the precise mechanisms vary by disease (e.g. domestic water storage for dengue54, versus floodwater mosquito breeding for RVF53).”

Also, please clarify in Extended Data Table 2 how “Precipitation Change” is defined. Precipitation in 2000-2020 minus that in 1950-1970, or vice versa? The direction is not clear from the descriptions in the second and fifth columns.

We have changed this to be precise about the directionality and meaning of the covariates for both precipitation change and temperature change, as follows:

“Difference in mean annual precipitation between present day (2000-20) and historical reference period (1950-70) (positive values denote an upward trend in precipitation since the historical reference, and negative values denote a drying trend)”

“Difference in mean annual air temperature between present day (2000-20) and historical reference period (1950-70) (positive values denote a warming trend since the historical reference, and negative values denote a cooling trend)”

121-127: Here, you discuss previous studies that have examined “ecological correlates of human disease emergence”, summarize their findings (“These studies have found that land use change, agricultural expansion, biodiversity hotspots and global travel are widely associated with observed geographic hotspots of disease emergence, 123-125), and outline how their approach differs from yours (125-135). I understand space restrictions might be limiting, but if possible, I think it would be interesting to refer back to this after you have presented your results (e.g., in your Conclusions, 404-427) and briefly discuss whether / how the ecological correlates in these two contexts (previous studies on emergence vs your study on ongoing risk) are similar or not.

Thank you for this suggestion and agree it is important to take the opportunity to set our evidence in the context of previous global studies. We have added some additional sentences to the final “Conclusions” section, as follows (lines 403-413).

“This trend may share common causes with both the climate and biodiversity crisis, but is also a “wicked problem”78 in its own right: our findings suggest that emerging infectious disease risks are ubiquitous, and widely associated with mosaic landscapes where people and cities live alongside forests and fragmented

ecosystems. ***Our approach has substantially expanded on earlier global studies by examining comprehensive outbreak geographies (rather than solely points of first emergence), testing disease-specific socio-ecological hypotheses, and adjusting for data biases at multiple scales. This has provided a more nuanced picture of how anthropogenic impacts vary across the diversity of emerging human infections.*** We find evidence of a strong and consistent anthropogenic fingerprint on vector-borne diseases, suggesting that over the coming decades, risk is likely to increase unless there are substantial shifts in human activities. […]"

131: The sentence "Assigning outbreak drivers solely based on expert opinion…" does not seem to follow logically from the previous sentences (which talk about the limitations of emergence data) -> reword to clarify link.

We agree the logical flow of this paragraph could have been clearer. We have reframed this paragraph to explain more clearly that, although, there are specific studies that have assigned drivers based on expert opinion, these are difficult to generalise to wider patterns of risk, as we do in our study (lines 132-144).

"Several influential studies have shown that geographic hotspots of human disease emergence events (i.e, the location and circumstances of the first scientifically documented outbreaks of around 300 pathogens) correlate with ecological factors including land use change, agricultural expansion, biodiversity hotspots and global travel3,5,29,30. Other research has identified similar drivers of historically significant outbreaks via literature review or expert opinion5,29. However, the circumstances of the first confirmed human infections, or of unusually large outbreaks, may not accurately reflect the social and ecological factors that determine wider landscapes of infection risk. For example, data on emergence events are inherently biased towards better-resourced settings where novel pathogens are more easily identified, and away from rural and marginalised populations that experience an endemic burden of zoonotic and vector-borne diseases31 (including many infections typically framed as "emerging"32). The growing availability of comprehensive georeferenced outbreak datasets for many of these diseases – compiled from disease surveillance reports, scientific and grey literature33 – now provides the opportunity for a more systematic, global, data-driven assessment of emerging disease drivers."

259: "Outbreak risk was higher in fragmented and forested landscapes […], with common and almost always positive effects of landscape heterogeneity." Here, and in a few other places (e.g., 275, 305, 310, 571), I noticed that I had to stop and remind myself what was meant by "positive". While in most places the manuscript is unambiguous (e.g., "more heterogeneity means higher risk"), there are some places where I wondered whether general readers could misinterpret such statements in a colloquial sense ("heterogeneity brings positive impacts, i.e., lower risk"). In Supplementary Table 2, you actually state that "Make sure to think carefully about what sign means for the direction of each variable; for example…", and I suggest that a similar clarification could be helpful to have in the main text at an appropriate

place (along with a careful review of all places where the words “positive” and “negative” are used to ensure that statements cannot be misunderstood).

We agree the use of “positive” and “negative” is potentially open to misinterpretation, so we have ensured we avoid using these terms throughout the main text, and for clarity, have changed most sentences to refer to “increases in risk” or “decreases in risk” (e.g. lines 269-286, 331-334).

895: “Borrelia burgdorferi or West Nile fever” -> I suggest “Lyme or West Nile fever” (i.e., using either the name of the disease or the name of the causative agents consistently would help avoid confusion)

1145: “diseases names” -> “disease names”

1154: “author” -> “authors”

1171-1172: “diseases are ordered from left to right by number of outbreak records (lowest to highest)” -> I think this should be “highest to lowest” (Lyme is on the left, Marburg is on the right)

1410 & 1416: “B-F” should be “B-G”

We have made these suggested text corrections.

Referee #3 (Remarks to the Author):

Thank you for the thoroughly responsing to my comments. I am satisfied with the reviewed manuscript.

Referee #3 (Remarks on code availability):

I downloaded the data and code from github and run the small example on my laptop. The data and code are in order and the example worked fine.

Response to editorial requests for Gibb et al. "The anthropogenic fingerprint on emerging infectious diseases"

Thank you for in principle accepting our manuscript for publication. We believe we have now addressed all the concerns and issues raised by the editors to assist in the editorial process, and we have provided a full breakdown of these changes below. There were no additional changes or edits requested by the reviewers.

1. More information is needed in the data availability statement, which is ambiguous. What disease data currently can't be shared? Please see our guidelines for data availability and the information that needs to be provided regarding restrictions on data access: https://www.nature.com/nature-portfolio/editorial-policies/reporting-standards.
Please also separate the code and data availability statements and deposit data and code on zenodo or similar doi-minting repository.

We have now clarified this by providing a full Data Availability statement, separate from the Code Availability statement, which explains precisely which data sources are shared within the study repository. Most datasets are public domain already so processed versions of these data, sufficient to reproduce the findings, are included in the repository. A smaller number of diseases cannot be shared because we do not hold the rights to share them onwards; in these places we have provided full descriptions in Supp. Table 1 of how to access them, either via URLs to online surveillance portals, or providing email addresses for data providers who will make data available on reasonable request.

2. Please reduce the Abstract to 230 words or less. Currently there are 252 words.

We have now reduced the abstract to 230 words.

3. The number of references should generally not exceed 60. Currently you have 93 references.

We have now restructured the references so that the Main Text now only cites 59 items, and have removed any extraneous citations. The overall reference list still contains 91 items, because we needed to cite the source publications for all the disease datasets and covariate datasets (wherever possible), as well as key methodological references for the statistical modelling. These make up most of the additional references within the Methods. Given the importance of crediting data sources for a large synthesis project like this, we would like to request if it is possible to make an exception to the usual 60 reference limit to allow for this.

4. Please create a separate reference list for any methods references, making sure that the numbering continues from the main text references.

We have now done this (see above).

5. Please re-supply the figures in an acceptable format such as EPS, AI, PS, PDF, PPT, CDR, PSD or XLS (for graphs)

We have provided editable format versions of the main text figures in .SVG format.

6. You have more than one account on EJP. Please contact Nature Manuscripts (naturemanuscripts@nature.com) to confirm all accounts which belong to you, and your primary email address.

We have contacted Nature Manuscripts to resolve this issue, although have not heard back as yet.

7. Please provide a supplementary information guide as a separate word document.

We have provided this.

8. There are potential third party rights issues in the figures. Please check the sources of all illustrations and clarify whether permissions are needed to adapt or reproduce them. Please make sure to include the relevant details in third party rights table when you resubmit (more information below). If Biorender or a similar software has been used, please also ensure to provide relevant licenses. In particular please check figure/s: main figure 1a, 2d.

The maps that we used to generate the figures were from Natural Earth data, and are fully public domain and able to be used without credit. However, for transparency, we have now ensured that the figure legends credit the source as Natural Earth (https://www.naturalearthdata.com/). We confirm that there are no display items that present issues for third party rights, so we have not filled in a third party rights table.

9. Please ensure that the text size in all figures is at least 5 pt Arial.

We have done this.

10. Please remove the Extended data figures from the article file and re-supply them individually in EPS, JPEG or TIF format. The ED tables should be converted to SI Tables if they are too large to fit on a single printed page.

We have removed the Extended Data figures and tables and provided them separately. The tables contain information that is useful for quick reference while reading and interpreting the manuscript, i.e. details about each disease and covariate layer, so if possible we strongly request that these be retained within the Extended Data with the fonts scaled to an appropriate size to enable them to be printed on a single page. We are happy, if needed during the editorial and proofing process, to explore reducing the information content in these tables to ensure they can still be provided as part of the Extended Data.

11. Funding information is provided along the acknowledgment section, please provide it as separate section.

We have done this.

12. Please see the attachments for further information about revising your paper and Reporting Summary.

We have addressed the issues raised in the attachments around ensuring that figure legends properly describe the number of models or observations that go into each result (Figure 5 and ED Figures 4 and 8). We have also revised the Reporting Summary to address the issues raised in the attached. We have also completed the rest of the Nature check list items, which are attached to the submission.
