# Supplementary material for: The anthropogenic fingerprint on emerging infectious diseases
Source: Nature. 2026 Sep 23;658(8138):1301–9. doi: 10.1038/s41586-026-11058-6 (PMC13716837; doi:10.1038/s41586-026-11058-6)
Supplement: Supplementary file 3 [file 41586_2026_11058_MOESM1_ESM.docx]

Supplementary material for:

**The anthropogenic fingerprint on emerging infectious diseases**

Rory Gibb^1,†^, Sadie J. Ryan^2,3,4†^, David M. Pigott^5^, Maria del Pilar Fernandez^6^, Renata L. Muylaert^7^, Gregory F. Albery^8^, Daniel J. Becker^9^, Jason K. Blackburn^3,10^, Hernan Caceres-Escobar^11^, Michael Celone^12^, Evan A. Eskew^13^, Hannah K. Frank^14^, Barbara A. Han^15^, Erin N. Hulland^12^, Kate E. Jones^1^, Rebecca Katz^16^, Adam Kucharski^17^, Direk Limmathurotsakul^18,19^, Catherine A. Lippi^2,3^, Joshua Longbottom^20^, Juan Fernando Martinez^21^, Jane P. Messina^22,23^, Elaine O. Nsoesie^24^, David W. Redding^25^, Daniel Romero-Alvarez^26,27^, Boris V. Schmid^28^, Stephanie N. Seifert^6^, Anabel Sinchi^29^, Christopher H. Trisos^30,31^, Michelle Wille^32^, and Colin J. Carlson^8,16,33,†^

**Affiliations**

^1^Department of Genetics, Evolution and Environment, University College London, London, UK.

^2^Quantitative Disease Ecology and Conservation (QDEC) Laboratory, Department of Geography, University of Florida, Gainesville, FL, USA.

^3^Emerging Pathogens Institute, University of Florida, Gainesville, FL, USA.

^4^College of Life Sciences, University of KwaZulu Natal, Durban, South Africa.

^5^Department of Health Metrics Sciences, University of Washington, Seattle, WA, USA.

^6^Paul G. Allen School for Global Health, Washington State University, Pullman, WA, USA

^7^Sydney School of Veterinary Science, The University of Sydney, Sydney, Australia.

^8^Department of Biology, Georgetown University, Washington, DC, USA.

^9^ School of Biological Sciences, University of Oklahoma, Norman, OK, USA.

^10^Spatial Epidemiology and Ecology Research (SEER) Laboratory, Department of Geography, University of Florida, Gainesville, FL, USA.

^11^Facultad de Medicina Veterinaria y Agronomía, Universidad de Las Américas, campus Providencia. Santiago, Chile

^12^Institute for Health Metrics and Evaluation, University of Washington, Seattle, WA, USA.

^13^Institute for Interdisciplinary Data Sciences, University of Idaho, Moscow, ID, USA.

^14^Department of Ecology & Evolutionary Biology, Tulane University, New Orleans, LA, USA.

^15^Cary Institute of Ecosystem Studies, Millbrook, NY, USA.

^16^Center for Global Health Science and Security, Georgetown University, Washington, DC, USA

^17^Centre for Mathematical Modelling of Infectious Diseases, London School of Hygiene and Tropical Medicine, London, UK.

^18^Mahidol-Oxford Tropical Medicine Research Unit (MORU), Faculty of Tropical Medicine, Mahidol University, Bangkok, Thailand.

^19^Centre for Tropical Medicine and Global Health, Nuffield Department of Medicine, University of Oxford, Oxford, UK.

^20^Department of Vector Biology, Liverpool School of Tropical Medicine, Liverpool, UK.

^21^Center for Integrated Earth System Information (CIESIN), Columbia Climate School, Columbia University, New York, NY, USA.

^22^School of Geography and Environment, University of Oxford, Oxford, UK.
^23^Oxford School of Global and Area Studies, University of Oxford, Oxford, UK.

^24^Department of Global Health, Boston University School of Public Health, Boston, MA, USA.

^25^Science Department, Natural History Museum,London, UK.

^26^Research Group of Emerging and Neglected Diseases, Ecoepidemiology and Biodiversity, Health Science Faculty, Universidad Internacional SEK (UISEK), Quito, Ecuador.

^27^Instituto Nacional de Biodiversidad (INABIO), Quito, Ecuador
^28^Infectious Disease Epidemiology, Department of Animal Sciences, Wageningen University & Research, Wageningen, Netherlands.

^29^Instituto Nacional de Enfermedades Virales Humanas Dr. Julio I. Maiztegui, Administración Nacional de Laboratorios e Institutos de Salud (ANLIS), Argentina.

^30^African Synthesis Centre for Climate Change, Environment and Development (ASCEND), University of Cape Town, Cape Town, South Africa.

^31^Climate Risk Lab, African Climate and Development Initiative, University of Cape Town, Cape Town, South Africa.
^32^Centre for Pathogen Genomics, Department of Microbiology and Immunology, at the Peter Doherty Institute for Infection and Immunity, University of Melbourne, Melbourne, Victoria, Australia.

^33^Department of Epidemiology of Microbial Diseases, Yale University School of Public Health, New Haven, CT, USA.

^†^These authors contributed equally and share corresponding author status.

Correspondence to Rory Gibb ([rory.gibb.14@ucl.ac.uk](mailto:rory.gibb.14@ucl.ac.uk)), Sadie Ryan ([sjryan@ufl.edu](mailto:sjryan@ufl.edu)) and Colin Carlson ([Colin.Carlson@yale.edu](mailto:Colin.Carlson@yale.edu)).

**Table of contents:**

1. Supplementary Table 1: Disease data sources, links and access.
2. Supplementary Table 2: Hypothesis generation exercise form as completed by study coauthors.
3. Supplementary Table 3: Socio-environmental driver data sources, links and access.
4. Supplementary Figure 1: Forest plots of linear fixed effects from disease-specific multivariable models.
5. Supplementary Figure 2: Modelling results using the “any author” hypothesis criterion.
6. Supplementary Figure 3: Modelling results using the “top ranked” hypothesis criterion.
7. Supplementary Figure 4: Correlations between covariates averaged across different buffer sizes.

**Supplementary Tables**
(Files are provided as separate Excel spreadsheets)

**Supp. Table 1:** **Disease data sources, links and access**. The table provides a full itemised list of all the data sources for each disease included in this study, including detail on the spatial and temporal extent, the specific sources collated to create the final dataset, and accessibility information for each dataset. Most disease datasets were open-access and public-domain, and processed versions of these are included in the study code and data repository. Others were accessed from national disease surveillance system data portals or via data requests to specific authors or groups; in these cases, we provide details of how to access or request each data source.

**Supp. Table 2:** Hypothesis generation exercise form. A blank version of the online form that was circulated to coauthors to contribute to the hypothesis generation exercise. Information on how this form was developed and tested is provided in the Methods.

**Supp. Table 3: Socio-environmental driver data sources, links and access.** This table provides a full itemised list of the covariate data sources for each disease, including a brief description of whether or how each was preprocessed, and web links to access the original datasets.

**Supplementary Figures**

**Supp. Figure 1: Forest plots of marginal effects of emerging disease drivers for 31 modelled diseases.** Points and error bars show the marginal effect (linear fixed effect) of z-score scaled driver covariates on outbreak event risk for each disease (posterior mean and 95% credible interval, log-odds scale). Each panel shows a modelled disease, and shows results for 3 separate models fitted including hypotheses generated using three criteria from the author exercise: either “majority rule”, “top ranked”, or “any author” (denoted by point colour).

**Supp Figure 2: Inferred drivers of outbreak risk for 31 emerging infectious diseases based on “top ranked” hypothesis criterion.** The figure replicates the results from main text Figures 3 and 4, but based on hypotheses generated using the stricter “top ranked” criterion (Extended Data Figure 5c). Top row (A): summary of effects across 31 diseases; see Figure 3 legend (main text). Middle and bottom rows (B-G): analysis of mean effect size and driver sharing across 31 diseases; see Figure 3 legend (main text).

**Supp. Figure 3: Drivers of outbreak risk for 31 emerging infectious diseases based on “any author” hypothesis criterion.** The figure replicates the results from main text Figures 3 and 4, but based on hypotheses generated using the broader “any author” criterion (Extended Data Figure 5c). Top row (A): summary of effects across 31 diseases; see Figure 3 legend (main text). Middle and bottom rows (B-G): analysis of mean effect size and driver sharing across 31 diseases; see Figure 3 legend (main text).

**Supp. Figure 4: Averaged covariate values are highly correlated across different buffer sizes.** To ensure the results would not be affected by differences in buffer size (spatial uncertainty) for each outbreak event, we tested the correlation between covariates averaged in a 5km buffer around 1000 randomly-generated points worldwide, and covariates averaged in 2.5, 10, and 20km buffers. The matrix colour shading shows the correlation (Pearson coefficient) between 5km and other buffer sizes. Almost all covariates were very highly correlated (coefficient > 0.9) over all buffer sizes.
